# Magnetic Nanoparticle Composites: Synergistic Effects and Applications

**DOI:** 10.1002/advs.202004951

**Published:** 2021-05-05

**Authors:** Stefanos Mourdikoudis, Athanasia Kostopoulou, Alec P. LaGrow

**Affiliations:** ^1^ Biophysics Group Department of Physics and Astronomy University College London London WC1E 6BT UK; ^2^ UCL Healthcare Biomagnetic and Nanomaterials Laboratories 21 Albemarle Street London W1S 4BS UK; ^3^ Institute of Electronic Structure and Laser (IESL) Foundation for Research and Technology‐Hellas (FORTH) 100 Nikolaou Plastira Heraklion Crete 70013 Greece; ^4^ International Iberian Nanotechnology Laboratory Braga 4715‐330 Portugal

**Keywords:** biomedicine, catalysis, nanoparticle synthesis, sensing, surface functionalization

## Abstract

Composite materials are made from two or more constituent materials with distinct physical or chemical properties that, when combined, produce a material with characteristics which are at least to some degree different from its individual components. Nanocomposite materials are composed of different materials of which at least one has nanoscale dimensions. Common types of nanocomposites consist of a combination of two different elements, with a nanoparticle that is linked to, or surrounded by, another organic or inorganic material, for example in a core‐shell or heterostructure configuration. A general family of nanoparticle composites concerns the coating of a nanoscale material by a polymer, SiO_2_ or carbon. Other materials, such as graphene or graphene oxide (GO), are used as supports forming composites when nanoscale materials are deposited onto them. In this Review we focus on magnetic nanocomposites, describing their synthetic methods, physical properties and applications. Several types of nanocomposites are presented, according to their composition, morphology or surface functionalization. Their applications are largely due to the synergistic effects that appear thanks to the co‐existence of two different materials and to their interface, resulting in properties often better than those of their single‐phase components. Applications discussed concern magnetically separable catalysts, water treatment, diagnostics‐sensing and biomedicine.

## Graphical abstract

Different compositions and morphologies of all‐inorganic or organic‐inorganic magnetic nanocomposites, synthesis routes aiming for new properties and improved performance in a range of fields. Applications in catalysis, sensing‐diagnostics, water treatment and biomedical domains. Evaluation of recyclability‐reusability, magnetic separability, efficiency and cost‐effective processing.

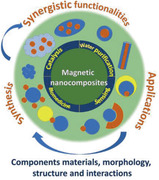

## 1 Introduction

Magnetic nanocomposite materials are made from two or more components with different physical or chemical properties that, when combined, form a material with distinct features, compared to their single constituent parts. Among these materials, at least one should have size in the nanometer range and at least one should be magnetic. Magnetic nanocomposites have drawn increased attention thanks to their remarkable properties. Numerous combinations of the composing units of nanocomposites exist, which involve magnetic nanoparticles (NPs) and a second component which is either organic or inorganic. The states of the nanocomposites can be colloidal, powder, fibers, membranes, or films.^[^ ^1^ ^]^ For example, carbon shells and graphene sheets combined with Fe_2_O_3_ nanoparticles have been reported to provide electrical networks that allow fast and efficient electron transport, when these composites are used as anode materials in Li‐ion batteries. Carbon coating around each individual iron oxide nanoparticle hinders the direct contact of electrode and electrolyte, thus endowing a large reversible specific capacity to the whole composite material.^[^ ^2^ ^]^ SiO_2_ is another material that is commonly used as a coating layer surrounding magnetic nanoparticles to form nanocomposites. Silica shells prevent the aggregation of the sole nanoparticles and chemical decomposition in solution.^[^ ^3^ ^]^ The shell protects the core from oxidation or attack by chemicals, and it can be further functionalized by attaching functional groups to its surface.^[^ ^4^ ^]^ Such optical, physical, chemical, or biomedical functional groups can be attached depending on the desired application, as drug delivery, bioseparation, and diagnostic analysis.^[^ ^5^, ^6^ ^]^ Another important family of magnetic nanocomposites (MNCs) are nanostructures with yolk–shell, Janus, or dimer–oligomer configurations as well as different types of core–shell structures. It has been demonstrated that the growth or deposition of a second material onto a core is usually non‐epitaxial when a large lattice mismatch exists between the lattice parameters of the two materials. Such process could cause symmetry breaking, another pathway that nanocrystals could undertake to evolve into new shapes, whereas a small lattice mismatch can often lead to epitaxial growth, resulting in alloy or well‐controlled core–shell structures, depending on the synthesis temperature.^[^ ^7^ ^]^ For instance, a Au shell on the surface of iron oxide NPs provides chemical stability and biocompatibility. Janus and dimer nanocomposite types attract also a significant amount of interest: Janus particles are symmetric in shape but asymmetric in surface properties due to the distribution of different functional groups over the particle surfaces.^[^ ^8^ ^]^ Heterodimer structures, such as Pt‐Fe_3_O_4_, are typically produced through seed‐mediated pathways and can be used in several applications.^[^ ^9^ ^]^ Recently, yolk–shell composites with a movable core inside a hollow capsule have gained significant interest due to their specific morphology. Such structures are often formed through the occurrence of the so‐called Kirkendall effect. Compared with core–shell composites, they display higher surface area, larger void space, and lower density, which results in their suitability for applications in drug delivery, lithium‐ion batteries, and catalysis;^[^ ^10^ ^]^ especially supported catalysis using magnetic components such as iron oxide nanoparticles enables the magnetic separation and recyclability of the nanocomposites. Other applications of MNCs include sensing, water purification, and biomedical fields. The inorganic metals which constitute the main part of MNCs can be also combined with a range of different organic, organometallic, biological, and other moieties, including metal–organic frameworks, in order to be suitable for a range of applications. Such organic surface functionalities may be provided during or after synthesis.

**Scheme 1:**
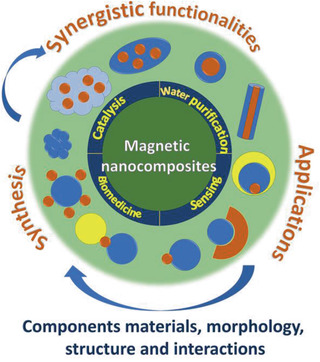
Different morphologies and configurations of all‐inorganic or organic–inorganic magnetic nanocomposites (the organic part is indicated by the light blue color). Synthesis routes lead to new properties and improved performance in applications.

In this review, we present in a comprehensive way the synthetic methods, the improved synergistic properties, and the range of applications of several categories of MNCs (**Scheme** **1**). In most cases, chemistry‐based techniques are employed for the preparation of MNCs, not excluding the additional use of other production methods, if needed, in certain stages of the overall fabrication process.

This paper is organized in different sections, which will present the principal families of magnetic nanocomposites and their applications. First, we present MNCs with inorganic components. Core–shell nanostructures with or without lattice matching, carbon coating, silica shells, and yolk–shell structures are described. Afterward, the synthesis and characterization of Janus as well as dimer and oligomer nanostructures are demonstrated. Inorganic–organic and inorganic–organometallic composites are then reported, with emphasis on the control of organic surface chemistry and biofunctionalization. Throughout the paper, focus will be given to the synergistic effects of the nanocomposite components, when possible, which endow enhanced properties and improved performance in applications. A special section will be dedicated to catalysis and the presentation of supported and magnetically separable catalysts. The applications of MNCs in water treatment will then be analyzed, followed by the demonstration of applications in biomedical domains such as hyperthermia, drug delivery, and magnetic resonance imaging (MRI). **Table** **1**provides relevant information on some characteristic examples of MNCs. The use of MNCs for diagnostic purposes will finally be explained, before the suggestion of future outlooks.

**Table 1:** Summary of the main information for some representative magnetic nanocomposites featured in this Review (the materials are listed according to the order of the corresponding figures in the text)

Type/Figure	Synthesis method	Applications	Reference
Fe_3_O_4_@Au core‐shell NPs / 1	Numerous different methods (see text)	Biomedicine, environment	^[^ ^17^ ^]^
Co@SoSb core‐shell NRs / 2	Metal‐organic growth of CoSb on pre‐synthesized Co NRs	Not reported yet	^[^ ^39^ ^]^
Cu@Ni core‐shell NWs / 3	Epitaxial growth of Ni shell onto Cu NWs	Not reported therein	^[^ ^50^ ^]^
Fe_3_O_4_@RF@void@mSiO_2_ yolk‐shell MNCs / 4b	Plasmolysis‐inspired nanoengineering strategy	Catalytic hydrogenation of 4‐nitrophenol	^[^ ^79^ ^]^
Ni@void@SnO_2_ yolk‐shell MNCs / 4c	Acid etching hydrothermal method	Outstanding electromagnetic wave absorption	^[^ ^94^ ^]^
Fe_3_O_4_@SiO_2_ core‐shell MNCs / 5a	Water‐in‐oil microemulsion	Magnetic particle imaging (MPI)	^[^ ^99^ ^]^
Amine‐functionalized Fe_2_O_3_‐SiO_2_ core‐shell MNCs / 6	Three‐step process	Biomedicine (envisaged)	^[^ ^114^ ^]^
Fe_3_O_4_@C MNCs / 7a	Solvothermal process	Several applications	^[^ ^141^ ^]^
Au‐Fe_3_O_4_ heterostructures / 8a	Growth of Fe_3_O_4_ in the presence of Au seeds	Biomedicine (envisaged)	^[^ ^185^ ^]^
Ag‐Pt‐Fe_3_O_4_ heterotrimers / 8b	Total‐synthesis framework (seeds‐based)	Several applications	^[^ ^188^ ^]^
Fe_2_O_3_‐based MNCs / 9	Ligand exchange and click chemistry	Not reported	^[^ ^243^ ^]^
Au/Fe_x_O_y_‐thiol, catechol Janus particles / 10	Multi‐step process	Biological applications, MRI	^[^ ^264^ ^]^
[RuCl(CO_3_)(*μ*‐DOPA)]@maghemite MNCs / 11	Multi‐step process	CO release	^[^ ^270^ ^]^
NCMTs@Fe_3_O_4_@SiO_2_@C/Ni MNCs / 12	Multi‐step process	4‐NP catalytic reduction	^[^ ^320^ ^]^
HAP@Fe_3_O_4_@PDMS paper /13	Multi‐step process	Oil/water separation‐wastewater treatment	^[^ ^368^ ^]^
Tea waste/Fe_3_O_4_ MNCs / 14	Co‐precipitation	Cr(VI) removal	^[^ ^370^ ^]^
Fe_3_O_4_@SiO_2_@Ag MNCs / 15	Multi‐step process	Thiram pesticide detection	^[^ ^415^ ^]^
GR‐Fe_3_O_4_‐PEDOT‐Au MNCs / 16a	Multi‐step process	Penicillin detection	^[^ ^419^ ^]^
Au@MWCNTs‐Fe_3_O4‐PtTi / 16b	Multi‐step process	Streptomycin detection	^[^ ^429^ ^]^
MBCPE/Fe_3_O_4_@Ag MNCs / 17	Multi‐step process	DNA detection	^[^ ^421^ ^]^
Au/Fe_3_O_4_/MoS_2_CAs MNCs / 18	Multi‐step process	Hg(II) detection	^[^ ^427^ ^]^
FeCo/GC MNCs / 19	Methane CVD	MRI	^[^ ^124^ ^]^
SPION‐PEG‐based MNCs / 20	Therm. Decomp. Synth. & functionalization	MRI	^[^ ^459^ ^]^
Mn‐SPIO‐loaded mPEG‐b‐PCL micelles / 21b	Therm. Decomp. Synth. & micelle formation	MRI	^[^ ^456^ ^]^
NaYF_4_:Yb^3+^,Tm^3+^@Fe_x_O_y_ MNCs / 23d	Two‐step synthesis & functionalization	MRI	^[^ ^496^ ^]^
Pd‐Fe_3_O_4_ Janus MNCs / 25	Two‐step synthesis	Magnetic‐photo dual‐mode hyperthermia	^[^ ^527^ ^]^
Iron oxide‐loaded hollow mSiO_2_‐DOX / 26	Multi‐step process	Drug delivery and hyperthermia	^[^ ^544^ ^]^
Polystyrene/Fe_3_O_4_@SiO_2_ Janus MNCs / 27	Multi‐step process	Drug release and tumor cell targeting	^[^ ^555^ ^]^
Fe_5_C_2_@Fe_3_O_4_ MNCs / 29	Two‐step process	Fe^2+^ release, ROS, MRI	^[^ ^558^ ^]^
Porous core‐shell hematite‐SiO_2_‐NiO MNCs / 30	Multi‐step process	Magnetic protein separation	^[^ ^566^ ^]^

## 2 Magnetic Nanoparticles with Inorganic Composites

One of the main categories of MNCs refers to those which have inorganic materials in both of their components. Multiple structures including core–shell configurations or Janus and dimer–trimer particles can be obtained by combining different materials. We begin this section by presenting the core–shell structures between different inorganic elements, alloys or oxides, while separate sub‐sections will be devoted to silica‐ and carbon‐coated as well as to yolk–shell composites.

### 2.1 Core–Shell Magnetic Nanocomposites

**Figure 1:**
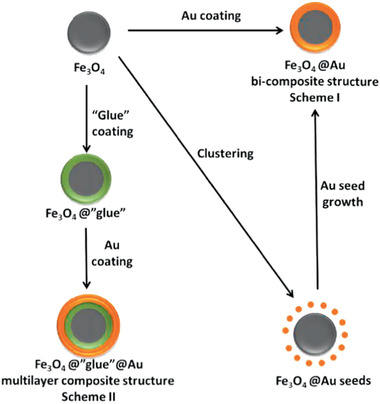
Schematic representation of three different strategies for the synthesis of Fe_3_O_4_@Au core/shell nanoparticles. Reproduced with permission.^[^ ^17^ ^]^ Copyright 2015, Elsevier.

Magnetite (Fe_3_O_4_) nanoparticles are largely used for biomedical and water treatment applications, among others. Sood et al. have produced Fe_3_O_4_/Au core–shell nanocomposites by inserting a mixture of gold chloride and trisodium citrate dihydrate solution in a moderately heated suspension of pre‐synthesized Fe_3_O_4_ NPs.^[^ ^11^ ^]^ The attachment of thiolated sodium alginate onto the surface of the composites provided an improved stability and rendered these materials as potentially suitable for drug delivery application with the added capability to be used as contrast agents for MRI. The gold shell protects the core from further oxidation and corrosion, while it exhibits good biocompatibility and affinity via amine/thiol terminal groups.^[^ ^12^ ^]^ Fe_3_O_4_/Au composites prepared using pre‐synthesized magnetite NPs showed good biosensing performance, with great potential in the field of protein detection and disease diagnosis.^[^ ^13^ ^]^ Terpyridine‐type ligand‐protected Fe_3_O_4_@Au‐L composites were prepared in a simple three‐step reaction. The advantages of that approach, which involved citrate reduction and ligand exchange, are simplicity, low reaction temperature, and high reproducibility.^[^ ^14^ ^]^ The near‐infrared (NIR) light sensitivity together with the strong adsorptive ability of gold surface and the uniqueness of magnetic NPs renders such composites easily manipulated and heated by an external magnetic field. Fouad et al. used such composites for the photodegradation of the organophosphate insecticide malathion. It was found that the presence of Au nanoshell increased the photodegradation efficiency in the presence of ultraviolet (UV) light and allowed the photodegradation even in natural sunlight since Au is a visible responsive material.^[^ ^15^ ^]^ Tamer and co‐workers reported the synthesis of anisotropic core–shell nanocomposites by dispersing Fe_3_O_4_@Au NPs in sodium citrate followed by their addition into a growth solution consisting of cetyl trimethylammonium bromide, HAuCl_4_, AgNO_3_ and ascorbic acid at room temperature. The high surface area of the product helped toward a very good performance in the detection of *Escherichia coli* in high volumes.^[^ ^16^ ^]^ Majouga and colleagues reviewed a range of typical approaches for the synthesis of Fe_3_O_4_@Au nanocomposites for biomedical applications (**Figure** **1**). Such wet‐chemistry synthesis routes include co‐precipitation, thermal decomposition, reverse micelle, water‐in‐oil inverse nanoemulsion, ultrasonic irradiation, and solvothermal reduction.^[^ ^17^ ^]^

In another work, a quick and simple one‐step method to synthesize Fe_3_O_4_@Au composites using pre‐synthesized Fe_3_O_4_ NPs and a heated HAuCl_4_ solution resulted in a nanoparticulate product with high sensitivity in the detection of nucleic acids in aqueous conditions.^[^ ^18^ ^]^ Huang and co‐workers have published a Review on the synthesis of spherical and anisotropic magnetic‐plasmonic core–shell NPs focusing on iron oxide‐gold system. The optical properties of the iron oxide‐gold core–shell composites can be controlled from the visible to the NIR region by changing the core size, shell thickness, as well as the core and shell shapes. The magnetic properties of the core–shell composites are the same as the ones of the single cores, with reduced saturation magnetization due to the mass contribution of the diamagnetic Au.^[^ ^19^ ^]^

The “inverse” configuration of the aforementioned system, that is, Au core@Fe_3_O_4_ shell nanocomposites, has also been reported. In particular, a systematic study of the latter system produced by thermal decomposition has been published by Morais and co‐workers.^[^ ^20^ ^]^ The occurrence of exchange anisotropy effect was demonstrated, caused by the interaction of a magnetically ordered layer in the magnetite shell and disordered spins located at the interface Au/magnetite and the external surface of the magnetite shell. Ghorbani et al. reported the synthesis of Au/Fe_3_O_4_ core/shell NPs which were surface decorated by a dual stimuli‐responsive copolymer of thiolated poly (N‐isopropylacrylamide‐*co*‐itaconic acid) (thiolated poly(NI‐PAAm‐*co*‐IA), suitable for application as a carrier of a well‐known anticancer agent, doxorubicin (DOX).^[^ ^21^ ^]^ Sun's group has published the synthesis of Fe_3_O_4_/Au and Fe_3_O_4_/Au/Ag composites by reducing HAuCl_4_ in a chloroform solution of oleylamine (OAm) in the presence of magnetite NPs. The plasmonic properties of these structures can be tailored by controlling the coating composition and thickness.^[^ ^22^ ^]^ In another work, Fe_3_O_4_@Ag nanocomposites were produced with the aim to construct a nontoxic biomimetic interface and develop a novel immobilization method for proteins.^[^ ^23^ ^]^

Ag‐Fe_3_O_4_ nanocomposites have been synthesized by a combination of electrochemical and reduction methods: in particular, the electro‐oxidation of iron in water was used, and Ag^+^ was reduced to Ag^0^ with glucose as reductant and NaOH as accelerator.^[^ ^1^ ^]^ In fact, magnetite nanoparticles have been used as cores to be coated also by other inorganic materials, apart from gold. For example, an uncomplicated and green approach to produce core–shell Fe_3_O_4_@C@MnO_2_ composites was reported. That approach contained two steps: C‐coated magnetite nanoclusters were synthesized via a solvothermal route, and then MnO_2_ nanosheets were decorated onto the surface of the Fe_3_O_4_@C through self‐sacrificing oxidation‐reduction reaction. The resulting nanocomposites had large surface area and were employed as adsorbents for U(VI) and Eu(III) elimination from wastewater.^[^ ^24^ ^]^ Composites composed of carbon nanotube (CNT) films decorated with Fe_3_O_4_/MnO_2_ were prepared by a two‐step approach using solvothermal deposition and electrochemical deposition.^[^ ^25^ ^]^ The magnetite NPs and the MnO_2_ sheets formed a nanoscale core–shell structure on the surface of the CNT film. The unique core–shell and 3D network morphology is deemed to be of benefit for the electromagnetic wave absorption capability of the composites because of the interfacial polarization and high specific surface area.^[^ ^25^ ^]^ In a different work, Angeloni et al. prepared Fe_3_O_4_@Cu core–shell NPs by a co‐precipitation method and characterized them with magnetic force microscopy (MFM). A so‐called “dipole model,” derived by the way that the MFM images are acquired due to the tip‐NP interaction, was used to estimate the thickness of the coating of the core–shell composites.^[^ ^26^ ^]^ Wang and co‐workers reported the eco‐friendly synthesis of Fe_3_O_4_@MoS_2_ and MoS_2_@Fe_3_O_4_ composites by replacing hydrazine hydrate used in previously published protocols with ammonium hydroxide. The products were used for Cr(VI) removal under several different experimental conditions (solution pH, Cr(VI) concentration, adsorbent dose, contact time, temperature).^[^ ^27^ ^]^

Zerovalent Fe core NPs have also been coated by Au shells, already in 2005 by the Kauzlarich group. A reverse micelle method was used for their synthesis. The Au shell appeared to grow by nucleating at selected sites on the Fe core surface before coalescing. The resultant Au shell had a rough surface, which could compromise its oxidation resistance.^[^ ^28^ ^]^ Furthermore, Fe@Au NPs were fabricated through a microemulsion process and then grafted with methotrexate and indocyanine green. The composites were used to kill cancer cells by applying magnetic hyperthermia and releasing the anti‐cancer methotrexate.^[^ ^29^ ^]^ Huber and colleagues have presented the gram‐scale synthesis of strongly magnetic Fe/Fe_x_O_y_ core–shell NPs and used them as the magnetic fraction in the formation of a matrix‐free superparamagnetic nanocomposite. The particles were produced via a scalable reversible agglomeration mechanism, taking part in a solvothermal reaction. An oxide layer was formed, with no increase in its thickness over time, thus showing its passivating abilities for the Fe(0) core.^[^ ^30^ ^]^ However, Famiani et al. showed that for ligand exchange to aqueous media, corrosion of the central Fe(0) core occurred, and thus a larger core size was needed if only the native oxide shell was being used to passivate the particles.^[^ ^31^ ^]^ Yang et al. prepared iron carbide nanoparticles by introducing heteroatoms at body‐centered‐cubic (*bcc*) Fe@Fe_3_O_4_ NPs. A void space between the Fe_2_C core and the magnetite shell was observed, which acted as the possible reactive area in the carbon penetration process, implying the Kirkendall effect through the lattice transition. Halide (hetero‐Cl) ions made the phase transformation take place in a kinetically controlled manner, because their attachment on the Fe_3_O_4_ shell somehow interferes with the carbon penetration paths and causes the C atoms diffusion occur in a controlled way.^[^ ^32^ ^]^ The Chaudret group reported a versatile low‐temperature chemical synthesis of core–shell iron/iron carbide NPs which were air‐stable after some initial losses and showed excellent magnetic properties with tailored magnetic anisotropy and very good hyperthermia performance.^[^ ^33^ ^]^ Moreover, Qi et al. prepared several iron/iron carbide/carbon‐based core/shell/shell composites by controlling the pyrolysis temperature in a water‐assisted, environmentally benign and inexpensive chemical vapor deposition method. The introduction of water vapor had a significant effect on the yield and morphology of the produced materials. These composites displayed a promising prospect as high‐performance microwave absorbing materials.^[^ ^34^ ^]^

**Figure 2:**
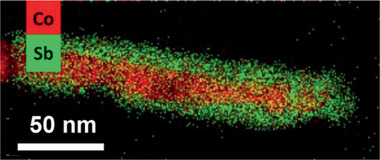
HAADF‐STEM image with EDS chemical mapping of the Co@CoSb nanorods. Reproduced with permission.^[^ ^39^ ^]^ Copyright 2016, American Chemical Society.

Another magnetic element that can be coated in the nanoscale with gold (and other) shells is cobalt. The gold coating can provide additional functionality, such as sensitivity to optical probes and other biomolecules. Terrones and co‐workers produced N‐MWCNT (nitrogen‐doped multi‐walled carbon nanotubes) containing Co@Au core–shell NPs in their tips. This composite material could be used as a magnetoplasmonic system and the CNT acted as drug delivery component taking advantage of its large surface area and biocompatibility. The combination of ferromagnetic and superparamagnetic‐like behaviors of these core–shell NPs made them attractive for applications in nanomedicine, where they can be used to target or destroy cancer cells. A high vacuum deposition chamber was needed as a part of the required equipment for the fabrication of the aforementioned composite material.^[^ ^35^ ^]^ Already in 2007 Krishnan and co‐workers had reported the preparation of Co‐Au core–shell NPs by the combination of a mild reductant (OAm) with a relatively stable gold precursor [(C_6_H_5_)_3_P]AuCl.^[^ ^36^ ^]^ A sensitive magnetic method to monitor the formation of core–shell Co‐Au NPs by measuring the hysteresis (recording coercivity and remanence values) as a function of reaction time during the progress of their synthesis was published by Krishnan group.^[^ ^37^ ^]^ Baaziz et al. published a work on the liquid‐phase filling of CNT with magnetic Co‐based NPs of uniform morphology. Such composite could be applied in the fields of magnetism, catalysis, battery, electrochemical devices, or wastewater treatment.^[^ ^38^ ^]^ Viau and colleagues reported the synthesis of Co@CoSb nanorods by reducing an antimony salt at the surface of cobalt nanorods in a mixture of 1,2‐tetradecanediol and OAm (**Figure** **2**). The presence of the CoSb shell helped to prevent the sintering of the cobalt core and preserve shape anisotropy and magnetic properties.^[^ ^39^ ^]^

Thanh and co‐workers synthesized CoFe@Pt core–shell NPs through a wet‐chemical “hot‐injection” method, and the composites where transferred into water with the help of the amphiphilic poly(maleic anhydride‐*alt*‐1‐octadecene) (PMAO).^[^ ^40^ ^]^ Physical vapor nanoparticle‐deposition approaches can be also used, apart from the colloidal chemistry routes, for the preparation for core–shell NPs. A PVD method was used by Kline et al. to produce high‐moment Fe(Co)‐Au core–shell NPs. The product was water soluble and biocompatible and was evaluated as a heating agent material for magnetic hyperthermia.^[^ ^41^ ^]^ Already in 2005, Bai and Wang had reported a physical technique combining an on‐line sputtering/evaporation process with an integrated nanocluster deposition process to prepare core–shell‐type FeCo‐Au and FeCo‐Ag core–shell NPs. The products were superparamagnetic with high magnetic moment.^[^ ^42^ ^]^ Fe‐ and FeCo‐based composites with remarkable magnetic properties were prepared after depositing such NPs under soft‐landing and ultra‐high‐vacuum conditions on bare tungsten (W(110)) surface.^[^ ^43^ ^]^

**Figure 3:**
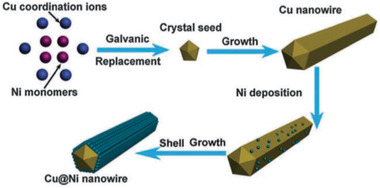
Schematic demonstration of the formation mechanism of corn‐like Cu‐Ni core–shell nanowires. Reproduced with permission.^[^ ^50^ ^]^ Copyright 2016, Royal Society of Chemistry.

Maenosono and co‐workers have reported the synthesis of Ag/FeCo/Ag core/shell/shell NPs via a hot injection method in combination with a polyol method. These particles became water soluble after modifying their surface with a hydrophilic polymer, *ε*‐poly‐_L_‐lysine (PLL). It was suggested that Ag cores acted as a catalyst to reduce Co^2+^ and the Co was required to reduce Fe cations to form the FeCo shell. These multishell composites enabled real‐time monitoring of the motion of liposomes by plasmonic imaging, and they show potential to be utilized as magnetic probes for the separation of intracellular vesicles.^[^ ^44^ ^]^ A partial oxidation of the FeCo intermediate shell can result in the observation of exchange bias due to the interface between ferromagnetic FeCo and antiferromagnetic Co_x_Fe_1‐x_O, but in general, the Ag, apart from its surface plasmon resonance property, helps to suppress oxidation of the FeCo alloy shell via electron transfer. It also offers a surface which is easy to modify through metal‐thiol interactions.^[^ ^45^ ^]^ Vasilakaki et al. have published a few works on the Monte Carlo simulations of such ferromagnetic/antiferromagnetic (FM/AFM) core–shell systems which present exchange bias and enhanced magnetic properties.^[^ ^46^ ^]^ Exchange bias was also demonstrated at FM/AFM core–shell Co_x_Fe_1‐x_O@Co_y_Fe_3‐y_O_4_ NPs prepared by the group of Begin‐Colin. It was shown that the presence of oleic acid (OAc) as ligand facilitated the formation of the core–shell configuration.^[^ ^47^ ^]^ Magnetic behavior similar to those noticed in magnetic thin film multilayers was shown for CoFe_2_O_4_/FeCo‐FeO core–shell NPs as reported by Nigam and co‐workers. The structure of those composites was presumably formed by a ferromagnetic cobalt ferrite core and a FM‐AFM shell of FeCo‐FeO. The composite shell coupled to the core by the exchange‐spring mechanism combining the high magnetic anisotropy of the ferrimagnetic core having the large magnetic moment of the FM FeCo with the unidirectional anisotropy of the AFM FeO. The strong magnetocrystalline anisotropy of the cobalt ferrite core ensured the strong exchange coupling at the core–shell interface.^[^ ^48^ ^]^ Zacharaki et al. prepared surfactant‐free Ni/Al_2_O_3_ model catalysts by first synthesizing Ni colloids before their deposition on alumina. 6–8 nm sized supported NPs with a core–shell structure were evidenced.^[^ ^49^ ^]^ In another report, Cu‐Ni core–shell NPs and nanowires were prepared by using Cu seeds and growing a Ni shell onto them in an epitaxial manner, in the presence of Ni(acac)_2_ and OAm. The Ni shell can improve the anti‐oxidation properties of copper nanomaterials. A growth along the <100> directions was observed for the Ni shell, parallel to the <100> directions of the Cu (**Figure** **3**). This was assigned to the lattice match between both elements and the minimization of the interface free energy. Selectively exposed surfaces and morphologies were generated with tailored magnetic properties.^[^ ^50^ ^]^ In a different approach, CoFe_2_O_4_‐CdS core–shell structures were recently prepared with a chemical precipitation synthesis route. The effect of the nanocomposites along with rice husk ash microparticles on the mechanical properties of epoxy resin was studied. Upon introduction of both types of particles, the tensile strength showed an ascending trend, attributed to the interparticle interactions of the NPs, the rice husk ash particles, and epoxy fibers. The interaction among the epoxy fibers was improved.^[^ ^51^ ^]^

A more complex core–shell structure has been synthesized by Wang et al.: A new self‐assembly method was used to produce Ni‐doped CeO_2_@NiO core–shell composites. NiO nanosheets root into self‐assembled Ni‐CeO_2_ microspheres, enabling the nickel oxide nanosheets to be firmly contacted with Ni‐CeO_2_, which helps the adsorption and mobility of oxygen and carbon monoxide. These materials displayed a greatly increased catalytic performance on CO oxidation. Synergistic effects between Ni‐doped cerium dioxide and nickel oxide nanosheets were considered as responsible for such high catalytic activity.^[^ ^52^ ^]^ Flower‐like CoS_2_@MoS_2_ core–shell microspheres coated by reduced graphene oxide were prepared by a facile hydrothermal method. The produced composites were found to act as promising materials as lightweight, broadband, tunable and high‐performance microwave absorbers. A low filler loading can reduce the density of absorbers, which is crucial to improve their practical value when applied in MW adsorption.^[^ ^53^ ^]^ Apart from the “hot‐injection” approach, where precursors are directly injected at a heated solution, the “heat‐up” method has also been used for the preparation of core–shell composites, as described in a Review by van Embden et al. Precursors with differing reactivities can be used to synthesize core–shell composites for systems that do not favor alloying.^[^ ^54^ ^]^ Younan Xia and co‐workers have reviewed the synthesis of nanoparticles by switching from batch to droplet reactors. The latter reactors facilitate the continuous and scalable synthesis of nanoscale materials with tuned features. Core–shell composites including metal–metal, oxide–oxide, or semiconductor–semiconductor configuration have already been obtained through seed‐mediated growth in droplet reactors.^[^ ^55^ ^]^

The presence of lattice mismatch between a shell and a core material may affect the way that the shell will be grown onto the core. For example, it is reasonable to understand that magnetite (Fe_3_O_4_), a more oxidized form of iron oxide, compared to wustite (FeO), has a small lattice mismatch (3%) with it. It would be difficult to anticipate a larger mismatch in the course of a naturally progressive oxidation process, when a shell is composed of higher oxidation states, whereas an interior surface would be composed of the less oxidized FeO.^[^ ^56^ ^]^ In fact, the theoretically expected lattice mismatch between two materials may not be the same with the experimentally observed one. For example, Cabot and co‐workers calculated a cell spacing difference between a Fe_3_O_4_ core and a NiFe_x_O_y_ shell at around 4%, although a difference of only 1% in lattice parameters between both structures should be present. This difference was attributed to the possible existence of a core–shell structure with a magnetite core and a more oxidized, defective maghemite shell.^[^ ^57^ ^]^ In another work, a 4.4% lattice mismatch was observed in Fe_3_O_4_/CdS core–shell composites synthesized at room temperature in aqueous medium. This mismatch concerned the (111) planes of the magnetite and the (111) planes of the *fcc* CdS. In that composite, HRTEM imaging indicated a heteroepitaxial growth mode. The non‐negligible lattice mismatch was the reason behind the induced changes in the optical properties of the composite, as indicated by red shifts in the absorption onset.^[^ ^58^ ^]^ Robinson et al. prepared Co‐Au and Fe_3_O_4_‐Au core–shell composites through wet‐chemical routes and they assigned the apparent distortion on nanoparticle surfaces to curvature effects. In this manner, the shell materials, despite having large lattice mismatch, can be in principle grown onto the core surface in an epitaxial way.^[^ ^59^ ^]^ In the Fe‐Au system, a smaller lattice mismatch between the iron core and the gold shell should be expected when the gold grows in certain orientations in respect to iron. In a paper reported by O'Connor and colleagues, the Fe core grew as sphere‐like shape inside reverse micelles; therefore, the epitaxial growth of Au could not be perfectly extended to a 3D manner. In that case, a different orientation growth or mismatch could arise in the course of the formation of the Au shell.^[^ ^60^ ^]^

Interestingly, a mismatch of only 0.7% between the FeCo (200) and Au (220) planes was observed in FeCo‐Au core–shell nanocomposites prepared with a sputtering‐based approach. Au (100) was epitaxially grown on the top of FeCo (100) with a 45^o^ rotation. Twinning was spotted between the Au epitaxially grown on two neighboring FeCo planes. Such composites are deemed to be promising for biomedical applications, thanks to the presence of the biocompatible Au shell.^[^ ^61^ ^]^ A highly strained shell, with a clear interface between the core and the shell was observed in FePt@Fe_3_O_4_ core–shell composites prepared by reduction of Pt(acac)_2_ and thermal decomposition of Fe(CO)_5_. The strain observed in the oxide shell seemed to be due to the interfacial lattice mismatch which was estimated to be 23%. The lattice strain induced in the shell by the core helps for applications in mass transport devices in catalysis and nanoscale multiferroic domains.^[^ ^62^ ^]^ Liu and co‐workers managed to synthesize FePt@ZnO core–shell composites with a seed‐mediated route in a quasiepitaxial growth manner. Also in that case, some uncommon emissions of the ZnO were attributed to the lattice mismatch at the interface of the two materials because of the possible presence of impurity states in the bandgap, which could provoke emissions at higher wavelengths.^[^ ^63^ ^]^ The large lattice mismatch between a magnetic core and a semiconductor shell will usually complicate the synthesis of magnetic‐fluorescent core–shell composites destined for bioapplications. Despite this, tetraethylene glycol (TEG) was used as a solvent and reducing agent for the synthesis of FePt@CdSe core–shell composites with sequential addition of precursors and no parallel generation of dimers was shown. Superparamagnetic properties with increased magnetization as well as fluorescent behavior was observed in those composites.^[^ ^64^ ^]^

Wen and Krishnan suggested that in Au‐Co core–shell NPs synthesized through a wet‐chemical approach, the cobalt spins at the Au/Co interface can be pinned by the strain due to the lattice mismatch of gold and cobalt. The Au/Co interface possessed a big degree of disorder due to multiple nucleation sites during heterogeneous nucleation.^[^ ^65^ ^]^ In fact, not always a large lattice mismatch can cause big difficulties in the epitaxial growth of one metal onto another. For example, Tsuji et al. observed an epitaxial growth of Ni shells on Au cores despite a large lattice mismatch (13.6%). In the case of CdSe grown on nickel nanoparticle, Siah et al. showed that epitaxial islands of CdSe could be formed on Ni even though there was a large lattice mismatch between those materials: a single‐crystalline shell could be formed when the particle size was below 11 nm.^[^ ^66^ ^]^ Still, the conformal epitaxial growth may not take place even in systems with smaller lattice mismatch, for which a heterogeneous nucleation and island growth mode can also occur (e.g., the Au@Pt system). This implies that a lattice mismatch below 5% is not a definitive factor to assure epitaxial growth. In general, a large lattice mismatch between two elements would lead to a large interfacial stress in the pseudomorphic state, which can be relieved by mechanisms as atomic relaxations, generation of misfit dislocations, or interfacial mixing of two metals. Kinetic factors also govern the epitaxial growth in large mismatch systems such as the Au@Ni. It seems that slow reduction of metallic cations keeps the concentration of metal atoms below supersaturation which is a requisite for epitaxial growth in large lattice mismatch core–shell composites.^[^ ^67^ ^]^ In another study, non‐epitaxial growth was presented for Au‐CdS core–shell composites. In that system, the lattice mismatch between the two majority lattice planes of bulk Au and CdS is up to 43%. Still, no strain‐induced bond‐length shifts compared with bulk‐indexed peaks were detected by XRD, which is different from other epitaxially grown core–shell composites with much smaller lattice mismatches.^[^ ^68^ ^]^ Maenosono and co‐workers have reported that the natural oxidation of FeCo cores results in oxidized surfaces with increased lattice constant, which hinders the epitaxial growth of a Ag shell onto them. On the contrary, producing Ag@FeCo core–shell NPs was much easier as silver is more resistant to oxidation than FeCo and the lattice constants do not get modified.^[^ ^69^ ^]^ Meng et al. have observed that a large lattice mismatch between Pd cores and AuCu shells is a key point for the generation of branches under slow reduction kinetics in a seed‐mediated growth route. In addition to the kinetic control, the ≈4% lattice mismatch between the Pd core and the AuCu shell contributed to the anisotropic growth mode. The capping effect of the surfactants used (cetyltrimethylammonium chloride) on the {100} facets of Pd cubes also favored the growth of AuCu in a branch mode. The produced Pd@AuCu planar tetrapods were found as promising for use in surface‐enhanced Raman scattering (SERS).^[^ ^70^ ^]^ Of course, in many cases, a large lattice mismatch over 10% will hinder the epitaxial growth, as in the case of PbS grown on the top of Cu_2_S nanoparticles. In that case, Cu_2_S‐PbS heteronanostructures will be favored instead of core–shell composites.^[^ ^71^ ^]^ It has to be mentioned that Xia and co‐workers have published a review on the seed‐mediated growth of metal nanocrystals, where all the queries and key points related to the role of lattice mismatch and other parameters on the layer‐by‐layer epitaxial or anisotropic growth of two‐component systems are described in detail.^[^ ^6^ ^]^

From the above, one can notice that the seed‐mediated growth is a dominant approach to produce numerous different core–shell nanocomposites, destined for a range of applications, when a precursor is decomposed to grow a shell onto pre‐synthesized core particles. The generated shell can endow new properties, such as biocompatibility and further possibilities for surface functionalization, according to its surface groups present. Issues such as the lattice mismatch between the different components have to be considered when attempting to produce core–shell nanocomposites.

### 2.2 Yolk–Shell Magnetic Nanocomposites

Yolk–shell nanocomposites are a particular case of core–shell nanostructures where an interior void is located between the core and the shell. Lin et al. have used SiO_2_ as the sacrificial template for the synthesis of magneto‐plasmonic Fe_3_O_4_@Au yolk–shell NPs. Their synthetic method included the coating of magnetite core with silica interlayer and porous gold outer shell, followed by removing the silica template. These composites showed good performance for multimodal MR/PA/PET imaging and NIR light‐induced chemothermal synergistic therapy.^[^ ^72^ ^]^ A silica templating method was also utilized to produce yolk–shell Fe_3_O_4_/carbon double‐layered shell, with plenty of Au NPs in situ immobilized in the shell. The yolk–shell structure prevents aggregation of the neighboring cores and protects the core from the outside environment while allowing the diffusion of small active molecules into and out of the shell, thus facilitating catalytic activity. The superparamagnetic magnetite part helps the recycling of the composite through applying an external magnetic field while the outer C layer offers additional adsorption sites for the attachment of Au NPs and protects the Fe_3_O_4_ layer from external harsh conditions. These composites acted as excellent catalysts for the reduction of 4‐nitrophenol (4‐NP) to 4‐aminophenol (4‐AP).^[^ ^73^ ^]^ Yolk–shell TiO_2_‐based composites were prepared via successive sol–gel coating on Fe_3_O_4_ particles, followed by annealing. The magnetite particles were initially synthesized by solvothermal method and the final composites demonstrated an excellent performance in the heterogeneous catalysis of styrene epoxidation, with high selectivity toward styrene oxide.^[^ ^74^ ^]^ Magnetic mesoporous carbon composites with a yolk–shell structure were fabricated with an in situ carbonization strategy, including stages of sol–gel assembly, oxidation, sulphonation, carbonization, and etching. The cavity of the yolk–shell structure provides additional space for storage of targeted molecules in order to offer an enhanced storage capacity to the composite. Those composites were employed as adsorbents for selective extraction of low abundance endogenous peptides from human serum.^[^ ^75^ ^]^ Lu et al. developed a simple method via chemical etching to produce yolk–shell Fe_3_O_4_@C composites as a multifunctional biosensing platform for the label‐free colorimetric detection of H_2_O_2_ and glucose.^[^ ^76^ ^]^ A facile method based on hydrothermal synthesis was employed for the production of Fe_3_O_4_@graphene yolk–shell nanocomposites. These materials were highly dispersible in water, with superparamagnetic behavior and high loading capacity for DOX.^[^ ^77^ ^]^

**Figure 4:**
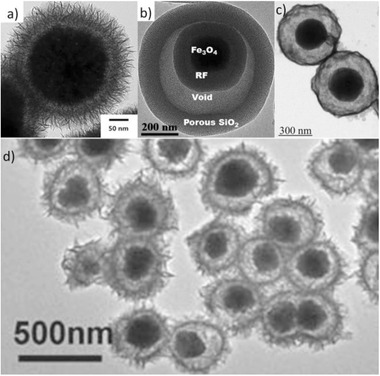
TEM images of different yolk–shell nanocomposites: a) Fe_3_O_4_@SiO_2_@Co_3_O_4_, b) Fe_3_O_4_@RF@void@mSiO_2_, c) Ni@void@SnO_2_, d) SPIO@nickel silicate. a) Reproduced with permission.^[^ ^78^ ^]^ Copyright 2015, Springer. b) Reproduced with permission.^[^^79^^]^ Copyright 2017, American Chemical Society. c) Reproduced with permission.^[^^82^^]^ Copyright 2011, Wiley‐VCH. d) Reproduced with permission.^[^^94]^ Copyright 2016, American Chemical Society.

Yolk–shell multi‐shell Fe_3_O_4_@SiO_2_@Co_3_O_4_ composites (**Figure** **4**a) were produced with a three‐step procedure by Chen and co‐workers. These materials demonstrated a high catalytic performance in photocatalytic water oxidation. The growth of Co_3_O_4_ NWs on the silica surface and the role of silica electronic barrier with wide band gap to avoid charge recombination helped toward such good catalytic activity.^[^ ^78^ ^]^ In another work, a facile plasmolysis‐inspired nanoengineering strategy was elaborated for the controlled production of yolk–shell Fe_3_O_4_@resorcinol‐formaldehyde(RF)@void@mSiO_2_ (mesoporous silica) microspheres (Figure 4b). The superparamagnetic core with considerable magnetization and a very high surface area (439 m^2^ g^−1^) were two of the most important features of these composites, which showed an excellent performance in the catalytic hydrogenation of 4‐NP.^[^ ^79^ ^]^ Silica etching and using polyaniline (PANI) formed by in situ polymerization as a template for the synthesis of Ni@Pd core–shell NPs were among the main steps of the synthesis of yolk–shell Fe_3_O_4_@PANI/Ni@Pd composites as shown by Niknezhad and co‐workers. The outer shell of yolk–shell composites typically needs to have both a high surface area and good dispersion characteristics in order to act as a catalyst support, thus maximizing the catalytic activity of the metal NPs. The composites were efficient for the selective catalytic reduction of nitrobenzenes.^[^ ^80^ ^]^ Yao et al. showed a facile way to fabricate Fe_x_O_y_/Pd@mSiO_2_ composites with a movable magnetic core loading inside mSiO_2_ capsules and dispersed Pd NPs anchoring on the inner surface. These composites were considered as ideal nanoreactors for heterogeneous catalytic reactions, and they were tested for the 4‐NP reduction reaction in the presence of NaBH_4_.^[^ ^10^ ^]^ The same group published also the synthesis of Fe_x_O_y_/PdPt/CeO_2_ yolk–shell composites, where a movable Fe_x_O_y_ core was encapsulated in a mesoporous CeO_2_ shell with PdPt alloys embedded in the surface of the inner shell. The composites were used for the reduction of the methylene blue (MB) dye and the magnetic core helped for their easy recovery and reusability.^[^ ^81^ ^]^ In another work, iron oxide@nickel silicate yolk–shell composites (Figure 4d) were synthesized with a simple method using silica in a sacrificial templating process. Gold NPs with tuned amount could be immobilized into the above composites by an in situ reducing process. The Au‐loaded yolk–shell composites were efficient for the catalytic reduction of rhodamine B (RhB).^[^ ^82^ ^]^ Fang et al. prepared Fe_2_O_3_‐CeO_2_/Au/carbon yolk–shell ellipsoid composites with ultrafine Au NPs. To prepare the composites, a one‐step surfactant‐free extended Stober process combined with carbonization–hydrothermal etching approach was employed. Then, [Au(en)_2_]^3+^ species used as gold precursors were mediated within the yolk–shell ellipsoids using the deposition–precipitation method. The final composites showed a very good performance as recoverable catalysts toward the reduction of 4‐NP and organic dyes.^[^ ^83^ ^]^ The same authors published also a combinatory approach for the synthesis of double‐shell hollow magnetic ultra‐small Au‐loaded yolk–shell ellipsoids (Fe@MO_2_‐Au@H‐SiO_2_). The initial Fe_2_O_3_@MO_2_ (M is Ce or Ti)/mSiO_2_ materials were produced through a facile bottom‐up assembly process based on sol–gel reactions. Also in that case, a deposition–precipitation method was used to encapsulate the Au NPs within the shell structures and the resulting materials catalyzed the reduction of 4‐NP to 4‐AP.^[^ ^84^ ^]^

Furthermore, yolk–shell composites consisting of a movable magnetic Fe core and metal oxide shell decorated with Pt‐Au NPs were prepared by an in situ reduction method followed by calcination in air and reduction in dihydrogen gas atmosphere. The incorporation of TiO_2_ in the Pt‐Au nanocatalyst helped for a much better catalytic performance in comparison to the insertion of either ZrO_2_ or m‐SiO_2_. Synergistic effects at the interface between the titania support and Au were suggested to occur, and in that case, the TiO_2_ would not only act as an inert carrier but would also intervene in the catalytic process itself. The model reaction of the reduction of 4‐NP to 4‐AP was tested.^[^ ^85^ ^]^ Gao et al. prepared FePt@Fe_2_O_3_ yolk–shell nanocomposites which displayed high cytotoxicity assigned to the FePt yolks and MR contrast enhancement thanks to the Fe_2_O_3_ shells. The Kirkendall effect played an important role for the formation of these composites, where FePt NPs were used as seeds.^[^ ^86^ ^]^ In another report, the production of yolk–shell spheres composed of a movable magnetic core, a chitosan‐derived porous carbon shell, and plenty of tiny Cu_core_Ag_shell_ NPs confined within the porous shell, was described. The preparation of these composites involved first the solvothermal synthesis of magnetite clusters and their silica coating with a sol–gel process. Selective silica etching and Cu@Ag NP immobilization on the surface of the composites followed, and the final product was used for the epoxidation reaction of styrene in water at room temperature. The role of chitosan as a functional, non‐toxic, and biocompatible agent with strong affinity for transition metals, which modified the surface of the Fe_3_O_4_@SiO_2_ NPs, was highlighted.^[^ ^87^ ^]^ Au@Fe_3_O_4_ nanocomposites have been synthesized by Lin and Doong through the decomposition of iron pentacarbonyl in the presence of 2.5–10 nm Au core NPs. These authors comment that although the template synthesis is effective for the production of yolk–shell composites, it is often a multistep, complex, and time‐consuming process. For this reason, they pursued a more facile method based on the Kirkendall effect and using Au NPs as the core materials. The Fe_3_O_4_ shells protected the excellent catalytic performance of the Au core for the reduction of nitroarene, and the interior hollow cavity served as an ideal platform for this reaction. Catalytic reduction reactions of 4‐NP and 1‐chloro‐4‐nitrobenzene were also investigated for these composites.^[^ ^88^ ^]^

Cobalt nanoparticles have also been used as main components of yolk–shell composites. Du and co‐workers have published the metal organic chemical vapour deposition (CVD) synthesis of Co/nanoporous carbon/void@carbonyl iron yolk–shell composites. A subsequent calcination step took place, too. The authors state that the in situ generation of void layer, coming from the shrink of a cobalt‐based zeolitic imidazolate framework (ZIF) during carbonization, offers distinct advantages in comparison to the common template method. The composites possessed a dielectric/magnetic media heterostructure with multiple interfaces and exhibited a broad effective absorption bandwidth. That work illustrated the capability of carbonyl iron as an excellent light absorber.^[^ ^89^ ^]^ In another report, the synthesis of Ag‐CoO heterostructures and Ag/Co‐CoO yolk–shell composites was described. This method involved the reduction of silver nitrate in the presence of cobalt NPs in OAm. The Kirkendall effect plays an important role for the gradual oxidation of Co‐CoO core–shell NPs to yolk–shell structures and finally to hollow CoO NPs. Such composites are regarded as suitable for applications in catalysis, drug delivery, and SERS.^[^ ^90^ ^]^ A facile strategy to incorporate yolk–shell CoO@Co and ZnO NPs with graphene sheets has been developed by Zhu et al. Hydrothermal annealing and wet‐chemical steps were included. The Kirkendall effect was also in that case crucial for the fabrication of these composites. These lightweight materials displayed excellent electromagnetic wave absorption properties.^[^ ^91^ ^]^ Another paper presents the production of yolk–shell Co‐C/void/Co_9_S_8_ ternary composites composed of a Co NP‐embedded porous carbon core and Co_9_S_8_ shell. This synthesis included the sulfidation of a Co‐based ZIF followed by pyrolysis. The very good microwave (MW)‐absorbing activity of the composites was attributed to the large interfacial polarization effect originating from abundant heterointerfaces, the synergistic effect between magnetic and dielectric components, and the tunable cavity between core and shell.^[^ ^92^ ^]^ In fact, a seminal paper on the synthesis of Pt@CoO yolk–shell composites based on the Kirkendall effect had already been published in 2004, using Pt NPs as seeds.^[^ ^93^ ^]^ Yolk–shell Ni@SnO_2_ nanocomposites (Figure 4c) were produced by a low‐cost and simple acid etching hydrothermal method, showing outstanding electromagnetic wave absorption performance. The void between Ni and SnO_2_ induced multiple reflection and scattering under alternated electromagnetic field. It also enabled to tailor complex permittivity to obtain proper impedance match, which makes the microwaves penetrate into absorbing materials to the maximum.^[^ ^94^ ^]^ Another report presented the wet‐chemical synthesis of CeO_2_‐MO_x_ yolk–shell nanocomposites (“M” refers to Cu, Co, or Ni). To achieve this, as‐synthesized CeO_2_ was solvothermally treated with M(CH_3_COO)_2_ in ethanol solution. The good dispersion of MO_x_ and the close contact between CeO_2_ and MO_x_ helped toward an improved catalytic activity in CO oxidation.^[^ ^95^ ^]^ In fact, a lengthy review paper on the classifications, synthesis, properties, and application of yolk–shell NPs was published by Purbia and Paria.^[^ ^96^ ^]^

Summarizing this sub‐section, one can observe that for the yolk–shell configuration, silica templating is a common fabrication method which has been partially replaced more recently by more simple approaches, for example, based on the Kirkendall effect. In yolk–shell structures, aggregation between different cores is prevented, while the diffusion of small active molecules is still possible, which is beneficial for catalytic performance. The abundant heterointerfaces can lead to particularly interesting properties, such as large interfacial polarization.

### 2.3 Silica‐Coated Magnetic Nanocomposites

**Figure 5:**
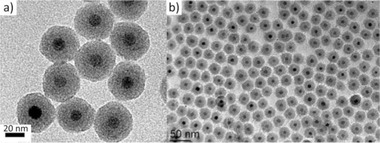
TEM images of iron oxide@silica core–shell nanoparticles. a) Reproduced with permission^[^ ^99^ ^]^ Copyright 2012, American Chemical Society. b) Reproduced with permission.^[^ ^108^ ^]^ Copyright 2010, Springer.

SiO_2_ shell on the surface of several types of nanoscale materials is a widely used component of nanocomposites. For instance, regarding magnetic NPs, silica shell helps to prevent their aggregation, promotes their thermal and chemical stability, and decreases their risk of toxicity, while it also improves their microwave absorption property. Saeedirad et al. proposed a simple method based on magnetic attraction to decorate untreated MWCNTs by silica‐coated magnetite NPs. The SiO_2_ coating of the Fe_3_O_4_ NPs was achieved by using a common Si precursor, tetraethyl orthosilicate (TEOS), and the final Fe_3_O_4_‐SiO_2_‐MWCNT nanocomposites were evaluated in what concerns their MW absorption properties.^[^ ^97^ ^]^ In another work, Fe_3_O_4_ NPs were produced by co‐precipitation method and then coated with silica, again using TEOS as Si source. The composites were used for the isolation of DNA of Hepatitis virus type B (HBV) and Epstein–Barr virus from several real serum samples.^[^ ^98^ ^]^ A very reproducible protocol for the production of Fe_3_O_4_@SiO_2_ composites (**Figure** **5**a) for different core sizes and with tuned size thicknesses was reported by Ding et al. The authors used a reverse microemulsion approach in cyclohexane, in the presence of Igepal, ammonium hydroxide, and TEOS.^[^ ^99^ ^]^ A modified sol–gel method with relatively short duration was described to obtain uniform Fe_3_O_4_/SiO_2_ core–shell nanocubes. In that work, the considerable magnetization, the biocompatibility, and the affinity of these composites for binding biomolecules, make them of potential interest for several biosensing applications.^[^ ^100^ ^]^ The non‐toxic silica shell protects the Fe_3_O_4_ core from corrosive media, and it does not alter its crystal and local structures (interatomic distances, degree of structural irregularity, coordination numbers). However, the absolute magnetization value of the nanocomposite decreases with the increase of the SiO_2_ shell thickness.^[^ ^101^ ^]^

A modified Stober method was employed for the synthesis of Fe_3_O_4_@SiO_2_ nanocomposites using 20 nm hydrophilic Fe_3_O_4_ NPs as seeds. The “common” Stober method is the alkaline hydrolysis of TEOS. The modified Stober reported by Hui et al. avoided the use of any surfactant. The silica shell was amorphous and its thickness could vary between 12.5 and 45 nm by tuning the ratio of TEOS/Fe_3_O_4_ or the reaction time.^[^ ^102^ ^]^ Fe_3_O_4_‐SiO_x_ core–shell nanocomposites are studied for their applications in biomedicine, for example, as T_2_ (dark) contrast enhancement agents in MRI. The influence of silica shell thickness on its *r_2_* relaxivity in MRI was investigated by Dravid and colleagues. In that work, the magnetite cores were prepared by chemical decomposition method, while the microemulsion process was employed for the silica coating.^[^ ^103^ ^]^ In another report, Congo red magnetic composites (Fe_3_O_4_@SiO_2_‐CR) were prepared under mild conditions, with stages including co‐precipitation, Stober method, silanization, and nucleophilic substitution reaction. The composites were employed to remove MB dye from wastewater. The excellent adsorption activity for MB was due to the strong electrostatic interactions between the dye and Fe_3_O_4_@SiO_2_‐CR.^[^ ^104^ ^]^ Kalantary and co‐workers synthesized Fe_3_O_4_ NPs with co‐precipitation method and after silica coating they used the resulting composites to remove humic acid (HA) from aqueous solutions. The SiO_2_‐coated magnetic nanocomposites showed a better HA removal performance in comparison to the “naked” magnetic NPs.^[^ ^105^ ^]^ A comparative study between the silica and polydopamine (PDA) coating of Fe_3_O_4_ NPs in what concerns their usage in bio‐separation platforms was published by Sahin et al. The initial magnetite NPs were prepared by co‐precipitation method. It was shown that PDA coating is a one‐step approach, with controlled shell thickness and superior magnetic behavior as well as biological modification ability but it proves complicated to control the level of size polydispersity with this approach. Silica coating, on the other hand, ensured monodisperse nanocomposites, when a high concentration of TEOS was used. Still, it has to be noted that SiO_2_ coating was a two‐step method, as it required an additional modification step for further application in bio‐separation.^[^ ^106^ ^]^

Zirak et al. prepared Fe_3_O_4_@SiO_2_ composites by chemical co‐precipitation in alkaline solution, followed by silylation using triethoxy silane, and then by treatment with 3‐aminopropyltriethoxysilane. They further coated their composites with carboxymethyl cellulose (CMC) and applied them to remove MB dye from water solutions. Abundant adsorption sites, easy separation with the use of an external magnetic field and low cost made these composites attractive for the aforementioned application (MB removal).^[^ ^107^ ^]^ In general, the hydrolyzed silica can provide silanol groups which facilitate surface biofunctionalization. Optical, physical, chemical, or biomedical functional moieties can be attached onto the silica shell in order to permit applications in drug delivery, bioseparation, and diagnostic analysis. Size‐controlled Fe_3_O_4_@SiO_2_ nanocomposites prepared by a one‐pot water‐in‐oil microemulsion route have been considered as suitable tracer materials for magnetic particle imaging (MPI) applications.^[^ ^5^ ^]^ Using an inverse microemulsion method, the silica thickness could be tuned in the range 5–13 nm by Vogt et al. In their case, OAc‐capped superparamagnetic Fe_3_O_4_ cores (Figure 5b) were transferred from oil phase to water phase via a ligand exchange process which was monitored by FTIR measurements.^[^ ^108^ ^]^ Binnemans and co‐workers published the synthesis of Fe_3_O_4_@SiO_2_(TMS‐EDTA) with homogeneous thin silica shells and highly superparamagnetic magnetite cores. The initial silica‐coated magnetic particles were prepared with a modified Stober method and they were then functionalized with N‐[(3‐trimethoxysilyl)propyl]ethylenediamine triacetic acid (TMS‐EDTA). The hydrophilic composites constitute a promising sorbent material for the selective recovery of rare‐earth ions from dilute aqueous solutions.^[^ ^4^ ^]^ Besides, Fe_3_O_4_@SiO_2_@polyionene/Br_3_^–^ composites were produced with a co‐precipitation method. These composites were employed as catalysts for the synthesis of imidazole and thiazole derivatives. The catalysts were easily recovered by simple magnetic decantation and could be recycled for several times without important reduction of their catalytic performance.^[^ ^109^ ^]^

**Figure 6:**
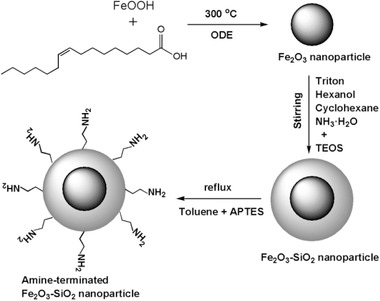
Schematic presentation of the synthesis of amine‐functionalized core–shell Fe_2_O_3_‐SiO_2_ magnetic nanocomposites. Reproduced with permission.^[^ ^114^ ^]^ Copyright 2018, IEEE Publishing.

A facile immobilization technique was used for the preparation of sulfonic acid‐supported silica coated magnetic composites (Fe_3_O_4_@SiO_2_@PrSO_3_H). Low‐cost precursors were utilized for this synthesis, where the silica‐coated magnetic NPs reacted with (3‐mercaptopropyl)trimethoxysilane to provide the final composites. This material acted as an efficient, magnetically separable, reusable, and environmentally friendly catalyst for the Pechmann condensation of substituted phenols with ethyl acetoacetate resulting in the formation of coumarin derivatives.^[^ ^110^ ^]^ Jiaqi et al. prepared Fe_3_O_4_@SiO_2_‐EDA‐COOH composites through the addition of different groups in pre‐synthesized Fe_3_O_4_@SiO_2_ (i.e., 3‐chloropropyltriethoxysilane, ethylenenediamine, maleic anhydride, and triethylamine). The composites were used for MB removal from water, and they showed higher adsorption capacity than other reported adsorbents, together with fast magnetic separation.^[^ ^3^ ^]^ A different approach has been used for the fabrication of magnetic/fluorescent composites by Abou‐Hassan and co‐workers: they employed a network of continuous‐flow microreactors for the microfluidic multistep synthesis of core–shell *γ*‐Fe_2_O_3_@SiO_2_ nanocomposites. Their synthesis products were not different in comparison to the ones obtained in the conventional batch synthesis, but they were fabricated in much shorter time.^[^ ^111^ ^]^ In another report, superparamagnetic *γ*‐Fe_2_O_3_ NPs were modified with two silica precursors (TEOS and aminosilanes). Cytotoxicity and in vitro immunotoxicity of *γ*‐Fe_2_O_3_@SiO_2_‐NH_2_ composites were minimal, but further assessments of this material were deemed as necessary before allowing its applications in the fields of cell labeling, MRI, and drug delivery.^[^ ^112^ ^]^ Makrygenni et al. published the synthesis of amine‐functionalized *γ*‐Fe_2_O_3_@SiO_2_ composites which were further covalently grafted with polyoxometalates. The nanocomposites were produced via the coupling of heteropolytungstate‐based hybrids bearing carboxylic acid functional groups with aminopropyl functional groups that decorate the APTES‐functionalized core–shell NPs. The composites displayed very good hyperthermic properties and they are considered also as promising for catalysis.^[^ ^113^ ^]^ APTES is a common reagent for the amine‐functionalization of iron oxide‐SiO_2_ nanocomposites. As illustrated in **Figure** **6**, surface functionalization of Fe_2_O_3_@SiO_2_ nanocomposites can take place through the silanization of aminosilane.^[^ ^114^ ^]^ Shao et al. highlight that the hydroxyl functional groups of SiO_2_ enable also to host more easily drug carrier molecules or to be coated with biomolecules (enzymes, antibodies, DNA, etc.) and sugars (dextrans, starch, albumin, and others).^[^ ^115^, ^116^ ^]^

Some additional positive features of SiO_2_ have been mentioned by Dobson and co‐workers, which are related to its optical transparency, heat‐resistance, low specific gravity, and good mechanical strength. Apart from being biocompatible, silica provides a negative surface charge in physiological pH, thus imitating biological species.^[^ ^117^ ^]^ Wu et al. have published a review paper on the synthesis and surface functionalization pathways of iron oxide NPs. A sub‐section of their paper is dedicated to silica coating where it is also mentioned that another approach to achieve such coating for Fe‐oxide NPs is the aerosol pyrolysis.^[^ ^118^ ^]^ The SiO_2_ shells provide an electrically insulating layer that reduces energy loss and additionally hinders the possibility of a decrease in permeability due to Fe oxidation. Hsieh et al. evaluated in vitro the viability and cytotoxicity of SiO_2_@Fe/SiO_2_ composites. These composites were considered able to act as dual‐functional agents in hyperthermia and as chemo agent carriers too.^[^ ^119^ ^]^ It has been observed that iron oxide@SiO_2_ composites can be converted to Fe@SiO_2_ through reduction with CaH_2_, maintaining the overall morphological characteristics of the starting particles.^[^ ^120^ ^]^

The room‐temperature synthesis of superparamagnetic FeCo@SiO_2_ nanocomposites was described by Desautels et al. The intimate contact between the metal core and the silica shells resulted in the spontaneous formation of metal silicates, as shown by XPS measurements.^[^ ^121^ ^]^ Another approach which combined co‐precipitation synthesis and H_2_ reduction led to the preparation of FeCo@SiO_2_ nanocomposites. These composites were considered as promising high‐frequency materials. In fact, the performance of compacted samples in high frequency depended strongly on the silica content.^[^ ^122^ ^]^ In another report, FeCo alloy NPs were supported on a 3D cubic mesoporous silica matrix (SBA‐16). A wet impregnation method was used for the matrix with a solution of Fe‐ and Co‐ nitrates. Afterward, calcination in air and reduction in dihydrogen flow comprised the next steps of the fabrication procedure for the nanocomposite. EXAFS and XANES techniques were employed, apart from XRD, to investigate the formation of the *bcc* FeCo through studying the intermediate products before reduction.^[^ ^123^ ^]^ An impregnation route of mixed Fe and Co salts in a methanol solution was used to load Fe and Co species on high‐surface area silica powder. Carbon deposition on FeCo‐SiO_2_ composites was carried out with a methane CVD step to produce MRI and near‐infrared agents.^[^ ^124^ ^]^ An urea‐assisted sol–gel protocol was used to prepare FeCo‐SiO_2_ aerogel nanocomposites. The structure of the FeCo NPs was studied, also in that case, with EXAFS and XANES spectra at the Fe and Co *K* edges. Surface oxidation, mainly consisting of iron oxide, was corroborated by electron energy loss spectroscopy analysis.^[^ ^125^ ^]^

Vanadium doping in FeCo‐SiO_2_ nanocomposites resulted in a remarkable modification of their temperature‐dependent magnetic properties, such as coercivity and exchange bias. In that case, the co‐reduction of metal halides was used for the formation of FeCo NPs. The generation of a mixed interfacial layer of iron and cobalt metal silicates was noticed for both undoped and V‐doped composites.^[^ ^126^ ^]^ A fast atom beam (FAB) sputtering technique has also been employed to co‐sputter Fe and Co foils along with a SiO_2_ target in a high vacuum chamber. In this way, FeCo‐SiO_2_ nanogranular composite films were deposited on a Si substrate. The enhanced field emission and SERS properties of those composites were studied through a combined experimental and theoretical work, involving XPS measurements as well as density functional theory and molecular dynamics simulations.^[^ ^127^ ^]^ In another work, FeCo nanocubes capped by polyvinylpyrrolidone (PVP) could be further coated with a thin layer of silica, maintaining their cubic shape. After amine‐functionalization, the binding of the nanocubes to a model sensor platform, such as carboxylic‐acid‐terminated self‐assembled monolayers, was illustrated.^[^ ^128^ ^]^ Condensed (not mesoporous) silica coating has been reported to protect the reactive FeCo alloy from oxidation in temperatures up to 300 °C.^[^ ^129^ ^]^

Bai and co‐workers synthesized CoFe_2_O_4_ NPs in ethylene glycol, and after silica coating, they further functionalized the resulting materials with thiol groups. The final CoFe_2_O_4_@SiO_2_‐SH nanocomposites were used for removal of Hg(II) from water, showing a promising efficiency.^[^ ^130^ ^]^ Cobalt‐zinc ferrite and magnetite NPs coated with SiO_2_ were used for the magnetic extraction and purification of DNA. The latter material binds to silanol and siloxane groups in the presence of salts such as NaCl, among others. Silica encapsulation hindered the oxidation of magnetite NPs to maghemite.^[^ ^131^ ^]^ Co‐SiO_2_ nanocomposites were prepared with a Co(NH_3_)_6_Cl_3_ template method in a polyoxoethylene‐nonylphenyl ether/cyclohexane reversed micelle system with a subsequent in situ reduction in water medium containing NaBH_4_/NH_3_BH_3_. The composites demonstrated a good catalytic activity for the hydrolysis of ammonia borane, showing recyclability and reusability.^[^ ^132^ ^]^ In another work, 3‐aminopropyl‐trimethoxysilane (APS) played an important role together with TEOS for the controlled synthesis of Co/SiO_2_ core–shell nanocomposites. CdS nanocrystals were then deposited onto the above structures, forming magnetic luminescent Co/SiO_2_/CdS nanocomposites. However, the deposition of CdS onto the SiO_2_‐coated Co did not occur in a homogeneous way.^[^ ^133^ ^]^

Kim et al. reported the synthesis of highly stable and magnetically recyclable Pt nanocatalysts in mesoporous silica embedded with FeCo/graphitic carbon (GC) NPs. CVD method and common silane molecules were used in this process, while the Pt immobilization was achieved by reduction of K_2_PtCl_4_ with ethanol. The final Pt‐FeCo/GC@mSiO_2_ displayed an excellent ability to catalyze cyclohexene hydrogenation, possessing high surface area and large pore volume.^[^ ^134^ ^]^ Gold NPs were immobilized onto the surface of FePt@SiO_2_ composites. A solution of gold NPs, which were formed by citrate reduction method, was mixed with amine‐functionalized FePt@SiO_2_ to provide the final composite. This material showed rapid SERS detection and identification of small biomolecules and microorganisms.^[^ ^135^ ^]^ In another report, a simple and reproducible hierarchically porous core–shell CaSO_4_/Fe_2_O_3_‐SiO_2_ composite was presented. The material demonstrated a very good catalytic activity for one‐pot transformation of crude *Jatropha curcas* oil to biodiesel.^[^ ^136^ ^]^ Guerrero‐Martinez et al. have published a review on silica coating which covers coating of pre‐synthesized or in situ synthesized NPs, as well as other aspects of this topic.^[^ ^137^ ^]^ Another review paper by Sun and co‐workers on the organic phases syntheses of magnetic NPs contains a short sub‐section on silica coating.^[^ ^138^ ^]^

Therefore, SiO_2_‐coated magnetic nanocomposites are often produced through the Stober approach, using one of its versions. TEOS is a common Si source, and the resulting silica shell helps to hinder the aggregation of the magnetic cores, while it also boosts their thermal and chemical stability and reduces their risk of toxicity. The hydroxyl functional groups of SiO_2_ enable also to host more easily drug carrier molecules. Silica provides a negative surface charge in physiological pH, imitating biological species. Optical transparency, heat resistance, low specific gravity, and good mechanical strength are additional benefits. Abundant adsorption sites, ability for easy magnetic separation, and low cost endow additional possibilities for application of SiO_2_‐coated magnetic nanocomposites in removal of organic dyes from water.

### 2.4 Carbon‐Coated Magnetic Nanocomposites

**Figure 7:**
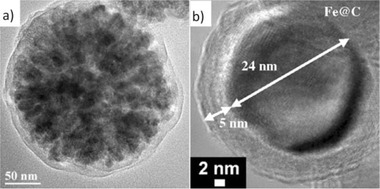
a) Fe_3_O_4_@C nanocomposites formed through a solvothermal method and b) Fe@C MNCs by high pressure CVD. Reproduced with permission.^[^ ^141^ ^]^ Copyright 2015, Elsevier. Reproduced with permission.^[^ ^151^ ^]^ Copyright 2009, Elsevier.

Carbon coating has also gained a significant amount of attention among researchers who try to improve the functionality of their magnetic composites. Wang et al. prepared core‐satellite and dumbbell‐like Fe_3_O_4_@C‐Ag composites. The process included a solvothermal synthesis of the Fe_3_O_4_@C NPs, followed by in situ reduction of silver ions in water at room temperature. The carbon shell promoted the adsorption and stabilization of Ag^+^ ions. In addition, the porous C shell acted as a physical barrier to hinder the aggregation between the particles. In what concerns biological applications, the hydrophilic carbon‐protected composites can be of interest as potential drug carriers for simultaneous imaging diagnosis and drug therapy. Those composites showed high catalytic recyclable performance for the degradation of RhB and it was stated that metal–carbon interfaces contributed in a positive way in the catalytic process.^[^ ^139^ ^]^ In another report, Fe_3_O_4_@C nanocomposites were produced via a combination of solvothermal, polymerization, and calcination steps. The carbide shell was considered as beneficial for potential applications in drug delivery, bioseparation, sensing, and other fields.^[^ ^140^ ^]^ Shi et al. synthesized Fe_3_O_4_@C nanocomposites (**Figure** **7**a) with a solvothermal method, using glucose, phenolic, and soluble starch resin as carbon source. The use of starch as carbon source resulted in the strongest intensity of diffuse characteristic peaks of carbon at the XRD patterns. The thickness of carbon shell could be tuned through adjusting reaction time and reactant ratios. The stability of the C shell allowed the protection of the magnetite core and their easy recyclability renders these composites as promising for several applications.^[^ ^141^ ^]^ The CVD strategy has also been used to prepare carbon‐coated Fe_2_O_3_ NPs dispersed on graphene sheets. When the Fe_2_O_3_@C@G composites were used as anode materials in Li‐ion batteries, the carbon coating helped to stabilize the solid electrolyte interface by avoiding direct contact of the iron oxide nanoparticles with the electrolyte. The C shell improved cycle stability by lowering the volume change during charge–discharge operations and prevented side reactions between electrode and electrolyte. The carbon coating helped also to ameliorate the rate capability thanks to its increased electrical conductivity, and in general both C and graphene roles were crucial for the outstanding performance of the Fe_2_O_3_@C@G electrode.^[^ ^2^ ^]^

Amine‐functionalized UiO‐66 composites with core–sell structures (*γ*‐Fe_2_O_3_@C@UiO‐66‐NH_2_) were produced through a simple, easy, and environmentally benign method consisting of co‐precipitation, hydrothermal, and solvothermal reaction stages. Amorphous carbon protects the cores against acids, bases, and solvents. The adsorption behavior of these composites for four hydrophilic dyes (MB, malachite green, acid fuchsin, and acid orange II) was investigated.^[^ ^142^ ^]^ Apart from preventing corrosion, the carbon coating offers an effective oxidation barrier for magnetic core materials. Its hydrophilic nature provides higher dispersibility and stability compared to the case of naked iron oxide NPs. Bae et al. prepared co‐precipitated ferrite NPs coated with C using a hydrothermal approach, and they demonstrated the suitability of the final composites as both T_1_ and T_2_ contrast agents in MRI.^[^ ^143^ ^]^ The carbon coating can be achieved also by using a sputtercoater with a carbon evaporation head, as in the case of gold‐magnetite heterostructure composites prepared by Krystofiak et al. These composites were further conjugated with protein for application in cell targeting.^[^ ^144^ ^]^ Cobalt ferrite NPs prepared by a reverse micelle method were afterward coated with carbon shells through a thermal CVD approach, using acetylene gas (C_2_H_2_) as carbon source.^[^ ^145^ ^]^ In a somewhat different approach, C‐coated and Fe‐doped *a*‐ΤiO_2_ nanocomposites were produced by using a one‐step flame combustion method, which was deemed as cost‐effective. The C coating and Fe‐doping of these titania NPs is expected to increase their ability to absorb more light in the visible range.^[^ ^146^ ^]^

Fe@C core–shell nanocomposites suitable for MRI were prepared via a one‐step top‐down approach through the electric plasma discharge generated in the cavitation field in organic solvents by an ultrasonic horn: ultrasound in toluene creates acoustic cavitations, which is the formation and implosive collapse of bubbles. The collapse of cavitation bubbles in a strong electric field between electrodes can polarize the *π*‐electrons in toluene, thus forming plasma. The production of NPs in the presence of plasma is then described.^[^ ^147^ ^]^ Chaudhary et al. fabricated carbon‐encapsulated iron NPs by plasma after applying ultrasonication in toluene. It was noted that no iron carbide phase formation was observed between the Fe core and the carbon shell. That plasma method was relatively inexpensive and those nanocomposites were considered as promising for MRI and photothermal cancer therapy.^[^ ^148^ ^]^ In a different approach, Fe_3_C@C composites were prepared by carbonizing Fe_3_O_4_‐lignin clusters, which were generated via a facile hydrogen bonding interaction between Fe‐OH and hydroxyl groups of lignin. A surface plasmon resonance (SPR) sensor was fabricated for selective and sensitive prior protein detection using aptamer‐modified Fe_3_C@C (Fe_3_C@C‐aptamer) as a recognition and amplification material for the strengthening of the SPR signal. High quantification and qualification ability for the protein was evidenced in that report.^[^ ^149^ ^]^ The thermal decomposition of cyclopentadienyl iron dicarbonyldimer [(C_5_H_5_)_2_Fe_2_(CO)_4_] resulted in carbon‐encapsulated Fe NPs attached to MWCNTs. Higher synthesis temperature led to higher degree of graphitization. The composites showed good potential for the removal of RhB from water. Electrospinning into aligned nanocomposite fibers provided a soft magnetic composite with potential applications in sensing and in fast switching solenoids.^[^ ^150^ ^]^ Apart from Fe‐based NPs, high pressure CVD has been shown to enable the C coating of Co and Ni NPs and an example of the formed composites is shown at Figure 7b.^[^ ^151^ ^]^ Kotoulas et al. presented an easy and inexpensive method to produce Co@C nanocomposites through a solvothermally assisted polyol route. Raman and HRTEM measurements confirmed the carbon coating and their magnetic hyperthermia performance was studied.^[^ ^152^ ^]^

The in situ pyrolysis of Prussian blue analogues (PBAs) provoked the formation of FeCo/C nanocomposites. The graphitization degree of the carbon frameworks was influenced by the Fe/Co ratio.^[^ ^153^ ^]^ Wang et al. used FeCo PBAs as nucleation sites for the polymerization of dopamine, to produce FeCo PBAs@PDA. A subsequent high‐temperature pyrolysis step generated FeCo@C@carbon nanocages. The authors state that a high carbon content favors a higher relative complex permittivity and dielectric loss through conductivity loss and dipole orientation polarization. On the other hand, high C amount induced moderately decreases relative complex permeability and magnetic loss.^[^ ^154^ ^]^ FeCo NPs coated by graphene have been synthesized through a catalytic CVD of methane in a continuous flow microreactor at atmospheric pressure. These composites showed high supercapacitor performance.^[^ ^155^ ^]^ A one‐step, solvent‐free approach to produce FeCo@C nanocomposites was reported by the thermolysis of low‐cost Fe(acac)_2_ and Co(acac)_2_ precursors in a closed cell under the autogenic pressure of the reactants.^[^ ^156^ ^]^ A simple solvothermal method was described for the synthesis of PtCo nanodendrites supported on N‐doped reduced graphene oxide (RGO), with linagliptin as structure‐directing agent and nitrogen dopant for the RGO. These composites were evaluated for their catalytic activity on 4‐NP reduction.^[^ ^157^ ^]^ Ye and colleagues have published a green mild hydrothermal carbonization technique for the preparation of FeNi@C nanocomposites. The metal precursors were FeSO_4_.7H_2_O and NiSO_4_.6H_2_O. In particular, FeNi NPs were first produced before being mixed with an aqueous solution of glucose and hydrothermally treated to 160 °C for 3.5 h to provide the final product.^[^ ^158^ ^]^

So, in what concerns MNCs with C‐coating, we conclude that the carbon shell can act as a physical barrier to prevent particle agglomeration and corrosion, but it also fights oxidation of the magnetic cores. One of the most common approaches to form the C coating is the CVD approach using molecules such as methane or acetylene gas as carbon source. The hydrophilic nature of carbon shell is beneficial for applications in imaging‐diagnosis and drug therapy. The enhanced electrical conductivity of carbon is advantageous for electrocatalytic applications, such as in anode materials for lithium ion batteries. Additional applications such as degradation of organic dyes have also been reported.

### 2.5 Janus and Dimer Magnetic Nanocomposites

Janus particles (named after the two‐faced Roman god Janus) are considered as a special type of patchy particles with only one patch that covers half of the particle. Patchy particles are defined as particles with one or more well‐defined patches, displaying strongly anisotropic and directional interactions. The particles possess a repulsive core and highly interactive surfaces. Modifying the surface chemistry or the shape of a spherical particle can help to generate a patchy particle. Several types of Janus particles exist, such as metal–metal, metal–dielectric, metal–polymeric, and non‐metallic nanocomposites, with either spherical or non‐spherical morphology.^[^ ^159^ ^]^ Composite Janus particles with enhanced fluorescence on one hemisphere were successfully prepared by a self‐organized precipitation method. These composites were also magnetic and contained Au NPs, Fe_3_O_4_ NPs, and 4‐(dicyanomethylene)‐2‐methyl‐6‐(*p*‐dimethylaminostryryl)‐4H‐pyran (DCM). Such composites have good potential as point light sources in optical circuits. This technology could find applications in nanolasers, strong fluorescence probes and sensors in the fields of photonics and medicine.^[^ ^160^ ^]^ Xi et al. prepared magnetic‐photoluminescent bifunctional Janus nanofibers by electrospinning technology using a homemade parallel spinneret. At the final [Fe_3_O_4_/PVP]/[NaYF_4_:Eu^3+^/PVP] nanofiber composites, one strand nanofiber was composed of Fe_3_O_4_ NPs and PVP, and the other one consisted of NaYF_4_:Eu^3+^ NPs and PVP. The average diameter of each strand of the Janus nanofiber was ≈600 nm.^[^ ^161^ ^]^ The electrospinning technology was also used to produce Janus nanofiber composites which contained a Fe_3_O_4_/PVP core and a Eu(BA)_3_phen/PVP shell as a half side with luminescent‐magnetic bifunctionality and polyaniline (PANI)/PVP nanofiber as the other half side with electrically conductive functionality.^[^ ^162^ ^]^ A one‐step, scalable flame aerosol technology was utilized to produce biocompatible, silica‐coated, Janus‐like Ag/Fe_2_O_3_ nanocomposites. The potential of these hybrid magneto‐plasmonic composites was studied by labeling their surface and specifically binding them on the membrane of tagged Raji and HeLa cells.^[^ ^163^ ^]^

A two‐step thermolysis‐based approach was used to prepare dumbbell‐like MnFe_2_O_4_‐NaYF_4_ Janus nanocomposites. MnFe_2_O_4_ NPs were produced first through a thermal decomposition method. Then the dumbbell structure was generated through epitaxial growth of lanthanide cation (Yb or Er)‐doped NaYF_4_ on the MnFe_2_O_4_ NPs to endow upconversion luminescence property. The magnetic‐luminescent composites combined optical property and high photothermal efficiency.^[^ ^164^ ^]^ Magnetic‐fluorescent bifunctional Janus nanofiber composites were fabricated through an electrospinning approach, also for the case of CoFe_2_O_4_‐polyacrylonitrile (PAN)/1,8‐napthalene anhydride (NAD)‐PVP system.^[^ ^165^ ^]^ A seed‐mediated growth approach was published by Liz‐Marzan and co‐workers for the synthesis of Au‐Fe_3_O_4_ nanocomposites. These magneto‐plasmonic materials showed high versatility as contrast agents in multimodal imaging and SERS detection.^[^ ^166^, ^167^ ^]^ Lattuada and Hatton used a so‐called masking technique to prepare water‐soluble 20 nm Janus magnetic nanocomposites by grafting polystyrene sodium sulfonate or polydimethylamino ethylmethacrylate to the exposed surfaces of negatively charged poly(acrylic acid) (PAA)‐coated Fe_3_O_4_ NPs adsorbed onto positively charged silica beads. The Janus particles were released from the silica beads by changing the pH.^[^ ^8^ ^]^ An emulsion‐free method using asymmetric silica/polystyrene Janus template was used to produce magnetic gold nanobowls. The template was covered with small iron oxide@Au NPs. The biosensing and drug delivery activity of the nanobowls was assessed through SERS detection of RhB and 4‐mercaptobenzoic acid.^[^ ^168^ ^]^ An electrospraying approach was used to prepare magnetic‐fluorescent bifunctional [PLGA/EuLa_3_(Bim)_12_]//[PLGA/Fe_3_O_4_] Janus microspheres (PLGA = polyactide‐*co*‐glycolide, Bim = benzimidazole]. Such composites are promising for drug delivery, biological imaging and bioprobe field applications.^[^ ^169^ ^]^ Pd‐coated magnetite‐polymers composites were fabricated using an emulsion method with a capillary tube‐based microfluidic device. To achieve this, an external field‐provoked migration of magnetic NPs that drag and stretch polymer chains along the field direction was employed.^[^ ^170^ ^]^ Zhang et al. reported a simple and inexpensive method to produce Fe_3_O_4_‐mesoporous silica Janus composites through a one‐step protocol. TEOS was used as silica precursor in a modified sol–gel process. The easy surface modification of these composites facilitates their applicability in bio/chemo molecule targeting and cellular labeling.^[^ ^171^ ^]^ Several review papers have discussed Janus composites: Walther and Muller described their synthesis, self‐assembly, physical properties, and applications.^[^ ^172^ ^]^ Tremel and co‐workers have focused on biomedical applications of inorganic Janus particles.^[^ ^173^ ^]^ Faivre and colleagues discussed, apart from the synthesis of Janus composites and the resulting types of materials, their applications with emphasis on drug delivery.^[^ ^174^ ^]^ Additional reviews on the synthesis, properties and applications of Janus composites have been published by several other research teams.^[^ ^175^, ^176^, ^177^, ^178^ ^]^

Thus from the above, we notice that various kinds of Janus particles can be produced, as metal–metal, metal–dielectric, metal–polymeric, and non‐metallic nanocomposites, with either spherical or anisotropic shape. Seed‐mediated and one‐pot methods are available for their production, which sometimes leads to the generation of particularly attractive materials which can combine, for example, magnetic and plasmonic properties.

**Figure 8:**
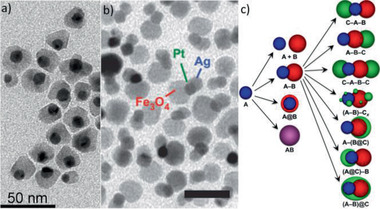
a) TEM image of Au‐Fe_3_O_4_ heterostructured nanocrystals, b) Ag‐Pt‐Fe_3_O_4_ heterotrimers, c) different A, B heterodimer and A, B, C heterotrimer configurations that are possible by the use of sequential seeded growth reactions. a) Reproduced with permission.^[^ ^185^ ^]^ Copyright 2017, American Chemical Society. b) Reproduced with permission.^[^ ^188^ ^]^ Copyright 2012, Springer Nature. c) Reproduced with permission.^[^ ^187^ ^]^ Copyright 2017, American Chemical Society.

One of the most important types of heterostructure composites are the heterodimers, or simply dimers. Sheng and Xue prepared Au‐Fe_3_O_4_ dimers by using the thermal decomposition method in the presence of 1,2‐hexadecanediol (HDOL), which played a crucial role in their successful formation. Fe(CO)_5_ was the iron source whereas HAuCl_4_ was the gold precursor. The “hot‐injection” of the iron precursor in the solution containing solvent and surfactants was followed by the quick addition of the gold precursor.^[^ ^179^ ^]^ In another work, the optical and magnetic properties between core–shell structure, dumbbell‐like dimers, and chemical cross‐linked pairs in the Au‐Fe_3_O_4_ system were compared. The capabilities of this magneto‐plasmonic composite in those different configurations were demonstrated, and the role of the magnetic and plasmonic components in the resulting properties was discussed.^[^ ^180^ ^]^ Guardia et al. reported the synthesis of Au‐Fe_x_O_y_ dimers through two synthesis routes, a one‐pot and a two‐pot method. These authors found that the absence of HDOL and the addition of chloride ions enabled to produce dimers in a controlled size range. Exceptional values of specific absorption rates (SARs) in magnetic hyperthermia were recorded for these nanocomposites.^[^ ^181^ ^]^ Exchange bias has been observed in 8 nm Au‐9 nm Fe_3_O_4_ nanocomposites prepared by Srikanth and co‐workers. The generation of exchange bias was assigned to the existence of highly disordered surface spins which foster due to the presence of stress (order of a few GPa) across the Au‐Fe_3_O_4_ interface. The exchange bias field could be modified by changing the size of both Au and magnetite domains.^[^ ^182^ ^]^ In a different approach, Dewi et al. synthesized Au‐Fe_3_O_4_ dimer nanocomposites by first producing each component in separate synthetic steps. After proper functionalization of both parts, amine bond formation was used to dimerize the two particle types.^[^ ^183^ ^]^ Pariti et al. produced Au‐Fe_3_O_4_ bifunctional nanocomposites through a single step hot‐injection precipitation method. Iron pentacarbonyl was used as Fe precursor, while chloroauric acid was the Au source. The synthesis was carried out in the presence of OAc and OAm. Cell viability and toxicity studies were performed for these composites, after their biofunctionalization with cysteine, showing that they were not toxic to CHO cells even at moderately high exposure.^[^ ^184^ ^]^ The Puntes group performed a systematic study on the seeded growth synthesis of Au‐Fe_3_O_4_ heterostructures (**Figure** **8**a), providing insights on the role of the iron precursor used (Fe(CO)_5_ versus Fe(acac)_3_). The role of other parameters such as surfactants, Fe/Au ratio, temperature, and solvent type was also investigated, in the presence of 10 nm pre‐synthesized Au seeds.^[^ ^185^ ^]^ Murray and co‐workers reported the synthesis of Janus heterodimers via asymmetric functionalization of Fe_3_O_4_‐Pt and Fe_3_O_4_‐Au dimers through sequential ligand exchange stages using dendritic ligands bearing different surface binding groups. The hydrophobic and hydrophilic ligands that contain phosphonic acid and disulfide surface binding moieties selectively coated the Fe‐oxide and the Pt (or Au) parts of the dimer, respectively.^[^ ^186^ ^]^

Schaak and co‐workers have published some robust work on the seeded growth on dimer and trimer nanocomposites (Figure 8c), such as Pt‐Fe_3_O_4_, Au‐Pt‐Fe_3_O_4_, and Ag‐Pt‐Fe_3_O_4_, rationalizing the key reaction parameters that need to be carefully controlled to minimize undesired nanomaterial byproducts.^[^ ^9^, ^187^ ^]^ These researchers managed to develop a total synthesis framework for the production also of M‐Pt‐Fe_3_O_4_ (M = Au, Ag, Ni, Pd) heterotrimers (Figure 8b) and heterotetramer composites such as M_x_S‐Au‐Pt‐Fe_3_O_4_ (M = Pb, Cu). Also, higher‐order oligomers based on the heterotrimeric Au‐Pt‐Fe_3_O_4_ building block were prepared.^[^ ^188^ ^]^ Sharma et al. produced Ag‐CoFe_2_O_4_ dimer nanocomposites through a two‐step solution‐phase route. XRD measurements showed a shift to lower 2*θ* values in respect to the “expected” peaks for the CoFe_2_O_4_, indicating the expansion of its lattice structure, whereas no shift was recorded for the position of the Ag peaks, which indicated its intact lattice structure. It was found that the silver offers a thermal stabilization for the dimer composites, which is better in comparison to CoFe_2_O_4_ alone, thanks to its interface with cobalt ferrite particles. The interface effect resulted in a strongly enhanced magnetic anisotropy and a large coercivity at 2 K for the dimers. These magneto‐optical composites are considered as potential nanovectors for drug delivery.^[^ ^189^ ^]^

The same group reported also the synthesis of Ag‐Fe_3_O_4_ nanodimers.^[^ ^190^ ^]^ In another report, a one‐pot colloidal protocol was used to synthesize magneto‐optical Ag_2_S‐Fe_7_S_8_ heterodimers through the transformation of ternary AgFeS_2_, which took place by internal reaction at elevated temperature in OAm.^[^ ^191^ ^]^ Ni_3_S_4_ nanorods were prepared through a hot‐injection method and further used as matrices to produce 1D Ni_3_S_4_‐PtCo heteronanorods by adding Pt(acac)_2_, cobalt acetate tetrahydrate, and surfactants at high temperature in organic solvents. These composites displayed high catalytic performance on the reduction of I_3_^–^ when used as counter electrode catalysts in dye‐sensitized solar cells.^[^ ^192^ ^]^ A simple two‐step solvothermal approach was used to fabricate Janus heterostructures and heterodimers, completely composed of binary transition metal oxides. The final Mn_3_O_4_‐TiO_2_/ZnO/Fe_3_O_4_ Janus‐shaped nanocomposites are very promising for use in theranostic fields.^[^ ^193^ ^]^ Asymmetric Fe‐Mn oxide hybrid nanocomposites were obtained using a seed‐mediated thermal decomposition‐based synthesis. 1‐Octadecene and benzyl ether were tested as solvents. It was evidenced that the choice of the latter solvent enabled the orientational growth of Mn_1‐x_O onto iron oxide nanocubes yielding mainly dimers and trimers while 1‐octadecene led to large NPs. The reason for such different behavior was suggested, based on the different polarity between the two solvents. Advanced electron microscopy techniques (HRTEM and HAADF‐STEM tomography) were used to shed light on the morphology and the crystallographic features of the composites.^[^ ^194^ ^]^ A review paper on the properties and applications of self‐assembled dimers was published by Xu et al.^[^ ^195^ ^]^

Therefore, one can observe that dimer and oligomer heterostructured magnetic nanocomposites have been increasingly formed through seed‐mediated routes. The resulting heterostructures combine two sets of properties (e.g., magnetic and plasmonic), and, unlike the core–shell composites, the surfaces of both components are widely accessible (there is no “protected core”) which is beneficial for certain applications. Interface effects are also of interest for certain properties (e.g., magnetic anisotropy‐coercivity). Still, less common preparation methods may be also efficient for the preparation of such composites: one example consists in amine bond formation, after suitable surface functionalization of the two components which will dimerize to form the nanocomposite.

## 3 Inorganic Magnetic Nanoparticle–Organic/Organometallic Composites

For nanoparticle systems, especially those used for biological applications, it is common to coat the inorganic magnetic nanoparticle with an organic component. The simplest form of this is used to form colloidal nanoparticle dispersions, where a surfactant or ligand binds to the magnetic metal or metal oxides surface, and a tail group protrudes into the solvent where it interacts with the solvent and makes the particles dispersible. This configuration of NPs is one of the most common, where chemists use colloidal synthesis techniques to grow NPs in solution with ligands helping to guide the growth process and control a particle final size and shape. Particles can be grown in either hydrophobic or hydrophilic media, with hydrophobic conditions being common to achieve tunable particles sizes and controlled shapes utilizing organic solvents and ligands. The particles can have their ligands exchanged between hydrophobic and hydrophilic compounds to disperse the particles in a range of solvents. For the particles synthesized under hydrophobic conditions, the ligands/surfactants that need to be exchanged are long chain molecules including amines, acids, diols, and phosphines with some typical examples used to synthesize magnetic nanoparticles being oleylamine,^[^ ^196^ ^]^ OAc,^[^ ^197^ ^]^ 1,2‐hexadecanediol,^[^ ^198^ ^]^ and trioctylphosphine.^[^ ^199^, ^200^ ^]^

To utilize these nanoparticle systems to create composites with a functional organic component, organic surface chemistry can be employed to impart a range of properties on the final particles. Often the surfactants used in the initial synthesis conditions will cause limitations or complications to be overcome for the future control and modification of the organic surface chemistry of the composites. This is particularly true in biological applications, where the initial synthesis conditions can limit the particles from being used in the intended application due to concerns about residual chemicals being present in the final product as a result of the synthetic chemistry.

### 3.1 Organic Surface Chemistry

Organic surface chemistry is the most common way to modify nanoparticles to form a nanoconjugate material. Organic compounds can engulf or bond to the surface of the magnetic ΝPs allowing them to be readily dispersed in aqueous or organic solvents. The surface chemistry of the particles can be designed particularly for the application in question. The ligand shells formed when synthesized in organic solvents and typically in high‐temperature synthetic routes are usually hydrophobic. To form a hydrophilic ligand shell there are several ways, one is to have the shell formed during synthesis, but equally as common is to take particles that are formed with a ligand shell that makes them hydrophobic and then to add or replace the functionality to render them hydrophilic and biocompatible.^[^ ^201^ ^]^ One of the core reasons for doing this, is that the initial organic shell might be needed for the synthesis of the particles to better control their size and shape, and thus post‐processing steps are often required.

The organic functionality that is added to the surface is typically used to make the nanoparticles hydrophilic, and to endow them biocompatibility. For hydrophilic ligands, an extensive array of functionalities has been explored for increasing the particles biological compatibility and functionality.^[^ ^201^ ^]^ One particular type are polymer shells that are commonly used to tune particles stability in water and under biological conditions. The polymer shells, for example, with polyethyleneglycol (PEG), allow the stability of the particles to be tuned in the blood stream,^[^ ^197^, ^202^ ^]^ increase the uptake of NPs by cells,^[^ ^203^ ^]^ and also tune the modes of relaxation that are used for magnetic sensing.^[^ ^204^ ^]^ Another effect is that the organic ligands have been shown to increase the particles stability against oxidation, for example with using dimercaptosuccinic acid (DMSA).^[^ ^205^ ^]^

There are five routes to add the organic functionality to the surface of the nanoparticles. The first and simplest way from a surface chemistry approach is to synthesize the nanoparticles with the needed functionality present. This occurs by the organic functionality at the end of the ligand or polymer binding to the surface of the growing nuclei and has been reported to occur shortly after the nucleation of the nanoparticulate material.^[^ ^206^, ^207^ ^]^ Although the process seems simple, strongly binding ligands can inhibit NP growth, while weakly binding ligands can lead to uncontrolled growth conditions and aggregation.^[^ ^199^ ^]^

Second, the functionality can be grafted to the nanoparticles after they have been synthesized “bare”.^[^ ^208^, ^209^ ^]^ This method can only be used in particular cases where NPs are synthesized without surfactants and are stabilized by ions in solution and through the aid of the charge of the particles, such as in the case of the co‐precipitation method. The advantage of this method is that the functionality can be added after the synthesis and thus will not interfere with the synthesis of the NPs. The functionality will need to have a specific functional group to interact with the nanoparticles present and the ligand cannot bind too strongly to the magnetic NPs as this might cause the material to re‐dissolve. The method of using grafting typically leads to some aggregation occurring before stabilizing ligands are added, and often involves cleaning steps to remove excess salts before the ligands are attached.^[^ ^201^ ^]^ In the case of iron oxide, hydroxyl groups are present in the surface when synthesized bare via co‐precipitation and these are amphoteric allowing them to react with acids and bases.^[^ ^210^ ^]^ The ligands attached will usually be strongly binding, utilizing the amine, acid, 1,2‐diol (catechol), phosphonate, and thiol functionalities with the catechol and phosphonate functionalities being preferred for their strong affinity for the metal oxide surface.^[^ ^211^ ^]^ An advantage of this method is that the ligand with the needed functionality can be attached to the surface of the nanoparticle after the nanoparticle has been synthesized, allowing the use of strongly binding moieties that might hinder NP growth. An example is the catechol functionality in modified dopamine to be used.^[^ ^212^ ^]^

Third, to replace the ligand that is natively covering the as‐synthesized NPs is carried out via a method called ligand exchange.^[^ ^213^ ^]^ This method relies on the molecule used for the exchange to reach the surface of the particle and to have a strong binding moiety on the surface to displace the current ligand shell. One example of using ligand exchange with designed ligands is dopamine‐modified ligands to utilize the strong binding catechol groups to the surface of the metal or metal oxide. For example, dopamine was conjugated onto poly(isobutylene‐alt‐maleic anhydride) (PIMA) and used for ligand exchange to replace OAc^[^ ^214^ ^]^ and OAm.^[^ ^31^ ^]^ The PIMA backbone could then be further functionalized with amine‐modified PEG.^[^ ^214^ ^]^

The ligand exchange approach can be a challenging technique for a variety of reasons, first getting complete exchange of the surface ligand can be difficult to achieve and to monitor.^[^ ^215^, ^216^ ^]^ In the case of using ligand exchange to NPs for biological applications, the residual ligands could be toxic and will limit their ability to be exploited.^[^ ^217^ ^]^ For example, Davis et al. studied the ligand exchange of OAc on iron oxide NP surfaces with C14 labeling of the OAc. They found that the catechol functionality replaced the OAc the most efficiently, but even this functionality did not fully displace the carboxylic acid completely from the particles surface.^[^ ^216^ ^]^ On top of this, the exchange of the ligand can often lead to oxidation or dissolution of the NP surface during the exchange process.^[^ ^31^, ^218^ ^]^ Palma et al. observed a reduction of the saturation magnetization and the core size with the exchange of OAc with DMSA.^[^ ^218^ ^]^ For magnetic NPs the typical surface functionalities used to strongly anchor surfactants to the surface of them are phosphonate and catechol based systems.^[^ ^31^, ^216^, ^220^ ^]^ This is due to their strong affinity for metal oxide surfaces,^[^ ^219^, ^220^ ^]^ which is either the surface of the synthesized NPs or occurs naturally at the surface post synthesis.^[^ ^31^, ^221^ ^]^

The fourth method is to engulf the particles by encapsulating them within a functionality provided often from amphiphilic polymers^[^ ^222^, ^223^ ^]^ co‐block polymer micelles,^[^ ^224^, ^225^ ^]^ or vesicles.^[^ ^223^ ^]^ The method of using ligand exchange or engulfing the particle is important for any type of NP system where certain surfactant or ligand species are needed in the NP synthesis. However, if the NPs are not synthesized bare, and the initial ligand shell is not replaced completely, it will be present in the final product: this means that if the native shell of the NPs is toxic the system will always have possible toxic elements present. The ability to engulf the nanoparticles can also be used to assemble several NPs simultaneously^[^ ^225^ ^]^ to form cluster‐type arrangements of the host NPs, thus aiming to tune the magnetic response of the collective clusters.^[^ ^226^, ^227^, ^228^, ^229^ ^]^ These assemblies have been shown to enhance the heating properties of magnetic nanoparticles, and increase their contrast in MRI applications.^[^ ^225^, ^230^, ^231^, ^232^ ^]^ Another advantage of the engulfing procedure is that other molecules can be incorporated within the micellar organic coating, such as drug molecules for targeted drug delivery.^[^ ^233^ ^]^ A typical methodology to engulf the magnetic NPs is to create a micelle structure that will incorporate one or more particles inside it. Such examples use amphiphilic block copolypeptides assembled around the clusters^[^ ^225^ ^]^ with a recent example forming nanovesicles from poly(ethylene oxide)‐block‐poly(e‐caprolactone) polymer.^[^ ^234^ ^]^ This method includes magnetic NPs synthesized with organic ligand shells, typically with OAc, and then coated with an amphiphilic polymer shell in which the long carbon chains intercalate between the OAc chains, allowing the polymer to coat it and confer stability.^[^ ^222^, ^223^, ^235^ ^]^ This method is not sensitive to the surface chemistry of the NP material and can be used on a wide variety of nanomaterials that have organic ligands attached to their surface.^[^ ^222^ ^]^

Finally, an initial ligand shell can be used as a platform for further synthetic reactions to build the final ligand shell needed. To carry this out, a ligand that can be further modified must be formed during the NP synthesis or attached to the NPs post‐synthesis by one of the strategies discussed above. This method is commonly used in combination with the other four, as an initial reactive molecular group must be attached to the surface. It is common to couple this strategy with ligand exchange replacing molecules such as OAc with molecules that can be further modified. This has been used for attaching polymer ligands to magnetic NPs where an initial ligand is attached to the nanoparticle surface that acts as an initiator, and then the polymer is grown off the ligand on the NPs surface.^[^ ^236^, ^237^, ^238^, ^239^ ^]^ Another type of chemistry that has been utilized to carry out covalent bonding of an incoming ligand with an initial ligand shell is carbodiimide chemistry, which occurs by coupling an acid functionality with an amine forming an amide bond.^[^ ^238^, ^239^ ^]^

**Figure 9:**
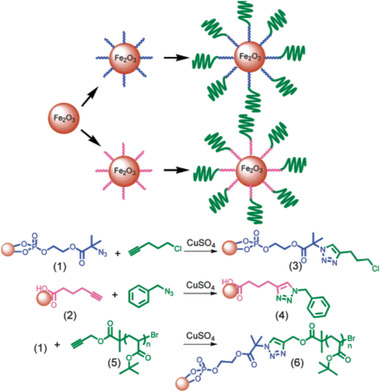
A scheme of using click chemistry to functionalize the surface of Fe_2_O_3_ NPs. The first step is either attaching to the surface a phosphonate (in blue) or carboxylic acid (in pink) functionalized ligand with an azide or alkyne on the other end of the molecule. This is then reacted with the additional functionality over a Cu^2+^ catalyst. Reproduced with permission.^[^ ^243^ ^]^ Copyright 2006, American Chemical Society.

The further modification can take place via click chemistry: this highly efficient method occurs under mild conditions which will not adversely affect the magnetic NPs. A common form of this is the copper (I) catalyzed azide‐alkyne cycloaddition “click” chemistry.^[^ ^240^, ^241^, ^242^ ^]^ An example of this reaction type was reported by White et al. where they used ligand exchange to replace OAc with a range of small molecules having a phosphonic acid or a carboxylic acid group at one end to anchor to the Fe‐oxide NPs and an azide or alkyne at the other end for further functionalization.^[^ ^243^ ^]^ The particles were then functionalized through the click chemistry with a polymer that had been modified to have an alkyne or azide functional group for the reaction, as presented in **Figure** **9**.^[^ ^243^ ^]^ The use of click chemistry needs the presence of the azide and alkyne functionalities to progress.

### 3.2 Biological Molecules

Magnetic nanoparticles conjugated with DNA, antibodies, and oligonucleotides can be employed for separation techniques and detection of biomarkers.^[^ ^244^ ^]^ The magnetic NPs can be directly functionalized with the biological molecules if they are readily synthesized with hydrophilic ligands on their surface and have amine or carboxylic acid groups available for further functionalization.^[^ ^244^ ^]^ Much like in the discussions above, the biological molecules can be attached chemically through conjugation to form a covalent bond with the functional groups on the particles surface, or through electrostatic adsorption to the particle. Electrostatic binding can be also used, for example, polyethleneimine‐encapsulated NPs have been applied for gene delivery by adsorbing DNA onto the particles.^[^ ^245^ ^]^ Similar to the above discussion, direct ligand exchange of OAc with biological materials on the surface of the iron oxide NPs can be carried out: in one specific example, DNA was bonded to a polymer (polyacrylic acid) and then directly grafted to the surface of the iron oxide NPs.^[^ ^246^ ^]^ A ligand exchange approach involved a Janus DNA tetrahedron structure that had three carboxyl groups modified to the DNA strands on one end and an aptamer sequence containing DNA on the other: this complex could bind firmly to the iron oxide NPs displacing the OAc shell which was present from the synthesis.^[^ ^247^ ^]^ DNA was also conjugated to dextran‐coated aminated magnetite NPs using sulfosuccinimidyl 4‐(*N*‐maleimidomethyl)cyclohexane‐1‐carboxylate as a cross linker.^[^ ^248^ ^]^

Other chemical strategies have been utilizing click chemistry to bond the biological molecules to the NPs shell: this was performed by attaching DNA via a copper catalyzed azide‐alkyne reaction.^[^ ^249^ ^]^ The iron oxide NPs were first functionalized with azide, and then reacted with the alkyne modified oligonucleotide in the presence of the Cu(I) catalyst.^[^ ^249^ ^]^ Click chemistry has been utilized to attach a range of biologically active molecules and proteins.^[^ ^243^ ^]^

Other nanoconjugates include incorporation of NPs prepared in hydrophilic^[^ ^250^, ^251^ ^]^ or hydrophobic conditions^[^ ^252^ ^]^ in liposomes. This strategy has been used for protecting and delivering NPs into the blood stream.^[^ ^251^ ^]^ Liposomes have been particularly attractive for magnetic NPs as they can be filled with the particles and also additional drug molecules,^[^ ^253^, ^254^ ^]^ which can then be paired with magnetic hyperthermia. The heating properties of the magnetic NPs can be exploited to locally heat the membrane of the liposome for triggered drug release.^[^ ^250^, ^254^, ^255^, ^256^ ^]^

### 3.3 Organic Surface Chemistry on Magnetic Nanoparticle/Inorganic Composite

The surface chemistry of the common composites is extremely important for tailoring the nanoparticles properties. Silica shells are commonly used as a platform for attaching the surface chemistry through direct functionalization with organic or biological molecules by reacting them with the silanol groups on the SiO_2_ shells surface.^[^ ^257^, ^258^ ^]^ This has been successfully carried out for electrostatic DNA separation with amino functionalized silica.^[^ ^259^ ^]^ The silica shell also provides protection to the magnetic core during these synthetic modifications to provide the organic functionality.

**Figure 10:**
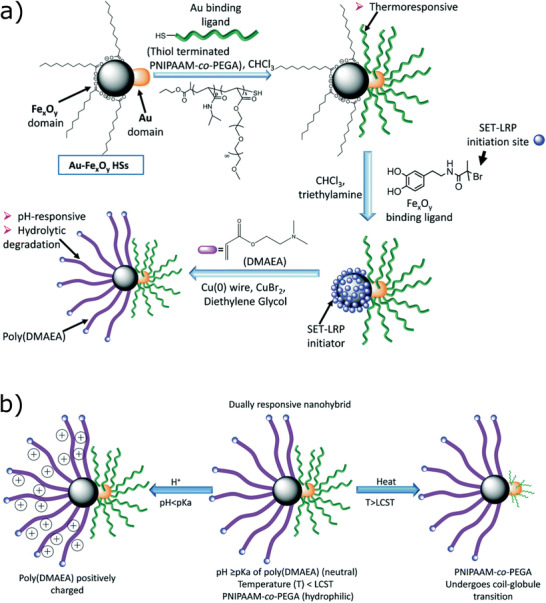
Schematic of the surface functionalization of the Au/Fe_x_O_y_ Janus particle with thiol and catechol functionalized ligands, leading to the formation of the dual responsive nanohybrid. Reproduced with permission.^[^ ^264^ ^]^ Copyright 2018, Royal Society of Chemistry.

Noble metals have a strong binding affinity for thiol functionalities, unlike the corresponding behavior of typical magnetic materials. This differing surface chemistry can be used to attach chemical functionality or biofunctionality that are common to gold chemistry to the noble metal shell or component, such as binding to proteins and DNA by utilizing the affinity of gold to thiol functional groups.^[^ ^260^, ^261^, ^262^ ^]^ The difference in chemical affinity between noble metal and magnetic NPs has been particularly exploited in the case of noble metal/magnetic Janus particles. Au‐Fe_3_O_4_ Janus particles have been used to form amphiphilic NPs, with one part having a hydrophobic functionality attached to the Au while the Fe_3_O_4_ has a hydrophilic one.^[^ ^263^ ^]^ Janus particles of Au‐Fe_3_O_4_ can be exploited by separately modifying the gold and the iron oxide with different bioactive molecules such as drug molecules on one side and targeting compounds on the other.^[^ ^263^ ^]^ A recent example of utilizing the surface chemistry of a Janus particle was reported by Kakwere et al. with a gold/iron oxide Janus nanoparticle where the gold and iron oxide were functionalized separately using thiol functionalized polymers for the gold and catechol groups for the iron oxide to separately coat each part of the Janus particle with a distinct polymer functional group (**Figure** **10**.)^[^ ^264^ ^]^

### 3.4 Magnetic Nanoparticle–Organometallic Composites

Organometallic compounds can also be attached onto magnetic NPs providing an additional functionality through bonding the organometallic species or an inorganic metal center to the NPs surface or onto the ligand shell surrounding the material. One such case is binding the drug cisplatin onto magnetic NP composites to help for its drug delivery. This has been achieved through several strategies^[^ ^263^, ^265^, ^266^, ^267^ ^]^ one of which is exploiting the surface chemistry of gold in an Au‐Fe_3_O_4_ nanoparticle and binding the cisplatin to the thiol‐containing ligand bound to the gold.^[^ ^263^ ^]^

Another common reason to attach metallic ions to NPs is for labeling. For instance, radionucleotides can be bound to the surface of magnetic NPs to use in multimodal imaging (MRI and radionucleotide imaging). Examples of this case include using ^64^Cu,^[^ ^266^ ^] 124^I,^[^ ^267^ ^]^ and ^99m^Tc^[^ ^268^ ^]^ complexed to magnetic NPs. These composites are all synthesized by conjugating the radionucleotide to the ligands surrounding the magnetic NPs. For example ^99m^Tc was complexed to the phosphate ligand shell around iron oxide NPs by Gao et al. for tumor imaging.^[^ ^268^ ^]^ For use as multi‐modal MRI contrast agents, Gd‐based chelates used for T1 MRI can be bound to magnetic NPs, in particular iron oxide, which acts as a T2 MRI contrast agent.^[^ ^269^ ^]^

**Figure 11:**
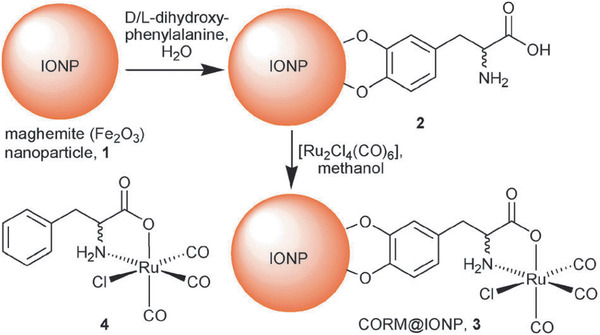
Schematic illustration of the binding of the ruthenium carbonyl to the ligand shell of an iron oxide particle. Reproduced with permission.^[^ ^270^ ^]^ Copyright 2013, Royal Society of Chemistry.

The binding of metal carbonyl onto magnetic NPs has been explored as a way to cause heat‐induced CO release under biological conditions: a relevant work presents the binding Mn, Ru, and Fe compounds with bound carbonyl groups to the surface of iron oxide NPs.^[^ ^270^ ^]^ Such binding has also been used to acquire optical bio‐probes due to their intense absorption and stretching bands observable in infrared spectroscopy.^[^ ^271^ ^]^ The strategies to bind the carbonyl complexes to the NPs are similar to what has been stated above: once the organic capping ligand has been added, the ligand is further functionalized to bind to the metal carbonyl complex. Kunz et al. first bound D/L‐3‐(3,4‐dihydrophenyl)alanine to the surface of the iron oxide NPs through its catechol surface moiety, and further reacted amine and carbonyl groups at the other end of the molecule to the metal carbonyl compound (**Figure** **11**).^[^ ^270^ ^]^

### 3.5 Magnetic Nanoparticle–Organic Framework Composites

Composites of metal‐organic frameworks (MOFs) and covalent organic frameworks (COFs) have been drawing increasing attention aiming to access the unique properties of these frameworks, such as their high surface areas and tailorable pore sizes. MOFs have been the most widely explored to form composites with magnetic NPs^[^ ^272^ ^]^ and have been destined for a range of applications such as drug delivery,^[^ ^273^ ^]^ catalysis,^[^ ^274^ ^]^ and fluorescent probes.^[^ ^275^ ^]^ The magnetic functionality is typically used for the magnetic separation of the MOFs from solution when used as catalysts,^[^ ^274^ ^]^ probes,^[^ ^275^ ^]^ or to locally heat via magnetic hyperthermia.^[^ ^274^ ^]^ A typical synthetic strategy for synthesizing an MOF‐magnetic nanoparticle hybrid would be to grow the MOF in the presence of the NPs and have the magnetic nanoparticles become incorporated into the structure during growth.^[^ ^273^, ^276^, ^277^ ^]^ This can be carried out ubiquitously as the synthetic conditions needed for the formation of the MOF are usually relatively mild.^[^ ^278^ ^]^ Another similar strategy is embedding the magnetic NPs within a ceramic compound that is the precursor for a MOF, and then grow the MOF from the compound already containing the magnetic NPs.^[^ ^278^ ^]^ Also, the MOF can be grown directly on the surface of the magnetic particle creating a shell of the MOF with a magnetic core: this can be achieved by first functionalizing the magnetic particle with mercaptoacetic acid and then carrying out the assembly of the MOF which directs to its growth as a shell around the particles. First the metal ions for the MOF (Cu or Fe) were bound to the carboxylic group on the iron oxides surface, and then this was further reacted with the benzenetricarboxylic acid units to form the MOF.^[^ ^279^ ^]^

Recent work on magnetic nanoparticle‐COF composites has been developed for synthesizing COFs for adsorption and removal of pollutants in water and food,^[^ ^280^, ^281^ ^]^ purification of hydrocarbon fuels^[^ ^282^ ^]^ and catalysis,^[^ ^283^ ^]^ and then using the magnetic component to allow the separation of these materials from solution. For COFs, the typical approach taking place is the encapsulation method of the NPs which is typically performed chemically: A first step involves functionalizing the nanoparticles surface either with an organic linker,^[^ ^284^, ^285^, ^286^ ^]^ or with silica^[^ ^283^ ^]^ and then the organic framework is grown on top of the particles. One such method was implemented by synthesizing dopamine‐capped iron oxide NPs, which afterward reacted with a COF building block triformylphloroglucinol (Tp).^[^ ^287^ ^]^ The Tp‐functionalized NPs were then used in the reaction to synthesize the COF from the building blocks of the Tp and *o*‐tolidine to achieve the COF engulfing the iron oxide NPs.^[^ ^287^ ^]^

### 3.6 Future Outlooks

All of the myriad different functional composites that can be generated from an inorganic nanoparticle and an organic‐based compound are synthetically related through a plethora of ways to create the first linkages between the inorganic compound and the ligand/surfactant shell. In this way, all of the further modifications and functionality are built upon this initial step. As the complexity and understanding of controlling the magnetic nanoparticle as well as controlling the surface chemistry expands, the main difficulties come from the use of the particles. Several limitations on this type of research are common and a unified approach for studying the organic materials bound to magnetic materials has not been created yet: Typical approaches via nuclear magnetic resonance cannot be used due to the broadening that the magnetic component causes. This prevents a thorough understanding of every material type discussed in this section and the research going forward as control over the organic surface chemistry is needed in all of these synthetic systems to reach maturity. Because of this, the field is less mature than it is with systems like gold nanoparticles.

To use the materials in biomedical applications, a high level of purity must be obtained. Several of the most practical reaction systems for a nanoparticle chemist, especially in forming magnetic nanoparticle/inorganic composites used as a platform for the organic components, will be carried out with toxic chemicals. These need to be removed with a high degree of efficacy before they could be used biomedically but this can be quite complex considering the above‐mentioned analytical difficulties. Another approach will be to develop new synthesis routes to achieve the production of the controlled magnetic nanoparticle composites while excluding any toxic components from the start. Green synthetic approaches are already being explored, but they do not yet achieve the same level of control as the more developed organic medium‐based high‐temperature reactions.

## 4 Applications of Magnetic Nanocomposites

### 4.1 Magnetically Separable Catalysis

One of the most important applications of magnetic nanoparticles is the catalysis of a variety of reactions. The so‐called synergistic effects, often occurring due to the presence of an interface between the different components of the composites, help to improve the overall properties of the composite. In this way, the resulting properties are not only a “sum” of the properties of the individual components, but in addition enhanced or new properties may also appear. Kokate et al. prepared a magnetite‐silica‐gold nanocomposite through a single‐step one‐pot green synthesis at room temperature. The composites were employed as magnetically separable and reusable catalysts in a solvent‐free oxidation of benzyl alcohol.^[^ ^288^ ^]^ The porous silica matrix stabilized the magnetite and the gold NPs but it also had a high surface area, making the composite accessible for the reactants. In another report, well‐dispersed Ag NPs were loaded on the surface of Fe_3_O_4_‐modified RGO via a two‐step approach. Through the reduction of Ag^+^ ions, highly dispersed Ag NPs were in situ formed on the RGO/Fe_3_O_4_ substrate. The reaction solvent was found to have a great effect on the resulting composites. The final composite was applied for the typical 4‐NP to 4‐AP catalytic reaction.^[^ ^289^ ^]^ A simple, facile and quick sonochemical method was developed to produce Fe_3_O_4_/Ag composite by three‐step reactions. Before the Ag^+^ reduction, the surface of the magnetite NPs was functionalized with APTES. The products showed good catalytic activity in the degradation of RhB.^[^ ^290^ ^]^ Yuan and co‐workers prepared titania‐coated carbon encapsulated magnetite composites through a three‐step approach. An enhanced photocatalytic activity was evidenced for the composite, when applied to degrade model contaminated water (a phenol aqueous solution). The increased hydroxyl groups of the composite probably inhibited the recombination of electron–hole pairs.^[^ ^291^ ^]^

An in situ co‐precipitation and reduction method was used to prepare Fe_3_O_4_/RGO composites. Compared to active carbon, this composite showed 3.7 times higher adsorption capacity for RhB and 30 times faster adsorption rates. A surface area of 296.2 m^2^ g^−1^ was evidenced.^[^ ^292^ ^]^ Dabiri et al. prepared Fe_3_O_4_@RGO@Au@C composites which demonstrated excellent catalytic performance for the reduction of nitrophenols and the Suzuki–Miyaura cross‐coupling reaction.^[^ ^293^ ^]^ Barberry fruit extract was employed as a reducing and stabilizing agent for the environmentally friendly in situ synthesis of Cu NPs supported on a RGO‐Fe_3_O_4_ nanocomposite. The final Cu/RGO‐Fe_3_O_4_ material displayed high catalytic activity for the *O*‐arylation of phenols using aryl halides.^[^ ^294^ ^]^ Another paper describes the synthesis of a magnetic inorganic–organic catalyst, PTA@Fe_3_O_4_/ethylenediamine‐MIL‐101 composite. This material was used as a catalyst for the one‐pot synthesis of diverse 2H‐indazolo[2,1‐b]phthalazine‐triones in good yield under solvent‐free conditions. The MIL‐101(Cr) is a promising MOF with high surface area, good thermal and chemical stability, large mesopore channels, and wide pentagonal and hexagonal microporous windows which facilitate fast in‐pore transport of substrate and products in catalysis.^[^ ^295^ ^]^ A template‐induced assembly mechanism was used to prepare Fe_3_O_4_‐PDA hybrid hollow microsphere composites. The composites exhibited intrinsic peroxidase‐like activity, as they could rapidly catalyze the oxidation of typical substrates 3,3′,5,5′‐tetramethylbenzidine (TMB) in the presence of hydrogen peroxide. Apart from their excellent catalytic performance, magnetic sensitivity, reusability, and stability were also demonstrated for these composites.^[^ ^296^ ^]^

Zhang et al. prepared magnetic mesoporous silica composites by embedding magnetic oxide NPs into abundance of host silica matrix. The composites possessed ordered hexagonal mesopores, very high surface area (larger than 1000 m^2^ g^−1^) and large pore volume. These materials were used as recyclable heterogeneous catalysts for efficient catalytic aldol reaction after their aminoalkylsilylation.^[^ ^297^ ^]^ Fe_3_O_4_/SiO_2_ (aerogel and MSU‐X) composites with very low Fe_3_O_4_ content were produced by co‐condensation of MPTES‐functionalized magnetite NPs with a silicon alkoxide (MPTES: (3‐mercaptopropyl)trimethoxysilane). The nanocomposites were used for the oxidation reaction of TMB with H_2_O_2_. The Fe_3_O_4_ NPs were incorporated onto the silica and they were freely accessible to the reactants.^[^ ^298^ ^]^ An ultrasound‐assisted wet impregnation method was employed to prepare Fe_3_O_4_‐TiO_2_@MWCNT composites. These materials were used to degrade 2‐chlorophenol. The catalysts presented combined advantages due to the co‐existence of MWCNTs and Fe_3_O_4_.^[^ ^299^ ^]^ A Fe_3_O_4_‐SiO_2_‐Pd nanocomposite was used to catalyze Suzuki–Miyaura coupling reactions of aryl halides (I, Br, Cl) with arylboronic acids in water under mild conditions and low Pd loading. The stabilization of Pd onto the surface of the magnetite NPs was achieved through their functionalization with phosphinite in the presence of an imidazolium ionic liquid moiety.^[^ ^300^ ^]^ A maghemite/montmorillonite composite was prepared by a co‐precipitation and calcination method. The calcined composite retained their magnetism thanks to the fact that montmorillonite inhibited the growth of *γ*‐Fe_2_O_3_ NPs, as well as their phase transition. Calcination helped also to strengthen the catalytic activities of the composite toward phenol degradation, since it boosted the interaction between iron oxides and aluminosilicate framework, resulting in more negatively charged surface. High stability and low Fe leaching for the catalyst was shown.^[^ ^301^ ^]^

A facile one‐step hydrothermal method was used to fabricate magnetically separable P25/CoFe_2_O_4_/graphene photocatalyst with differing P25 contents. GO was mainly reduced and decorated with well‐dispersed TiO_2_ and CoFe_2_O_4_ NPs. The composites catalyzed the photodegradation of MB, methyl orange (MO), and neutral dark yellow dyes. Synergistic effects among their individual components helped for a greatly improved catalytic performance.^[^ ^302^ ^]^ CuFe_2_O_4_‐RGO nanocomposites were produced via an in situ co‐precipitation reduction‐based synthetic approach. Synergistic effects, coming from the combination of the unique properties of each one of the two components were exploited for the catalytic reduction of 4‐NP to 4‐AP and the epoxidation of styrene to styrene oxide. The catalytic activity of the composites was much better than that of sole CuFe_2_O_4_.^[^ ^303^ ^]^ Cu‐Fe_3_O_4_@graphene composites produced through a one‐step solvent‐thermal method were employed to reduce 4‐NP to 4‐AP. The Cu NPs and Fe_3_O_4_ NPs were densely and evenly deposited on the graphene sheets. Low dosage was sufficient for high catalytic activity in these stable catalysts.^[^ ^304^ ^]^ NiFe_2_O_4_@GO‐Pd nanocomposites were produced through a facile one‐pot hydrothermal strategy. These materials were used in a Pd‐catalyzed Heck reaction in an ethanol–water system as a green solvent. A better catalytic activity for these reactions was demonstrated in comparison to previously reported magnetic Pd‐based catalysts.^[^ ^305^ ^]^ Kazemi et al. combined the advantages of polyoxometalates with those of magnetic NPs to produce a novel nanosorbent for the removal of MB. H_5_PMoV_2_O_40_ was immobilized on modified NiFe_2_O_4_ NPs and the catalyst could accelerate electron transfer in photocatalytic properties due to the good dispersion of PMoV on the surface of the nickel ferrite. A bond formation between hydroxyl groups on the surface of the magnetic component and metal ions in Keggin‐type polyoxometalate took place.^[^ ^306^ ^]^

Sulfur‐doped SnFe_2_O_4_/graphene nanohybrid composites were produced via a facile low temperature solvothermal method by Jia et al. Thiourea was used as the S source to prepare S‐doped tin ferrite NPs with controlled sulfur concentration through a hydrothermal protocol. These nanocomposites demonstrated a much better activity in the photocatalytic degradation of chlorotetracycline in comparison to their single components.^[^ ^307^ ^]^ Another study illustrated that the loading of Ag on Fe_3_O_4_/g‐C_3_N_4_ nanocomposites greatly enhanced the catalytic activity of the composite in what concerns the photocatalytic degradation of MB. The combination of Ag with magnetite has been used to accelerate the photocatalytic property of Fe_3_O_4_ NPs since the Ag NPs display an increased hole separation and interfacial charge transfer ability. They also help to increase the visible light excitation of Fe_3_O_4_. The addition of silver assisted to improve the antibacterial efficiency of the composite against *E. coli*.^[^ ^308^ ^]^ In another report, Ag NPs were grown onto the surface of mesoporous Fe_3_O_4_@C using silver acetate as precursor with the assistance of ultrasound treatment. The Ag‐(Fe_3_O_4_@carbon) composites possessed good antibacterial properties for *E. coli* and *Staphylococcus aureus* as well as high catalytic performance for the 4‐NP reduction, being also magnetically separable.^[^ ^309^ ^]^ Abbasi and co‐workers exfoliated graphene nanosheets and they decorated them with AgFeO_2_ nanocrystals in a homogeneous way, using a one‐step hydrothermal process. The AgFeO_2_‐G nanocomposites showed high electrocatalytic activity for the degradation of RhB, MO, and MB in the presence of visible light irradiation.^[^ ^310^ ^]^ In another study, Ag/TiO_2_/Fe_3_O_4_ composites displayed higher photocatalytic activity and MO removal efficiency than TiO_2_ or Ag/TiO_2_ alone under identical experimental conditions.^[^ ^311^ ^]^

Magnetically separable Pd NPs were designed on sustainable pectin‐carboxymethyl cellulose composite (Pct‐CMC) and the final Pd@Pct‐CMC/Fe_3_O_4_ composites were evaluated for Suzuki–Miyaura and 4‐NP reduction reactions. The Pd NPs were successfully immobilized and homogeneously dispersed on Pct‐CMC/Fe_3_O_4_.^[^ ^312^ ^]^ A well dispersed magnetically separable Pd complex immobilized on Fe_3_O_4_‐GO nanocomposite was prepared with a solvothermal approach. The distribution of the magnetite and of the palladium complex on graphene sheets was uniform. The composites were employed as catalysts for the direct aerobic oxidative esterification of alcohols with methanol in an environmentally friendly procedure.^[^ ^313^ ^]^ An assembly process of magnetic NPs has been introduced by Kuchkina et al. to obtain magnetically recoverable catalysts. Pd NPs were grown in dendron/dendrimer shells of multicore magnetite structures. These composites were used for the selective hydrogenation of dimethylethynylcarbinol to dimethylvinylcarbinol. The dendrimer generation which determined the specific sites for the growth of the Pd NPs as well as their size is a crucial parameter that influenced their catalytic activity. These nanocomposites exhibit high catalytic performance and fast recovery in the presence of an external magnetic field and the best catalytic activity was found for Pd NPs size of 1.5 nm.^[^ ^314^ ^]^ Amali et al. prepared a composite which combines inorganic NPs (SiO_2_, Pd, Fe_3_O_4_) and a polymeric component (poly‐_L_‐lysine) in such a way that they are interspersed with each other to hold the catalytic Pd and magnetic Fe_3_O_4_ NPs in a well‐defined structure. The polymer stabilized the Pd NPs. The final composites were used as magnetically separable catalysts for selective hydrogenations.^[^ ^315^ ^]^ In a different approach, *Withania coagulans* leaf extract was used as a reducing and stabilizing agent for the green two‐step synthesis of Pd/RGO/Fe_3_O_4_ nanocomposites. The 4‐NP reduction catalysis was investigated using these composites.^[^ ^316^ ^]^ A cost effective one‐pot strategy for the decoration of small Ni NPs onto RGO sheets was presented by Hussain et al. These composites demonstrated excellent catalytic activity for the Sonogashira cross‐coupling reaction.^[^ ^317^ ^]^

**Figure 12:**
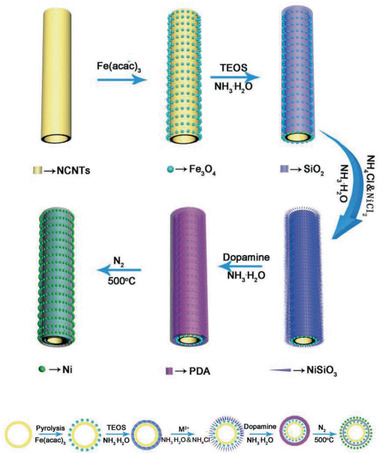
Schematic presentation of the fabrication of hierarchically structured NCMTs@Fe_3_O_4_@SiO_2_@C/Ni composites. Reproduced with permission.^[^ ^320^ ^]^ Copyright 2018, Royal Society of Chemistry.

The catalytic activity of nanocomposites is often improved in the case of supported catalysis. A magnetic cellulose aerogel‐supported Fe_3_O_4_ NPs composite was designed as a highly efficient and eco‐friendly catalyst for Fenton‐like degradation of RhB. The composites showed higher catalytic performance than that of the sole Fe_3_O_4_ NPs.^[^ ^318^ ^]^ A clay mineral, rectorite, was used as a support to prepare Fe_3_O_4_/rectorite composite catalysts in an in situ precipitation oxidation reaction. The immobilization of magnetite NPs on Al‐rectorite support improved the catalytic activity and prevented co‐aggregation.^[^ ^319^ ^]^ Miao et al. fabricated hierarchical magnetic metal silicate hollow microtubes using SiO_2_‐coated magnetic N‐doped carbon microtubes (NCMTs@Fe_3_O_4_@SiO_2_) as a chemical template. Further steps involving PDA and carbonization treatments (**Figure** **12**) led to the production of NCMTs@Fe_3_O_4_@SiO_2_@C/Ni‐Co‐Cu composites which were applied to catalyze the reduction of 4‐NP. The hierarchical structure of the final composite, its large specific surface area and the high density of metal NPs endowed an ultra‐high catalytic activity compared to that of Cu and Co supported catalysts.^[^ ^320^ ^]^ In another work, Fe(0)@Fe_3_O_4_ composites were produced, with the zerovalent Fe NPs being tightly attached to the surface of the Fe_3_O_4_, hindering the aggregation and passivation problems of Fe(0). The magnetite support accelerated the electron transfer to target pollutants and it improved the stability of iron NPs. Magnetite is a “green” support material which can provide high surface redox activity, strong electron transport capability, and good compatibility with different substrates. The degradation efficiency of the above composite toward decabromodiphenyl ether (BDE209) was investigated.^[^ ^321^ ^]^ Sun and Chen described the synthesis of poly(*o*‐toluidine)(POT)/Fe_3_O_4_/Au nanocomposites and they studied their catalytic activity for 4‐NP reduction. The reactivity of POT toward Au ions helped for the decoration of the initial composites with Au NPs.^[^ ^322^ ^]^

Li and co‐workers reported an approach to deposit trimetallic 10‐nm Au‐Pd‐Fe_3_O_4_ NPs on graphene. The composites displayed a high activity for the oxidation of TMB in comparison to bimetallic NPs or single component NP‐decorated graphene hybrids.^[^ ^323^ ^]^ Tabit et al. presented the synthesis of CoFe_2_O_4_/GO composites and they used them as heterogeneous peroxymonosulfate activators for the decomposition of RhB. The strong interfacial interaction between GO and cobalt ferrite provoked a synergistic function of large surface area and improved the electron transport ability and chemical reaction sites.^[^ ^324^ ^]^ In another report, APTES‐functionalized Fe_3_O_4_ microspheres‐supported Cu atom‐clusters (Cu‐APTES@Fe_3_O_4_) were synthesized. Excellent catalytic activity for the reduction of 4‐NP to 4‐AP was demonstrated for this composite. APTES played a crucial role as link‐bridge to preserve the copper self‐agglomeration and at the same time ameliorated the 4‐NP adsorption on the catalyst surface.^[^ ^325^ ^]^

A self‐assembly method was used to synthesize charcoal‐supported Pt/Fe_3_O_4_ composites with an average particle size of around 100 nm for the transfer hydrogenation of cinnamaldehyde with high chemoselectivity toward cinnamyl alcohol. The different reduction potentials between Pt(IV) and Fe(II) precursors were exploited.^[^ ^326^ ^]^ In supported catalysis, the choice of the solid support is important to obtain high conversion, great selectivity, and reusability. Bououdina and colleagues found out that the availability of basic sites of Fe_3_O_4_ solid support to attract the substrate (quinoline) and interaction with Rh NPs seems to favor good catalytic performance and outstanding reusability without deactivation. The Rh@Fe_3_O_4_ composites were used for the efficient hydrogenation of several N‐bearing heterocyclic acids and other aromatic compounds under mild experimental conditions in water.^[^ ^327^ ^]^ In another study, direct carbonization of cobalt complexes resulted in the preparation of magnetic carbon/cobalt nanocomposites suitable for heterogeneous catalysis of versatile catalytic reactions.^[^ ^328^ ^]^ A one‐step solvothermal approach with Co(OH)_2_ as cobalt source was used to synthesize Co NPs@RGO composites. The growth of Co NPs and the reduction of GO took place simultaneously. These composites were used to catalyze the reduction of highly toxic Cr(VI) to non‐toxic Cr(III) with easy recovery and recyclability.^[^ ^329^ ^]^

Heidari et al. highlight the superior functionality and excellent mechanical, electronic, optical, and thermal properties of GO sheets that could be useful in the design of new catalysts. They prepared M‐GO/(AM‐MBA‐*β*‐CD@Pd)(M‐GO, magnetic GO; AM, acrylamine; MBA, methylenebisacrylamide) nanocomposites and they used them as recyclable catalysts for Suzuki–Miyaura cross‐coupling reactions of aryl halides with boronic acids as well as for modified S‐M cross‐coupling reactions of *N*‐acylsuccinimides and boronic acid in green media.^[^ ^330^ ^]^ Another work presents the synthesis of Pd@salen‐porphyrin conjugated microporous polymer (Pd@SP‐CMP) composites. The functional CMP was used as a Pd NP support due to the coordinate interactions between the polydentate chelating sites with Pd(OAc)_2_ and subsequent reduction with NaBH_4_. Excellent performance and good recyclability for Suzuki–Miyaura and Heck–Mizoroki catalytic coupling reactions in water or dioxane/water mixture was evidenced for those composites.^[^ ^331^ ^]^ Zeolite‐supported nano iron–nickel bimetallic composites (Z‐nZVI‐Ni) were produced with a liquid‐phase reduction method. A very good dispersion of Fe‐Ni bimetallic NPs onto the zeolite sheet was shown. The slow iron and nickel leaching provided better stability and higher catalytic performance for the degradation of trichloroethene in groundwater.^[^ ^332^ ^]^ Magnetic zeolite obtained from iron and steel industrial waste was used as the support of TiO_2_ NPs. The advantages of using zeolite as a support were listed (e.g., high surface to volume ratio, functionality, porosity, thermal stability, adsorption capacity). The photocatalytic activity of these composites for the degradation of MB was evaluated.^[^ ^333^ ^]^ Zeolite‐supported nano Fe(0)‐Cu bimetallic composites were produced using an ion exchange method for application in the degradation of trichloroethylene.^[^ ^334^ ^]^ A facile solvothermal approach was used to synthesize magnetic Ni@h‐BN composites. Ni NPs were supported on hexagonal boron nitride nanosheets. The kinetics of ammonia borane hydrolysis was studied with the use of those composites as catalysts. The supported Ni NPs displayed a much better catalytic performance than the sole Ni NPs and the catalytic activity depended on the Ni amount loaded.^[^ ^335^ ^]^

Therefore, we notice that magnetic nanocomposites, synthesized through several ways and being either “unsupported” or supported, present very interesting catalytic properties. Synergistic effects, being present due to the interface between the different parts of a composite, lead to the appearance of improved or new features. In this way, the resulting properties are not only a “sum” of the properties of the individual components, but in addition enhanced or new properties may also appear. In supported catalysis, the selection of the solid support is critical to achieve high conversion, great selectivity, and reusability. Supports often provide a high surface area, good thermal and chemical stability, adsorption capacity, high electron transport ability, as well as porous channels which facilitate fast in‐pore transport of substrate and products in catalysis. Thus, the composite becomes accessible for reactants. The resulting composites can be applied as excellent magnetically separable catalysts.

### 4.2 Water Purification

The elimination of various types of contaminants such as heavy metals, organic compounds, and bacteria from aqueous media is one of the dominant applications of magnetic nanocomposites. Mesoporous Fe_3_O_4_@SiO_2_@KIT‐6(molecular sieve) nanocomposites were synthesized by Yang and colleagues and used as sorbent in magnetic solid phase extraction (MSPE). Trace pyrethroid pesticides in water were detected followed by high‐performance liquid chromatography, in a simple, easy, environmentally benign, sensitive, and precise method.^[^ ^336^ ^]^ Sulfur‐doped Fe_3_O_4_@SiO_2_‐amide‐linked organic polymer (S‐MAOP) was prepared by chemically anchoring AOP onto the surface of NH_2_‐functionalized magnetic NPs. The final composites displayed much higher adsorption selectivity and affinity for Hg(II) uptake from water solution than the initial MAOP due to introduction of thiomide groups in the network.^[^ ^337^ ^]^ A series of nano‐sized Zr‐magnetic MOF composites including Fe_3_O_4_@SiO_2_@UiO‐66‐NH_2_ and others were prepared with a facile one‐pot hydrothermal approach. The nanocomposites were efficient for the removal of heavy metal ions (Pb^2+^) and to capture organic dyes from water (MO and MB) with high adsorption capacity and fast adsorption kinetics.^[^ ^338^ ^]^ Biosorbents composed of magnetite NPs functionalized by *κ*‐carrageenan hybrid siliceous shells (Fe_3_O_4_@SiO_2_/SiCRG) were produced and evaluated in magnetically assisted removal of metoprolol tartrate from aqueous medium. The improved efficiency in the adsorption capacity of this material compared to other sorbents was attributed to the high affinity of sulfonate groups from *κ*‐carrageenan to metoprolol molecules, along with small particle dimensions and high surface‐to‐volume ratio.^[^ ^339^ ^]^ In another report, sulfur‐doped TiO_2_ (TiO_2_‐S) photocatalyst was fabricated for photocatalysis using titanium sulfate as a dual precursor for both TiO_2_ and S in a one‐pot protocol. Pre‐synthesized Fe_3_O_4_ NPs were coupled on TiO_2_‐S through a hydrothermal process. The resultant Fe_3_O_4_/TiO_2_‐S composites contained numerous surface hydroxyl groups and acted as efficient photocatalysts for the decomposition of RhB and formaldehyde solution under visible light and solar light irradiation. An electronic interaction‐induced synergistic effect of the surface hydroxyl groups and S atoms was found to reduce the band gap of TiO_2_, making it responsive to visible light.^[^ ^340^ ^]^ Magnetic activated carbon particles (MACP) were produced with a facile co‐precipitation process and they were applied as an adsorbent to remove Cu ions from water. The Fe_3_O_4_ loading onto the C matrix was crucial for the efficient removal of copper ions. A scalable adsorption process with a facile operating approach, high removal/regeneration performance, and relatively low cost were demonstrated for these composites.^[^ ^341^ ^]^ In another report, the synthesis of magnetic AC and magnetic biochar was presented. The impregnation of AC and biochar with magnetite was based on the co‐precipitation of ferrous and ferric salts. The composites were used to remove phenol from water, and their enhanced removal efficiency was assigned to the increase of specific surface area and pore volume due to magnetic modification.^[^ ^342^ ^]^ Sheshmani et al. fabricated magnetic graphene/chitosan nanocomposite via a facile chemical route and applied this material as an adsorbent for acid orange 7 removal. Characterization with various techniques revealed plenty of possible interactions/forces of dye‐composite system. The composites showed a good and versatile adsorption capacity for the aforementioned dye and could be easily manipulated by means of magnetic attraction.^[^ ^343^ ^]^ Wang and co‐workers published a work on the synthesis of Fe_3_O_4_@G(graphene) nanocomposites through an in situ precipitation method and they employed these materials to remove oxotetracycline and tetracycline from aqueous solution. The removal efficiency in lake, tap, and pool water was evaluated. High adsorption efficiency of the tetracycline antibiotic was evidenced.^[^ ^344^ ^]^

Zhang et al. synthesized Fe_3_O_4_@RGO nanocomposites by a co‐precipitation method and applied them to remove bisphenol A (BPA) from water in a recyclable manner. Excellent performance for BPA adsorption was evidenced, and the loading amount of MNPs affected drastically the adsorption activity.^[^ ^345^ ^]^ In another study, dithiocarbamate (DTC)‐modified magnetic reduced graphene oxide (RGO‐PDTC/Fe_3_O_4_) nanocomposites were synthesized for the removal of heavy metal ions (Cu(II), Cd(II), Pb(II), and Hg(II)) in synthetic wastewater. The composites were produced through a synthesis method that included GO bromination, nucleophilic substitution of polyethyleneimine (PEI), reaction with carbon disulfide (CS_2_), and magnetite NP loading. Large adsorption capacities with fast kinetics and solid–liquid separation were demonstrated.^[^ ^346^ ^]^ Magnetic graphene oxide modified by 2‐pyridinecarboxaldehyde thiosemicarbazone groups (Fe_3_O_4_@GO/2‐PTSC) was used to determine Hg(II) ions in trace amounts by inductively coupled plasma‐optical emission spectroscopy (ICP‐OES). The combination of sonication with MSPE helped to accelerate the magnetic adsorption process.^[^ ^347^ ^]^ Hu et al. employed an environmentally benign process to produce PLL‐functionalized magnetic Fe_3_O_4_‐(GO‐MWCNTs) hybrid composites with large surface area and plenty of hydroxyl and amino groups. First, MWCNTs were hybridized with GO by a chemical‐free method. Fe_3_O_4_ was then combined with GO‐MWCNTs by chemical co‐precipitation, and afterward PLL was grafted onto Fe_3_O_4_‐(GO‐MWCNTs) by chemical bonding. The composites were applied for the removal of tartrazine dye and Pb(II). High adsorption capacity, recyclability, rapid separation, and short operation time was illustrated.^[^ ^348^ ^]^ The KOH activation method was used to prepare Fe_3_O_4_@MWCNTs nanocomposites in a simple one‐pot approach. These materials displayed very high adsorption capacities for toluene, ethylbenzene, and xylene pollutants, which were higher than many other adsorbents, thanks to their large specific surface area and high degree of surface activity. The aforementioned activity constitutes a green route for MWCNT use in wastewater treatment.^[^ ^349^ ^]^ Lu et al. produced Fe_3_O_4_@MWCNTs with a facile one‐pot solvothermal synthesis using 1,6‐hexanediamine functionalized Fe_3_O_4_ NPs, for the removal of Cr(VI) from water. The composites showed excellent selectivity and high anti‐interference ability when confronted with commonly coexisting ions.^[^ ^350^ ^]^

Ampholytic polyelectrolyte microspheres with magnetite NPs embedded in the composite matrix were produced using an emulsification procedure from a homogeneous chitosan/carrageenan solution in LiOH/KOH/urea aqueous system. These materials displayed good compatibility and homogeneous network structure. The nanocomposites demonstrated a highly efficient adsorption capacity toward both cationic and anionic dyes as well as heavy metal ions in wastewater, due to their strong electrostatic and chelating affinity. The ampholytic chitosan/carrageenan composite matrix was responsible for the pollutant adsorption, while the Fe_3_O_4_ NPs helped for the magnetic separation. The pollutants tested were MB, Congo red (CR), copper nitrate, and chromic nitrate.^[^ ^351^ ^]^ An in situ method was employed to synthesize MOF‐199/Fe_3_O_4_ nanocomposites at room temperature. Electrostatic interaction facilitated the chemical stabilization of NPs and metal ions. These composites were investigated for their ability to adsorb neonicotinoid insecticides as hybrid adsorbents from environmental water. The developed method had excellent sensitivity, a wide linear range, and simple operation, together with satisfactory recoveries and repeatability under optimized conditions. The magnetic sorbent can be easily and rapidly isolated from aqueous medium by applying external magnetic field, thus avoiding a time‐consuming column passing procedure in SPE.^[^ ^352^ ^]^ MOF‐199/dithiocarbamate‐modified magnetite NP composite was employed for speciation analysis of As(III) and As(IV) via determination by electrothermal atomic absorption spectrometry. The dithiocarbamate functionalization endowed the nanocomposite with selectivity toward As(II) species. The developed method was fast, simple, selective, accurate, and precise, with fast extraction time, low detection limit, and high enrichment factor.^[^ ^353^ ^]^ Atta et al. doped copper oxides onto Fe_3_O_4_ NPs into hydrogels based on sodium 2‐acylamido‐2‐methylpropanesulfonate (AMPS) and *N*‐isopropylacrylamide NIPAm copolymers. These composites were used for the removal of MB. The AMPS/NIPAm hydrogel was chosen due to amphiphilicity as well as pH and temperature sensitivity to external environments together with its reusability for multiple adsorption–desorption cycles. Short contact time, high adsorption capacity, and stability were also achieved.^[^ ^354^ ^]^ In another study, Fe_3_O_4_/Cu_2_O/PANI nanocomposites with diverse functionality were fabricated by a one‐step in situ polymerization using cupric nitrate as the oxidant for aniline in the presence of magnetite NPs. A porous dandelion‐like nanocomposite with relatively low PANI content acted as a fast and very efficient sorbent for the selective removal of CR and MO from a simulated sewage system.^[^ ^355^ ^]^

Bentonite‐Fe_3_O_4_‐MnO_2_ nanocomposites have been synthesized by combining bentonite with magnetite and manganese dioxide through co‐precipitation. The composites were used to remove Cd(II) from aqueous media. The adsorption capacity was found to increase by raising the pH. The bentonite offered large specific surface area, low cost, chemical and mechanical stability, cation‐exchange capacity, and good adsorption capability.^[^ ^356^ ^]^ Luo et al. produced environmentally friendly millimetre‐scale magnetic cellulose beads with micro‐ and nanopore structure via an optimal extrusion dropping technology from NaOH/urea aqueous solution. The composite beads incorporated with carboxyl‐decorated Fe_3_O_4_ NPs and nitric acid‐modified AC showed high activity for the removal of Cu(II), Pb(II), and Zn(II). Relevant experiments illustrated that the adsorption processes were spontaneous endothermic reactions, controlled by combining physical and chemical adsorptive mechanisms.^[^ ^357^ ^]^ Zhang et al. prepared three different magnetic phosphate nanocomposites (Fe_3_O_4_/Ba_3_(PO_4_)_2_, Fe_3_O_4_/Sr_5_(PO_4_)_3_(OH), and Fe_3_O_4_/Sr_5x_Ba_3x_(PO_4_)_3_(OH) and explored their applicability for the removal of Pb(II) and malachite green (MG). The simultaneous removal of both types of contaminants was suggested to be due to ion exchange between Pb^2+^ and Sr^2+^ in the lattice and then the formation of hydrogen bonds between PO_4_^3−^ outside the material's surface and positively charged hydrogen in MG. The phosphate role was crucial for the overall removal performance.^[^ ^358^ ^]^ Au and Fe_3_O_4_ NPs were modified on a GO surface via light reduction and covalent attachment. The obtained Fe_3_O_4_‐Au‐GO nanocomposites were capable for the treatment of oil separation and dye decomposition at the same time.^[^ ^359^ ^]^ In another report, magnetite NPs were attached to the dead and alkaline activated biomass of *Aspergillus niger*. These nanocomposites were employed for Cr(VI) sorption, and it was shown that this sorption on the *A. niger* biomass was probably a physical process. This low cost activity featured a high sorption rate due to the low activation energy of adsorption and high density of the active sites on the adsorbent surface which removed about 70% of Cr(VI) within 1 min.^[^ ^360^ ^]^

Chen et al. coated Fe_3_O_4_ NPs with a polystyrene (PS) layer to form water‐repellent and oil‐absorbing surfaces. The resulting Fe_3_O_4_@PS composites were able to separate fast and efficiently oils from water surface using a magnetic field. Excellent recyclability was observed, with no production of secondary pollution to the environment.^[^ ^361^ ^]^ In another report, ZIF‐67 (zeolitic imidazolate framework‐67) nanocrystals were chosen as an attractive subfamily of MOF to produce Fe_3_O_4_‐PSS(poly(styrenesulfonate, sodium salt)@ZIF‐67 composites. These highly porous materials with unsaturated cobalt sites were employed for MO adsorption, showing very high capacity.^[^ ^362^ ^]^ Peng et al. prepared Fe_3_O_4_@silica‐xanthan gum composites by fixing xanthan gum, which is a natural polymer, on the surface of Fe_3_O_4_ microspheres through a sol–gel process. The composites were used for the removal of Pb(II) from aqueous medium. The xanthan molecules consisted of repeating five‐sugar units and they could selectively coordinate with the targeted Pb(II) ions. Fast adsorption rate with recyclability for over 21 cycles was observed, with no significant capacity loss.^[^ ^363^ ^]^ An ex situ precipitation and external reduction method was employed to intercalate Fe_3_O_4_ NPs onto a polyethylene terephthalate activated carbon matrix. The final composites possessed high surface area and they were used for the adsorption of cephalexin, demonstrating high adsorption capacity.^[^ ^364^ ^]^ In another work, Fe_3_O_4_‐*β*‐cyclodextrin‐MWCNT composites were produced from the reaction of oxidized CNT with cyclodextrin in the presence of hydrazine hydrate, with subsequent attachment of this composite to the magnetite NPs. The composites were applied to extract several different polycyclic aromatic hydrocarbons (PAHs) from environmental water samples. Applying a “micro” MSPE technique with these composites as sorbent constituted a sensitive, simple, and fast method with a wide linear dynamic range for the extraction of PAHs.^[^ ^365^ ^]^

**Figure 13:**
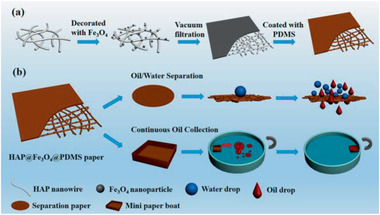
a) Schematic representation of the fabrication process of the HAP@Fe_3_O_4_@PDMS paper and b) its application as a filter paper for oil/water separation and magnetically driven oil collection. Reproduced with permission.^[^ ^368^ ^]^ Copyright 2018, American Chemical Society.

Zhai et al. prepared Fe_3_O_4_‐1,5‐diphenylcarbazide(DPC) nanocomposites and they used them as extractants for the solid‐phase extraction and preconcentration of trace Hg(II) from aqueous medium. This method to recover Hg(II) was simple, inexpensive, and eco‐friendly and offered high adsorption capacity. DPC was selected due to its favorable coordination capacity and selectivity for mercury ions.^[^ ^366^ ^]^ In another work, the incorporation of magnetite NPs into cryogels by an in situ approach was suggested, aiming to increase the dispersion of NPs in the gel composites and to produce effective magnetic materials with high adsorption capacities. 2‐acrylamido‐2‐methylpropane sodium sulfonate (Na‐AMPS) as well as *N*,*N*‐methylenebisacrylamide (MBA) were used as ionized and non‐ionic monomer and cross‐linker, correspondingly, to produce cryogel homopolymer or copolymers with either N‐vinyl pyrrolidone (VP) or 2‐hydroxyethyl methacrylate. The magnetite cryogel composites demonstrated greatly enhanced MB dye removal in short times with higher adsorption capacities than other potential adsorbents and good regeneration.^[^ ^367^ ^]^ Another study discusses the fabrication of hydroxyapatite (HAP)@Fe_3_O_4_@polydimethylsiloxane (PDMS) nanocomposites (**Figure** **13**). Free‐standing, recyclable, and fire‐retardant HAP nanowires were decorated with Fe_3_O_4_ NPs to endow magnetic property and a PDMS layer was added to offer a superhydrophobic behavior. These composites showed great potential for application in oil/water separation and oily wastewater treatment. Highly efficient oil collection, rapid transportation, and recyclability for at least ten successive cycles were achieved.^[^ ^368^ ^]^ In another report, hybrid nanocomposites (halloysite nanotubes [HNTs]@PANI@Cu) were prepared by coating Fe_3_O_4_‐loaded HNTs with PANI, followed by decoration with metallic Cu. The composites were used in a fast and sensitive ultrasound‐assisted magnetic dispersive solid‐phase microextraction setup for the determination of nitro‐phenanthrenes in environmental samples. The specific role of each one of the composite's components (magnetic HNTs, PANI, Cu) was described.^[^ ^369^ ^]^

**Figure 14:**
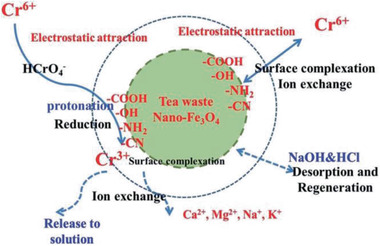
Proposed mechanism of Cr(VI) removal by the TW/Fe_3_O_4_ composite. Reproduced with permission.^[^ ^370^ ^]^ Copyright 2017, Royal Society of Chemistry.

Fan et al. produced tea waste/Fe_3_O_4_ nanocomposites through a facile chemical co‐precipitation approach. The composites were applied for the removal of Cr(VI), as shown in **Figure** **14**, which involved electrostatic attraction, reduction process, ion exchange, surface complexation, and other mechanisms. The adsorption of Cr(VI) was spontaneous in nature and endothermic.^[^ ^370^ ^]^ Deng et al. described a simple one‐pot solvothermal method to synthesize MIL‐101(Fe)@polydopamine@Fe_3_O_4_ composites from PDA‐modified magnetite NPs. This material was used as a magnetic adsorbent for the fast extraction of sulfonylurea herbicides from environmental water and vegetable samples. The composites presented combined advantages of Fe_3_O_4_ and MIL‐101(Fe). The MSPE extraction procedure was employed.^[^ ^371^ ^]^

In another work, Fe_3_O_4_@Prussian blue composites were prepared and used for water sterilization against gram‐positive (*S. aureus* and *E. coli*) bacteria. These magnetic photothermal materials provide a simple, recyclable, low‐cost, environmentally responsible, and highly effective route as a disinfection system.^[^ ^372^ ^]^ Jung et al. produced marine macroalgae‐derived AC/iron oxide magnetic composites (AC‐Fe‐MC) and they evaluated them for the removal of acetylsalicylic acid from aqueous media. The porous structure and superparamagnetic behavior of the composites allowed them to achieve an effective adsorption capacity and separation from the solution using an external magnetic field. This process may potentially be used in a large scale for isolating pharmaceutical compounds via using an inexpensive and easily accessible precursor.^[^ ^373^ ^]^ Canals and co‐workers reported the use of hexadecyltrimethylammonium bromide‐zeolite‐5/Fe_2_O_3_ composites as sorbents for magnetic dispersive solid‐phase extraction of nonsteroidal anti‐inflammatory drugs in water and urine samples with subsequent measurement by liquid chromatography diode array detection. The proposed method was simple, quick, economical, and user‐friendly. Time‐consuming filtration and centrifugation steps were redundant in that case.^[^ ^374^ ^]^ In another report, a co‐precipitation method was used to prepare natural kaolin/*γ*‐Fe_2_O_3_ composites. These materials had a mesoporous structure with a higher specific surface area than pure kaolin. This morphology endowed a significant enhancement in the removal capacity of phenol from aqueous media to the composite.^[^ ^375^ ^]^

Xiong et al. published a cheap method for the preparation of magnetic porous *γ*‐Fe_2_O_3_/C@HKUST‐1 composites for the removal of MB dye and Cr(VI). The MW‐enhanced high‐temperature method was used to prepare *γ*‐Fe_2_O_3_/C and then HKUST‐1 was grown onto the aforementioned particles using liquid‐phase epitaxy without chemical modification.^[^ ^376^ ^]^ In another study, Fe_3_S_4_/RGO nanocomposites were prepared and used to remove Pb(II) from water. For the synthesis of the composites, Fe_3_O_4_ NPs were first dispersed on RGO and afterward they were employed as sacrificial materials for a facile sulfuration process. The final composites were better in what concerns adsorption capacity and adsorption speed compared to Fe_3_O_4_/RGO. Synergistic contributions of the strong and selective Pb‐S interactions between Pb(II) and Fe_3_S_4_ NPs associated with the surface adsorption contributed toward an increase adsorption activity.^[^ ^377^ ^]^

Composites consisting of zeolite and montmorillonite functionalized with zerovalent Fe NPs were produced using an impregnating process with a solvent excess. These composites were employed for the sorption of Pb(II). High sorption capacities were assigned to the synergistic behavior between the clay coating and the Fe NPs.^[^ ^378^ ^]^ In a different report, a magnetic zerovalent iron‐carbonaceous conjugated microporous polymer nanocomposite (ZVI‐CCMP) was prepared from ZVI and waste polystyrene via a liquid phase reduction process. This material was applied for the low‐cost removal of dissolved organic carbon in water. The performance of the composite was much better than ZVI alone, demonstrating the synergistic effect of ZVI activation.^[^ ^379^ ^]^ Wang et al. used pine‐derived biochar as a support to stabilize Fe(0) NPs for As(V) removal. The nZVI/BC composites were fabricated by precipitating the NPs on carbon surfaces. The removal of arsenic ions was investigated in batch, continuous flow, and completely mixed reactors. Surface complexation seemed to be the principal removal mechanism, although a reduction reaction might be also involved.^[^ ^380^ ^]^ Iron‐aluminum oxide NPs anchored on graphene oxide (IAO/GO) was produced through a simple one‐step co‐precipitation method for fluoride removal from water. The combined advantages of GO and IAO favored a high adsorption capacity, good acid–alkali stability, superparamagnetic character, and good selectivity for fluoride. According to the sorption studies, the most possible mechanisms that were responsible for fluoride sorption were electrostatic attraction, anion exchange, and inner‐sphere complexation.^[^ ^381^ ^]^ Fang et al. have written a review article on nZVI composites with various supports and they discuss the removal mechanisms for the treatment of pollutants such as dyes, heavy metals, nitrogen, and phosphorus from water.^[^ ^382^ ^]^

Core–shell structured magnetic polyimide@layered double oxide (LDO) composites coating a porous polyimide (PI)‐coated Fe_3_O_4_ magnetic core and layer double hydroxide were prepared by solvothermal synthesis and co‐precipitation process. These flower‐shaped composites were used to remove tetracycline, 2,4‐dichlorophenol, and glyphosate from aqueous medium. The adsorption of the contaminants was spontaneous and endothermic and the whole process was cost‐effective.^[^ ^383^ ^]^ Moreover, CoFe_2_O_4_@SiO_2_‐nylon 6 composites were applied in a stir bar sorptive‐dispersive microextraction method to extract hydrophilic organic compounds. This method allowed lower extraction time and easier post‐extraction treatment compared to simple stir bar sorptive extraction or dispersive solid phase extraction methods. The high affinity of sulfonated compounds to polyamides helped for a good adsorption capacity. This method was environmentally friendly and it was tested for several water samples (sea, river, and swimming pool).^[^ ^384^ ^]^

Pd‐Fe_3_O_4_‐flyash composites prepared combining electroless and co‐precipitation techniques were used for the decoloration of aqueous solutions containing organic synthetic dyes. A post treatment with high surface area AC helped to effectively adsorb the intermediate products of dye decoloration and carbon leached out from the surface of flyash particles. The dye removal process was found to be fast and efficient. The utilization of flyash as a catalyst‐support in that work was considered to help bridge the gap between the thermal power plants and dye‐related industries.^[^ ^385^ ^]^ Wang et al. described the large scale synthesis of Ag NW/Fe_3_O_4_ NP composites where magnetite NPs are attached onto the silver nanowires in situ in TREG. The composites were dispersible in both water and organic solutions. Good catalytic activity for the reduction of MB dye was demonstrated.^[^ ^386^ ^]^ A simple one‐pot solvothermal route was used to immobilize MnFe_2_O_4_ microspheres on graphene nanosheets. The resulting MnFe_2_O_4_‐G composites were employed for the adsorption of glyphosate. The adsorption process was spontaneous, exothermic, and feasible in the range 5–45 °C. The suggested adsorption mechanism of glyphosate on those composites was a combination of electrostatic interaction with an ion exchange. An excellent glyphosate adsorption capacity with high adsorption rate was illustrated.^[^ ^387^ ^]^ Ellipsoid *α*‐Mn_2_O_3_@*α*‐μnO_2_ core–shell nanocomposites were fabricated through a hydrothermal approach followed by a calcination step. These core–shell composites were effective in the heterogeneous oxone activation to generate sulfate and hydroxyl radicals in order to degrade aqueous phenol. The catalytic activity was better than that of homogeneous Mn(II), or individual *α*‐Mn_2_O_3_ and *α*‐MnO_2_ catalysts.^[^ ^388^ ^]^ Lin et al. reported the preparation of a cobalt‐based magnetic carbonaceous nanocomposite (MCN) by one‐step carbonization of the cobalt‐containing MOF, ZIF‐67. The presence of Co_3_O_4_ in MCN endowed magnetic separation ability but also catalytic activity for the activation of oxone. The decolorization of RhB dye in water was investigated in the presence of the aforementioned composite. Several parameters of the catalytic process were studied and a good degree of recyclability was shown.^[^ ^389^ ^]^

Amidoximated microgels, amid‐p(Mac‐co‐AN) microgels, were used for in situ synthesis of Co‐Fe bimetallic magnetic NPs by simultaneous reduction of Co(II) and Fe(II) ions within microgel. The prepared magnetic microgel nanocomposites were used for the removal of Cd(II), Cr(III), MB, and rhodamine 6G dye, as well as of a herbicide, paraquat. Contaminated samples from tap, river, and seawater sources were investigated.^[^ ^390^ ^]^ Dai and Vogt produced mesoporous carbons containing Co NPs through self‐assembly of Pluronic F127, phenol‐formaldehyde oligomer (resol), cobalt acetylacetonate, and TEOS. The composites displayed an outstanding adsorption capacity for methylene green, which was enhanced by increasing the Co content.^[^ ^391^ ^]^ In another work, hydrochar‐derived pyrolysis char‐supported Cu nanocomposites (Cu/HDPC) were used for the degradation of octocrylene in water, in the presence of H_2_O_2_. The HDPC possessed relatively high surface area and pore volume and this led to a good dispersion of Cu NPs.^[^ ^392^ ^]^ TiO_2_/Au NRs composites were fabricated in a gram‐scale procedure via a co‐precipitation approach followed by a calcination treatment. This composite was used to degrade MB and an antibiotic, nalidixic acid. A high photocatalytic performance was evidenced.^[^ ^393^ ^]^ Some reviews on the use of metal oxide‐based and other nanocomposites for the removal of heavy metals and other pollutants from water and wastewater have already been published.^[^ ^394^, ^395^, ^396^, ^397^ ^]^

To summarize this section, magnetic nanocomposites have been largely used for water treatment, to tackle the presence of pollutants such as heavy metals, dyes, and bacteria. A variety of fabrication methods has been employed to obtain such nanocomposites. Often, characteristics as high adsorption capacity, recyclability, rapid magnetic separation, and short operation time were demonstrated. Small particle dimensions, large specific area, and high degree of surface activity were beneficial, compared to more traditional water purification agents. Other features may include low cost as well as chemical and mechanical stability. Adsorption processes can combine physical and chemical adsorptive mechanisms.

### 4.3 Sensing Applications

Magnetic nanocomposites have been also used as detection tools for a variety of compounds, in different environments. Acylated xylan‐based magnetic Fe_3_O_4_ nanocomposite hydrogels (ACX‐MNP‐gels) were produced by synthesizing Fe_3_O_4_ nano‐octahedra in situ within a hydrogel matrix. The latter was prepared by the co‐polymerization of ACX with acrylamide and N‐isopropylacrylamide under ultraviolet irradiation. The composites showed a sensitive response to H_2_O_2_ detection even at a very low concentration. A good magnetic/temperature dual‐response for the magnetic hydrogels was demonstrated.^[^ ^398^ ^]^ Min and Wenzhong reported the fabrication of core–shell–shell nanocomposites, with magnetite NPs being the inner core, mesoporous silica functionalized with phosphorescent Ru(II) complex as the outer shell, whereas amorphous silica was present as the middle shell. These nanocomposites were used as sensing probes for oxygen. The good photostability of the Ru‐MCM‐41@SiO_2_@Fe_3_O_4_ composites was attributed to the covalent bonding to excellent supporting matrix of MCM‐41. No slight photobleaching was noticed. The oxygen‐sensing performance was measured on the basis of steady emission intensity quenching.^[^ ^399^ ^]^ Amino‐modified fluorescent magnetic nanocomposites were prepared with a method involving the use of APS, Fe_3_O_4_ NPs, and CdTe quantum dots. These composites were applied for the high‐sensitivity detection of trace target DNA, with facile operation, easy enrichment, and separation capability by an external magnetic field.^[^ ^400^ ^]^ In another work, core–shell hybridized nanostructures of Fe_3_O_4_ NPs and poly(3,4‐ethylenedioxythiophene) (PEDOT)‐conductive polymers were used as high‐performance chemiresistive sensors to detect volatile organic compound (VOC) vapors. These magnetic nanocomposites were synthesized by a MW‐assisted method in the presence of polymerized ionic liquids (PILs), which acted as a linker to couple the magnetic NPs with PEDOT. The PILs played diverse roles, preventing the aggregation of magnetite NPs and imparting specific functionalities to their surfaces. High sensitivity of the hybrid PEDOT‐PIL@Fe_3_O_4_ nanocomposites to VOC biomarkers including methanol, ethanol, acetone, benzene, and toluene was evidenced, with a very low detection limit. Such reliable sensing activity is important for the early diagnosis of lung cancer, as the aforementioned biomarkers are present in the exhaled breath of lung cancer patients.^[^ ^401^ ^]^ Another study referred to the synthesis of composites consisted of Fe_3_O_4_ NPs and molecularly imprinted polymers (MIPs), through a surface imprinting method combined with precipitation polymerization. These materials were employed for the selective and sensitive fluorescent detection of 17*β*‐estradiol based on the competitive desorption of fluorescein as a fluorescent indicator from the composites. Fast binding kinetics, easy separation, and reusability were demonstrated. Overall, the detection method was simple, rapid, convenient, environmentally benign, accurate, and cost‐effective. Trace levels of the targeted compound could be detected. Spiked lake and river water samples were studied for that purpose.^[^ ^402^ ^]^

A hybrid material based on MIP‐decorated Fe_3_O_4_ NPs for specific and label‐free sulfonamide detection was presented by Merkoci and co‐workers. An extremely low limit of detection was shown, which was close to those obtained by liquid chromatography and mass spectrometry. This sensing system displayed selectivity and specificity and can be useful for seawater monitoring, since such samples contain a minimum sulfonamide amount compared to other environmental samples.^[^ ^403^ ^]^ Asfaram et al. published the use of Fe_3_O_4_‐HKUST‐1 MOF composite as a support for surface imprinting of gallic acid imprinted polymer (HKUST‐1‐MOF‐Fe_3_O_4_‐GA‐MIP) with vinyltrimethoxysilane (VTMOS) as the cross‐linker. The resulting nanocomposites were employed for the rapid, selective, and sensitive ultrasound‐assisted dispersive magnetic solid phase microextraction of gallic acid by UV–vis (UA‐DMSPME‐UV–Vis) detection method. A good detection selectivity was shown with remarkable precision and a low detection limit.^[^ ^404^ ^]^

Sotomayor and co‐workers published the synthesis of magnetic nanocomposites composed of Fe_3_O_4_ NPs and MIPs in a core–shell format, for the selective detection of 1‐chloro‐2,4‐dinitrobenzene, a powerful allergenic substance. The magnetite NPs were produced by a co‐precipitation method and mixed with OAc. This material was then encapsulated in three types of hydrophobic polymeric matrix by a mini‐emulsion method. These composites offer increased selectivity, durability, and possibility of reuse. High adsorption capacity was demonstrated.^[^ ^405^ ^]^ Furthermore, Fe_3_O_4_ NPs were in situ loaded on the surface of MWCNTs by a simple co‐precipitation procedure. The obtained Fe_3_O_4_/MWCNTs nanocomposites were applied for the selective detection of dihydronicotinamide adenine dinucleotide (NADH). High sensitivity, reproducibility, wide linear range, and minimal surface fouling were shown. The attractive low potential of the NADH oxidation was an important feature that rendered this nanocomposite electrode suitable for biosensing applications.^[^ ^406^ ^]^ Liu and Mao reported the synthesis of two fluorescent magnetic core–shell composites, denoted as core@MCM‐41/R6 and core@MCM‐41/RS6. Fe_3_O_4_ particles were used as core and silica molecular sieve (MCM‐41) was the shell. The composites were employed as nitrite optical sensing agents, and they achieved site‐specific guiding with the aid of a magnet.^[^ ^407^ ^]^ In another work, a ratiometric fluorescence sensor was produced via biotin–streptavidin interaction for the detection of *Bacillus thuringiensis* (Bt) special gene fragment. In that context, special green quantum dot‐decorated Fe_3_O_4_ magnetic beads fabricated with streptavidin (SA) acted as donor, whereas gold NP‐modified red quantum dots (rQDs@SiO_2_) with hairpin DNA acted as receptor. The proposed sensor exhibited a simple, fast, precise, and sensitive detection of tDNA sequence.^[^ ^408^ ^]^ Nanocomposites containing poly(bromocresol green), magnetite NPs, and MWCNTs were prepared in another study for the sensitive electrochemical detection of serotonin. The MWCNTs provided numerous active sites which increased the sensor sensitivity. A wide linear concentration range was shown for the composite‐modified electrode, together with a low detection limit, good selectivity, and reproducibility.^[^ ^409^ ^]^

Magnetite nanochains prepared by a co‐precipitation method in the presence of dextran were linked with chlorotoxin (CTX) and curcumin via the PEGylation and carbodiimide technique. The CTX‐NCs‐Cur composites were applied for dual modal imaging detection and limitation of early tumor. The cytotoxicity, targeting ability, imaging enhancement, and anti‐cancer activity of the composites were evaluated. MRI contrast enhancement and good fluorescence imaging were illustrated.^[^ ^410^ ^]^ Jie and Yuan published the facile preparation of a magnetic electrochemiluminescent Fe_3_O_4_@CdSe nanocomposite which was used for the sensitive and fast electrochemiluminescence detection of thrombin by a multiple DNA cycle amplification strategy for the first time. Sensitivity, specificity, and excellent activity in real human serum assay were displayed.^[^ ^411^ ^]^ PEI‐stabilized magnetite NPs were assembled with photosensitive nitric oxide (NO) donors (Fe_4_S_3_(NO_7_^−^)), RBS) and CuFL complex consisting of fluorescein derivatives (FL) and Cu(II) to produce Fe_3_O_4_‐CuFL‐RBS nanocomposites, under electrostatic interactions. This nanocomposite system could achieve light‐triggered NO release, in situ fluorescence turn‐on detection of NO and potential magnetism‐targeted delivery when utilized as smart carrier.^[^ ^412^ ^]^ Fayazi et al. produced HNT–iron oxide‐manganese oxide nanocomposite (HNTs‐Fe_3_O_4_‐MnO_2_) involving a combination of chemical precipitation and hydrothermal processes. These nanocomposites were used as solid‐phase extracting agent for the electrochemical detection of Hg(II). The suggested method had simple operation, good selectivity, high sensitivity, and reproducibility. Low cost and low detection limits were also achieved.^[^ ^413^ ^]^

**Figure 15:**
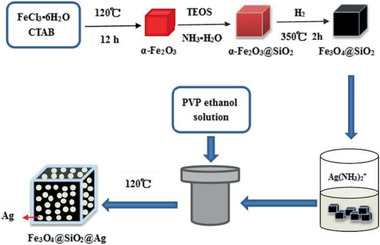
Synthetic process for cube‐like FSA composites. Reproduced with permission.^[^ ^415^ ^]^ Copyright 2016, Springer Nature.

In another study, a multi‐color fluorescent nanoprobe consisting of lanthanides and magnetic NPs (Fe_3_O_4_@CePO_4_:Tb‐EDTA‐Eu) was prepared. The composite was used for the sensitive, selective and point‐of‐care detection of dipicolinic acid (DPA), which is important for the prevention of the anthrax virus. The Fe_3_O_4_@CePO_4_:Tb part acted as the internal stable signal of green fluorescence whereas the EDTA‐Eu group was used as the sensitive reaction signal for DPA monitoring.^[^ ^414^ ^]^ Li et al. produced Fe_3_O_4_@SiO_2_@Ag nanocomposites by first preparing Fe_3_O_4_@SiO_2_ core–shell structures and then introducing the Ag NPs onto their surface via a one‐pot hydrothermal reaction (**Figure** **15**). The nanocomposites were applied for the detection of thiram, a dithiocarbamate fungicide pesticide used in agriculture. High SERS activity, stability, and strong magnetic responsivity together with low detection limit were evidenced.^[^ ^415^ ^]^ Mayr and co‐workers published the synthesis of composites containing Fe_3_O_4_ and NaYF_4_ which combined magnetic and upconversion luminescence properties. These materials were employed for oxygen sensing and being easily manipulated and separated through the application of an external magnetic field.^[^ ^416^ ^]^ Magnetic‐photocatalytic Fe_3_O_4_@SiO_2_@TiO_2_ nanocomposites were prepared through a solvothermal approach without any calcination step. These materials were used for the sensitive detection of several protein biomarkers such as alpha‐fetoprotein with the help of silicon microcantilever arrays. Even very low concentrations of human serum proteins could be detected.^[^ ^417^ ^]^

**Figure 16:**
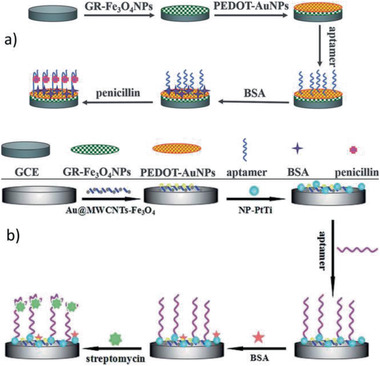
a) Schematic illustration of a penicillin aptasensor based on GR‐Fe_3_O_4_ and PEDOT‐Au NPs, b) a streptomycin aptasensor based on Au@MWCNTs‐Fe_3_O_4_ composite and nanoporous PtTi. a) Reproduced with permission.^[^ ^419^ ^]^ Copyright 2016, Royal Society of Chemistry. b) Reproduced with permission.^[^ ^429^ ^]^ Copyright 2016, Royal Society of Chemistry.

Multifunctional Fe_3_O_4_@SiO_2_@Au nanocomposites with superparamagnetic, fluorescent, and peroxidase‐like catalytic properties were prepared by Luo et al. in an aqueous solution. These composites were applied for the detection of H_2_O_2_ and glucose. The developed colorimetric‐visual method for glucose detection was simple, inexpensive, highly responsive and selective, with low detection limit.^[^ ^418^ ^]^ In another work, graphene‐Fe_3_O_4_ NPs were produced on a bare glassy carbon electrode and after that, a poly(3,4‐ethylenedioxythiophene)‐gold (PEDOT‐Au NPs) composite was also introduced onto the first film. The final composite was used to detect penicillin (**Figure** **16**a). The PEDOT‐Au addition helped to obtain a much larger surface area and a greatly improved electronic transmission rate. The final composite electrode had increased conductivity, excellent biocompatibility, and helped to achieve a low detection limit with high sensitivity.^[^ ^419^ ^]^

**Figure 17:**
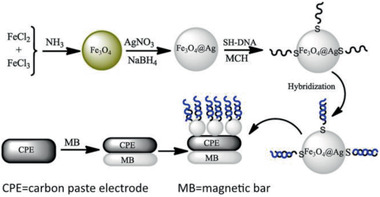
Schematic illustration of the fabrication of a DNA‐biosensor based on MBCPE/Fe_3_O_4_@Ag composites. Reproduced with permission.^[^ ^421^ ^]^ Copyright 2016, Elsevier.

Wu and colleagues reported the synthesis of PEI‐capped superparamagnetic iron oxide NPs by a solvothermal process and they managed the in situ self‐assembly of negatively charged gold NPs onto their surface. Further modification of the composites with 4‐mercaptobenzoic acid and folic acid‐conjugated rBSA allowed good specificity to HeLa cells. Detection of circulating tumor cells was achieved with these composites, with quite good detection limit.^[^ ^420^ ^]^ Benvidi and Jahanbani fabricated an electrochemical biosensor based on magnetic bar carbon paste electrode (MBCPE) modified with magnetite and silver NPs. The MBCPE/Fe_3_O_4_@Ag nanocomposites were employed for DNA detection (**Figure** **17**). A wide linear range and a low detection limit were observed. The biosensor possessed a high conductivity with large surface to volume ratio. The sensing method was fast, sensitive, stable, inexpensive, and showed excellent sensitivity for the detection of breast cancer mutation.^[^ ^421^ ^]^ Another study discusses the modification of a magnetic glassy carbon electrode (mGCE) with a ternary composite prepared from Prussian blue, magnetite, and RGO. The produced material was used for the sensing of hydrazine. The magnetite offered magnetic immobilization and separation ability, while the RGO provided an increased sensitivity. Low detection limit was achieved in that case.^[^ ^422^ ^]^

Fe_3_O_4_ and Cu NPs were integrated into nitrogen‐doped carbon nanotubes through a one‐pot high‐temperature decomposition procedure. Afterward, Au NPs were assembled on formed materials to generate NCNTs@Fe_3_O_4_@Au nanocomposites by galvanic replacement with Cu NPs. Cytochrome was accumulated by the composite and the resulting material displayed good activity on detection of H_2_O_2_. Synergistic improved sensitivity and very low detection limit were observed in a fast and convenient process.^[^ ^423^ ^]^ Lai et al. produced a ternary hybrid structure composed of Au NPs grown on a Fe_3_O_4_‐MoS_2_ microflower composite. The final product was used for SERS detection and degradation of aromatic dyes. The MoS_2_ helped to reduce and act as a support for the stabilization of Au NPs; it improved SERS sensitivity, provided a large specific surface area and helped to catalyze the degradation of the dye pollutant. The dyes studied were MG, MB, and RhB. Low detection limit and excellent reproducibility with a fast degradation of RhB were obtained.^[^ ^424^ ^]^

**Figure 18:**
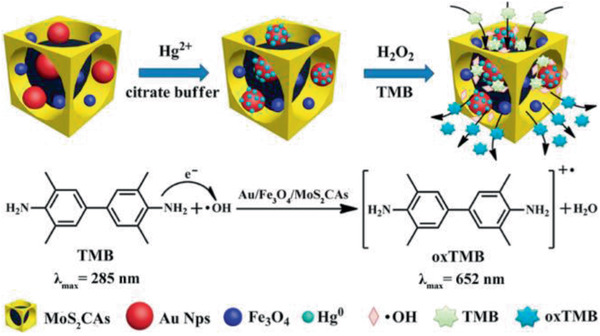
Detection of Au/Fe_3_O_4_/MoS_2_CAs for sensing Hg(II). Reproduced with permission.^[^ ^427^ ^]^ Copyright 2016, American Chemical Society.

In another work, well‐defined magnetic and thermal dual‐responsive nanocomposites were prepared, which contained core–shell Fe@SiO_2_ NPs as magnetic core and PNIPAM as thermosensitive outer shell. The nanocomposites were evaluated for the detection and removal of bisphenol A and other phenolic compounds. The simultaneous phenol determination was based on a MSPE approach. High sensitivity, high enrichment, quick analysis, reusability, and an eco‐friendly process were characteristics of that method.^[^ ^425^ ^]^ Liz‐Marzan and co‐workers published a simple way to co‐encapsulate Au nanostars and iron oxide NPs into hybrid colloidal composites. These materials possessed both plasmonic and magnetic properties and their SERS detection abilities were tested by comparing MG as a positively charged probe and trypan blue as a negatively charged analyte.^[^ ^426^ ^]^ Composite aerogels were fabricated using GO‐doped MoS_2_ sheets as the feedstock by hydrothermal assembly, and then Au and Fe_3_O_4_ NPs were embedded between the GO‐doped MoS_2_ sheets through coordination. The resulting Au/Fe_3_O_4_/MoS_2_CAs composites were applied for the detection of Hg(II) in water by a colorimetric method (**Figure** **18**). A low detection limit was observed with a very high adsorption capacity for mercury removal, which was superior than other materials.^[^ ^427^ ^]^

Hashemi et al. prepared a 3D‐magnetic RGO/polyaniline/Au NPs composite and immobilized a specific thiolated aptamer onto its surface. Then, they used the nanocomposite to modify screen‐printed carbon electrodes. The whole process aimed at cocaine detection, was repeatable, accurate, sensitive, and user‐friendly.^[^ ^428^ ^]^ In another paper, a Au@MWCNTs‐Fe_3_O_4_ composite together with a nanoporous PtTi alloy were placed on a glassy carbon electrode surface to prepare an aptasensor for the selective and sensitive detection of streptomycin (Figure 16b). The nanocomposites provided a smoothly conductive pathway for electron transfer due to the synergistic amplification effect of its three components. Wide linear range, shortened analysis time, and low detection limit were obtained.^[^ ^429^ ^]^

A supersensitive system for the detection of circulating tumor cells was developed by combining triangular Ag nanoprisms and superparamagnetic iron oxide NPs. The Ag particles were encoded with 4‐mercaptobenzoic acid and modified with reductive bovine serum albumin and folic acid. The final MBA‐AgNPR‐rBSA‐FA nanocomposites were SERS‐active and were applied for CTC detection in blood samples with good sensitivity and specificity, and with very low detection limit. Better sensitivity and specificity was observed for the composites in comparison to the SERS‐active nanostars alone.^[^ ^430^ ^]^ Xu et al. prepared core–shell fluorescent Ag@SiO_2_@SiO_2_‐RuBpy nanocomposites that were used for the detection of prostate‐specific antigen (PSA), which is the most specific and efficient tumor marker for the diagnosis of prostate cancer. The interaction between the doped RuBpy molecules in the outer SiO_2_ shell and the Ag core improved much the excitation efficiency and increased the fluorescence intensity. The existence of SiO_2_ decreased the self‐quenching of RuBpy, which helped to introduce larger amounts of RuBpy into the silica shell. The whole PSA detection process was sensitive, specific, reliable, easy to operate, and time‐saving. A good linear relationship between the fluorescence intensity and the concentration of PSA was shown.^[^ ^431^ ^]^ Spende and co‐workers published the fabrication of ZnO/Fe_3_O_4_ composites, which involved the dispersion of pre‐synthesized Fe_3_O_4_ NPs into alkaline zinc nitrate solutions. After annealing, the composites were decorated with Ag nanostructures by adding them into a silver nitrate/EG solution at 95 °C. The final Ag/ZnO/Fe_3_O_4_ nanocomposites were SERS‐active and used for the detection and quantitative analysis of uric acid in water. A good sensitivity was demonstrated, which improved the detection of uric acid compared to previous SERS reports.^[^ ^432^ ^]^

ZnFe_2_O_4_/polypyrrole nanocomposites were prepared by a method which included the chemical oxidative polymerization of pyrrole on zinc ferrite NPs surface. These composites were used for the detection of glucose with good sensitivity, stability, and quick sensing response. A linear concentration range and a low detection limit were observed.^[^ ^433^ ^]^ Zheng et al. produced CNTs/Fe_3_O_4_@PPy/Pd nanocomposites by first preparing CNTs/Fe_3_O_4_ and immobilizing PPy onto them, followed by accumulation of Pd NPs on the surface of the composites. These materials were applied for the detection of triclosan. The electrochemical sensing method used had quick response, low cost, simple operation, high sensitivity, and possibility for real‐time in situ detection. The PPy shell protects Fe_3_O_4_ from oxidation and corrosion while it can also be the carrier for the Pd NPs and improve the electrochemical performance of the sensor due to its high electrical conductivity. Pd NPs act as excellent scaffolds for the production of aptasensors, thanks to their biocompatibility, good conductivity, and outstanding photostability. Low detection limit was noticed for this system.^[^ ^434^ ^]^ Wang et al. fabricated hollow H‐Mn_3_O_4_‐PEI‐PtNPs‐Ru‐Eu^3+^ nanocomposites by first producing hollow Mn_3_O_4_ nanocrystals in the presence of triblock copolymeric micelles with core–shell‐corona architecture as templates. The peptide‐based biosensing of cyclin A_2_ (CA2), a prognostic cancer indicator, was achieved using these composites. A wide linear detection range was obtained, together with outstanding selectivity, stability, and reproducibility.^[^ ^435^ ^]^

In another work, Ni@graphene nanocomposites with core–shell morphology were prepared through thermal annealing of Ni‐BTC MOF. The composites were used to prepare a sensing platform comprising a magnetic glassy carbon electrode. Sensing of hydroquinone and catechol was achieved.^[^ ^436^ ^]^ A hydrothermal method was used to prepare NiO‐Nb_2_O_5_ nanocomposites which were applied for the detection of hydrogen at room temperature. The composites exhibited better sensing performance in comparison to pristine Nb_2_O_5_ in what concerns sensitivity, response time, and selectivity. The sensing mechanism of the NiO‐Nb_2_O_5_ nanocomposite‐based electrical H_2_ sensor was suggested to come from the combination of the modulation effect of the conduction channel width due to the oxidation of H_2_ with that of the potential barrier height due to the oxidation of H_2_.^[^ ^437^ ^]^ Several review papers have presented different types of nanocomposites (organic‐inorganic hybrids, metal‐dielectric systems, mesoporous silica, Au‐Ag/RGO) for sensing, diagnostic, and therapeutic applications.^[^ ^438^, ^439^, ^440^, ^441^ ^]^

Hence, we notice that magnetic nanocomposites are employed widely also as sensing agents for several different entities and in diverse environments. Again, the fabrication methods of those composites, this time with detection abilities, are numerous. Simple and quick operation, magnetic separation capability, sensitivity, selectivity, reusability, reproducibility, wide linear range, precision, real‐time in situ detection, as well as low cost are often some of the common characteristics of those systems. Low detection limits, sometimes comparable with those achieved by liquid chromatography or mass spectroscopy, can be also observed in certain cases.

### 4.4 Biomedical Applications

As shown before, magnetic nanocomposites are multi‐phase systems, organic–inorganic or all‐inorganic, which can combine the properties of their individual component materials. These complex, functional materials can be utilized in multiple biomedical applications. These multifunctional nanosystems, which are capable for diagnosis, drug delivery, and monitoring of therapeutic response, are expected to play a significant role in the dawning era of personalized medicine, and much research effort has been devoted toward that goal.^[^ ^442^ ^]^ In those systems, the nature of the different materials, the type of the ligand coating, the distance among the magnetic particles, the colloidal dispersion, the packing density, as well as the mutual NP interactions are parameters which play critical role in such applications.^[^ ^443^ ^]^ In the following, we will summarize the most important biomedical applications of the MNCs giving emphasis to their applications in MRI and multimodal imaging, in hyperthermia and drug‐delivery therapy, and finally in the application for biomolecule separation.

#### 4.4.1 Magnetic Nanocomposites as Effective MRI Contrast Agents

##### All‐Inorganic Nanocomposites

In this section, we will present the MRI efficiency of the systems, with either porous or complex structures, which possess at least two inorganic counterparts: the magnetic one and a second that could be Si, plasmonic, carbon‐based, or even another magnetic material.

Among these systems, a porous Si nanoparticle loaded with magnetic Fe_3_O_4_ NPs, namely as Fe_3_O_4_:pSi nanocomposite, was found to be a system with promising MRI capability.^[^ ^444^ ^]^ Their porous surface structure facilitates the proximal loading and alignment of magnetic NPs. Additionally, this particular surface structure provides control over the clustering of iron oxide NPs, yielding an increased magnetization compared to the individual particles. These structures showed enhanced magnetization if we compare with other systems such as the micelle‐covered nanocomposites of similar sizes, including similar quantity of iron. The Fe_3_O_4_:pSi nanocomposite with 25% loading of magnetic material has shown a T_2_‐value of 556 mm Fe^−1^ s^−1^, which is among the highest in the literature. This cell‐type structure also minimizes the cytotoxicity and long‐term tissue damage. No cellular (HepG2 or rat hepatocyte cells) or in vivo (rat) toxicity was observed, as this formulation degrades and eliminates after 4–8 h. In different studies, multiple core–shell structures with magnetic cores of manganites and ferrites have been combined with coating materials such as silica or titania.^[^ ^445^, ^446^, ^447^ ^]^ The regime of the transverse relaxation rate of ^1^H protons in aqueous suspensions has been studied for different sizes, morphologies, and shell features.^[^ ^445^ ^]^ Furthermore, nanocomposites with Janus morphology have been introduced also as MRI contrast agents. These systems can be used for theranostic purposes, as the Fe_3_O_4_ part can act as MRI contrast agent whereas TiO_2_ will help as an inorganic photosensitizer for photodynamic therapy.^[^ ^448^ ^]^

In another approach, magnetic–plasmonic nanocomposites have been also found that could act as efficient T_1_‐contrast agents with a very low toxicity. Such a nanocomposite system enables the growth of small iron oxide nanocrystals on a variety of plasmonic cores with the help of a thin polypyrrole interlayer as cohesive layer.^[^ ^449^ ^]^ Cores of different morphologies have been tested and among them the nanocomposite with an Au nanorod core has shown a low r_2_/r_1_ ratio of 4.8, making it an efficient T_1_ positive contrast‐enhancing agent for MRI. At the same time, this nanocomposite can behave synergically as an excellent photothermal agent due to their plasmonic core for cancer therapy in the second near infrared region, with a high photothermal conversion efficiency, reaching up to 46%. Furthermore, magnetic–plasmonic nanocomposites have also been synthesized in an inverse way, the magnetic part being in the core and the plasmonic in the shell in order to use them as multimodal theranostic nanoheaters in cancer photothermal therapy or for the thermal ablation of tumors.^[^ ^450^ ^]^ The advantage of the Janus configuration is also discussed in the case of the magnetic–plasmonic nanocomposites. Εasy and selective functionalization of each side, facile access of water to the magnetic surface—contrary to core–shell NPs—and plasmonic responses at the NIR biological transparency window are some among their advantages.^[^ ^166^ ^]^ Non‐spherical, star‐like structures have been evaluated for photoacoustic and SERS imaging at the same time with the MRI, which is not achieved using the conventional spherical Janus particles.^[^ ^166^ ^]^

**Figure 19:**
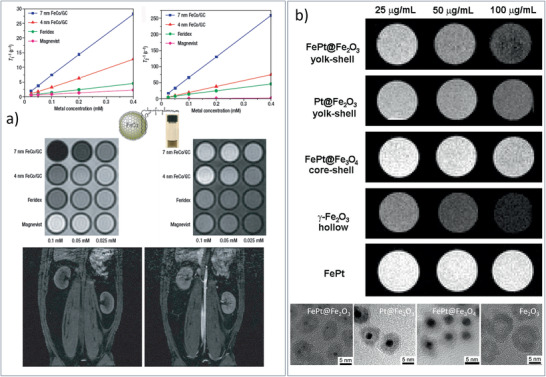
a) MRI positive and negative contrast efficiency of the FeCo/GC nanocomposites functionalized with phospholipid‐poly(ethylene glycol) of diameter 4 and 7 nm. T_1_‐weighted MR images of a rabbit before (left) and 30 min after (right) initial injection of a solution of ≈4 nm FeCo/GC nanocrystals.^[^ ^124^ ^]^ b) T_2_*‐weighted MR images of magnetic nanocomposites of different morphologies. a) Reproduced with permission.^[^ ^124^ ^]^ Copyright 2006, Springer Nature. b) Reproduced with permission.^[^ ^86^ ^]^ Copyright 2008, American Chemical Society.

Furthermore, graphene‐based magnetic nanocomposites were able to be used as a safe multifunctional nanoplatform for cancer theranostic applications.^[^ ^451^, ^452^ ^]^ For example, a graphene oxide–iron oxide–doxorubicin (GO‐IO‐DOX) system which combines two materials and a drug has been proposed for such purposes. The GO‐IO‐DOX nanocomposites were tested in vitro and were observed to exhibit an enhanced tumoricidal effect through both hyperthermia and cancer cell‐specific DOX release along with an excellent MRI performance, enabling a versatile theranostic platform for cancer. In a similar way, a core–shell FeCo/GC composite has been tested also as T_2_ MRI contrast agent (**Figure** **19**a).^[^ ^124^ ^]^ In this system, the shell is composed of only one layer in order to provide a very high r_2_ value and the FeCo core size was in the range of 4–7 nm. The FeCo/GC composites have been further functionalized with phospholipid‐PEG. The hydrocarbon chains of phospholipids were adsorbed onto the graphitic shells via van der Waals and hydrophobic interactions, whereas the hydrophilic PEG chain was extended into the aqueous phase to impart solubility. The relaxivity of the larger diameter core was found six times enhanced (644 mm ^−1^s^−1^ at 1.5 T) compared to the commercial T_2_ contrast agent Feridex. This high value was attributed to the high magnetization of the FeCo core and to the ultra‐thin, single‐graphitic shell structure affording effective magnetic relaxations to the proton spins around the nanocrystals. In addition, preliminary in vivo experiments in a rabbit with the functionalized FeCo/GC composites showed an exciting long‐lasting positive‐contrast intravascular MRI of the blood pool using a metal dose 10% of that typically used for existing Gd agents. The nanocrystals were stable in blood circulation for more than 20 min, much longer than conventional Gd agents that are known to allow for MRI only within seconds of injection before rapid leak‐out of Gd complexes from the blood vessels. The positive contrast enhancement was due to the unusually high r_1_ relaxivity of FeCo/GC nanocrystals. This result achieved a major goal of MRI contrast‐agent development, that is, long‐circulating positive contrast enhancement at low metal dosages in vivo. This has been difficult to attain with iron oxide NPs and will facilitate MRI angiography as well as various other applications.

Finally, nanocomposites of different magnetic materials have been tested as MRI contrast agents. FePt@Fe_2_O_3_ nanosystems of different morphologies functionalized with 3,4‐dihydroxy‐l‐phenylalanine (l‐dopa) molecules have been synthesized in order to study the effect of the morphology on the MRI efficiency.^[^ ^86^ ^]^ T_2_*‐weighted MR (magnetic resonance) images of FePt@Fe_2_O_3_ yolk–shell NPs, Pt@Fe_2_O_3_ yolk–shell NPs, FePt@Fe_3_O_4_ core–shell NPs, and *γ*‐Fe_2_O_3_ hollow NPs have been recorded in a 7.0 T MRI system (Figure 19b). The *γ*‐Fe_2_O_3_ hollow particles exhibit the strongest MR signal attenuation effect among the four types of nanocomposites. FePt@Fe_2_O_3_ and Pt@Fe_2_O_3_ yolk–shell nanocrystals also showed strong MR relaxation enhancement. However, FePt@Fe_3_O_4_ core–shell NPs and single‐phase FePt covered with cysteine exhibit a very weak MR contrast enhancement effect. The spin‐spin relaxation process of the protons in the water molecules surrounding the NPs is affected through the control of magnetic spins by changing the structure in the hollow or solid particles. The FePt@Fe_2_O_3_ yolk–shell NPs have shown a stronger MR contrast enhancement compared to the commercial contrast agents, MION and Sinerem, indicating their potential as novel, efficient MR contrast agents. The selective choice of the two magnetic materials in a core–shell structure could also modify the magnetic features of the nanocomposite. Α hard phase such as CoFe_2_O_4_ in the core could enhance the magnetic anisotropy, while a soft MnFe_2_O_4_ phase in the shell could enhance the magnetization.^[^ ^453^ ^]^ The same materials combined in compact bimagnetic nanoclusters in the presence of sodium dodecyl sulfate have shown improved hyperthermia efficiency and MRI contrast effect.^[^ ^454^ ^]^

##### Organic‐Inorganic Composites

**Figure 20:**
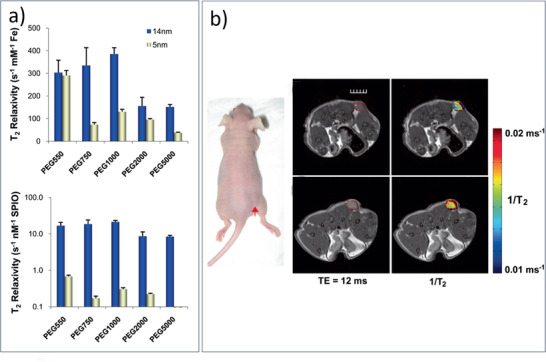
a) Dependency of T_2_ relaxivity of the core size and the PEG chain length of the SPIONs. b) In vivo tumor imaging (using spin‐echo sequence) using the 14 nm SPIONs. The arrow indicates the locations of the subcutaneous tumor. The pictures above show the MR images before the injection and the below the same tumor after 1 h following the injection. Reproduced with permission.^[^ ^459^ ^]^ Copyright 2010, American Chemical Society.

The MR relaxivities of such complex systems are dependent on several factors including the nature and number/size of the incorporated magnetic particles and also the type of the organic coating.^[^ ^455^, ^456^ ^]^ The choice of high‐quality magnetic particles with enhanced magnetic moment is important in order to acquire an enhanced MRI signal.^[^ ^457^ ^]^ Meanwhile, their surface coating is crucial for the promotion of the water diffusion and facilitates the water exchange between the magnetic NPs and the surrounding water molecules.^[^ ^458^ ^]^ Furthermore, the type of the surface coating can control the colloidal stability and prevent the particles from aggregation which could potentially reduce the MR relaxivities. While increasing the nanocomposite size could improve the MR relaxivities, the colloidal stability of the resultant large nanocomposites might be at risk. Thus, a balance between the aggregates size, stability, and relaxometric properties must be considered when designing efficient MRI contrast agents. The role of the capping organic agent in the MR contrast efficiency has been evaluated in superparamagnetic iron oxide nanoparticles (SPIONs) of 5 and 14 nm diameter covered with an organic shell of different thickness (**Figure** **20**).^[^ ^459^ ^]^ Iron oxide particles were coated with 1,2‐distearoyl‐sn‐glycero‐3‐phosphoethanolamine‐*N*‐methoxy(polyethylene glycol)] copolymer (DSPEmPEG). Five different PEG chain lengths have been used for comparison. The T_2_ relaxivity of the 14 nm SPIONs increased by 2.54‐fold when the PEG molecular weight decreased from 5000 to 1000 Da; however, it did not increase further when the PEG size further decreased to 750 and 550 Da. The change is even more significant for the 5 nm SPIONs; their T_2_ relaxivity increased by 7.79‐fold when the PEG molecular weight decreased from 5000 to 550 Da. Interestingly, both cores have a critical PEG size, at which the T_2_ relaxivity changed dramatically. With 14 nm core and DSPE‐PEG1000, SPIONs display a T_2_ relaxivity of 385±39 s^−1^ mm ^−1^, which is among the highest per‐Fe atom relaxivities of all SPIONs reported.

**Figure 21:**
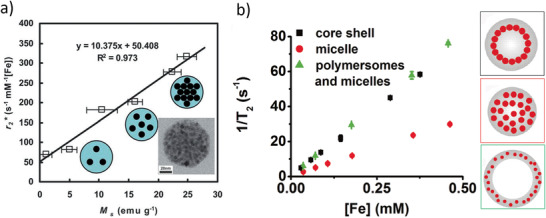
Effect of the loading and arrangement of the magnetic NPs in the composites on the transverse relaxivity. a) Reproduced with permission.^[^ ^464^ ^]^ Copyright 2011, The Royal Society of Chemistry. b) Reproduced with permission.^[^ ^456^ ^]^ Copyright 2011, American Chemical Society.

In a different approach, the development of colloidal organic–inorganic nanocomposites made of multiple subunits arranged in a controlled topological fashion could lead to enhanced relaxivities. Polymers,^[^ ^232^, ^460^ ^]^ block‐copolymers,^[^ ^456^ ^]^ polyelectrolytes,^[^ ^461^ ^]^ and micelles^[^ ^230^, ^462^ ^]^ act as both stabilizers and as templates for the assembly of the magnetic subunits. The control assembly is either the result of direct specific interactions (e.g., van der Waals attractions, steric repulsions, attractive depletion or capillary forces, Coulomb forces, etc.) of their discrete components,^[^ ^436^, ^453^ ^]^ or can be carried out by applying an external stimulus.^[^ ^463^ ^]^ The role of the magnetic particle loading and the type of the assembly on the saturation magnetization and the transverse relaxivity is illustrated in **Figures** **21**a and 21b, respectively.^[^ ^456^, ^464^ ^]^ Αssemblies of oriented maghemite NPs capped with OAc and varied intracluster magnetic material volume fraction exhibit among the highest transverse relaxivities in a magnetic field of 1.5 T (≈ 510 mm ^−1^s^−1^) due to their strong intraparticle interactions.^[^ ^232^ ^]^

**Figure 22:**
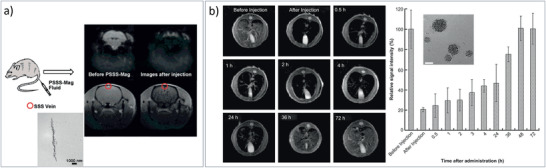
Magnetic nanocomposites in brain (a) and liver (b) MRI imaging. a) Reproduced with permission.^[^ ^461^ ^]^ Copyright 2008, American Chemical Society. b) Reproduced with permission.^[^ ^465^ ^]^ Copyright 2009, Elsevier.

Furthermore, chain‐like assemblies of iron oxide NPs formed by using a negatively charged polyelectrolyte and directed by an external field have been evaluated for the imaging of the rat‐brain (**Figure** **22**a),^[^ ^461^ ^]^ while micellar nanocomposites of manganese ferrite NPs can be delivered in the region of the liver (Figure 22b).^[^ ^465^ ^]^ The latter can contribute to the identification of small lesions and normal tissues, evaluation of the degree of liver cirrhosis, and to differential diagnosis of other liver diseases. This clustered nanocomposite yielded significant liver contrast with signal intensity changes of ≈80% at 5 min after intravenous administration. The time window for enhanced‐MRI can last about 36 h with obvious contrast on liver images.

##### Potential and Limitations for Nanocomposite‐Based MRI Contrast Agents

MNCs exhibit improved relaxometric properties compared to the individual superparamagnetic nanocrystals; however, there are issues that restrict their use in clinical practice. In particular:

a) The total particle size and the biosafety of MNCs are important features for their potentiality as MRI contrast agents. The clustering of small magnetic nanocrystals in larger structures or the combination of two materials in a single particle seems an effective way to increase the magnetization and improve the relaxometric properties of the final product. Despite this, the enlargement of the magnetic particle size could cause a strong EPR action. They could get accumulated in the reticuloendothelial system for a long time, which can cause severe toxicity.^[^ ^466^ ^]^
b) Biocompatibility, bio‐distribution and accumulation, bio‐degradation, defensive and inflammatory responses, and metabolism/clearance mechanisms are important to be explored for these contrast agents. In vitro models, as well studies using animal models are essential to determine the safe concentration and dosage of the new systems. Furthermore, studies using proteomics, genomics, and metabolomics could be very useful to understand the underlying mechanisms and predict the toxicity in different types of cells.^[^ ^467^ ^]^
c) Although small scale production to generate the required magnetic nanocomposites is sufficient for laboratory and clinical trials, a reproducible production also in larger scales is still required for economical industrialization and subsequent commercialization.^[^ ^455^ ^]^

#### 4.4.2 Magnetic Nanocomposites as Effective Agents for Multimodal Imaging

In many cases, the single imaging modality cannot provide complete diagnostic information. Combination of imaging modalities, for example, MRI together with optical fluorescence, computer tomography (CT), and positron emission tomography (PET) could lead to more sensitive and accurate diagnosis.^[^ ^469^ ^]^ Different information regarding the same region of interest can be provided by a single injection of a contrast composite‐based agent. Magnetic nanocomposites can be multifunctional contrast agents which enable multiple imaging modalities at the same time due to their multi‐phase structure. The role of introducing magnetic nanocomposites for multimodal imaging is described in this section.

##### T_1_‐T_2_ MR Imaging Agents

**Figure 23:**
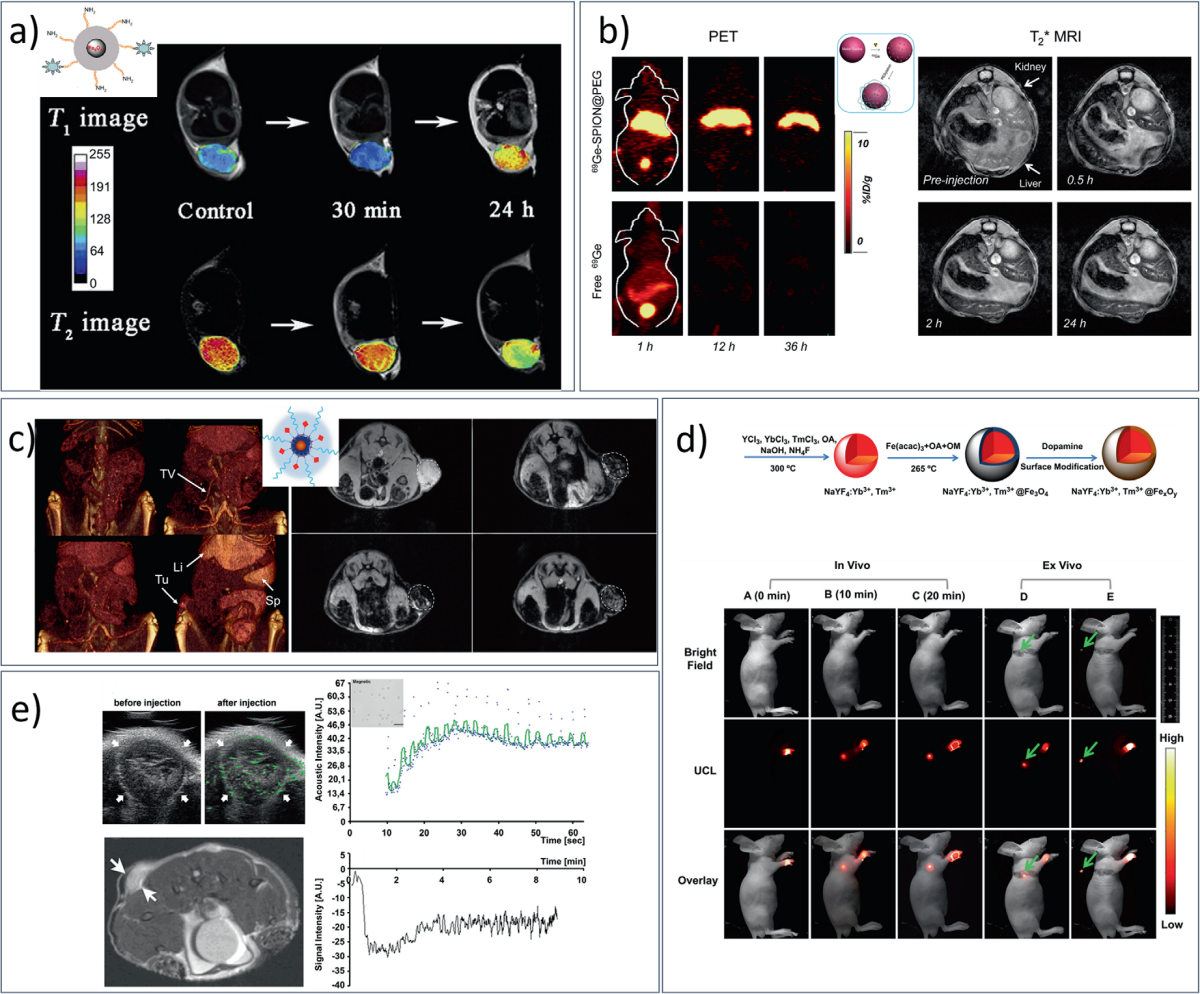
Magnetic nanocomposites as effective multi‐modal imaging probes such as T1‐T2 MR Imaging agents (a) radionuclide‐MR imaging agents (b) CT‐MR imaging agents (c) optical‐MR imaging agents (d) and ultrasound‐MR Imaging agents (e). a) Reproduced with permission.^[^ ^473^ ^]^ Copyright 2011, Elsevier. b) Reproduced with permission.^[^ ^477^ ^]^ Copyright 2014, Wiley‐VCH. c) Reproduced with permission.^[^ ^487^ ^]^ Copyright 2012, American Chemical Society. d) Reproduced with permission.^[^ ^496^ ^]^ Copyright 2011, Elsevier. e) Reproduced with permission.^[^ ^497^ ^]^ Copyright 2011, Elsevier.

The T_1_‐T_2_ MRI contrast agents enhance the contrast in the regions of interest by bright and dark signals, respectively. An approach to create a T_1_‐T_2_ MRI contrast agent is to use single phase^[^ ^469^ ^]^ or multi‐phase^[^ ^470^ ^]^ magnetic NPs which are effective T_2_‐contrast agents and to incorporate paramagnetic metal ions such as Cd^3+^ and Mn^2+^. The important issue in the design of such dual contrast agents is that the direct contact between the two components has to be avoided. This has to be taken into account because the strong magnetic field generated from T_2_ contrast agent building block can disturb the T_1_ relaxation efficiency. This problem can be effectively solved by introducing new complex materials, such as magnetic nanocomposites, where the two building blocks responsible for the MRI are separated by a long chain^[^ ^469^ ^]^ or SiO_2_ layer (**Figure** **23**a).^[^ ^471^, ^472^, ^473^, ^474^ ^]^

MnFe_2_O_4_ NPs functionalized with folic acid and labeled by gadolinium complexes have been found to not only improve surrounding water proton signals on the T_1_‐weighted image, but also induce significant signal reduction on the T_2_‐weighted image.^[^ ^469^ ^]^ Furthermore, superparamagnetic nanocomposites in which the magnetic part is well separated from the paramagnetic continuous Gd‐based shell with a SiO_2_ layer have shown significant improvements in MR‐imaging.^[^ ^471^, ^472^ ^]^ Image artifacts have been improved^[^ ^471^ ^]^ and “ON” states in both imaging modes were found^[^ ^472^ ^]^ by utilizing such magnetic nanocomposites. The Gd(III) complexes can be also doped in the SiO_2_ shell of the magnetic core–shell architectures.^[^ ^475^ ^]^

In a different approach, Mn‐based systems like manganese oxide (MnO) have been studied for T_1_ contrast enhancement due to the 3d^5^ electronic configuration and high relaxation rate. For instance, in a Janus configuration, MnO can play the role of T_1_ contrast agent, whereas the magnetic component which boosts T_2_ contrast consists of MnFe_2_O_4_.^[^ ^476^ ^]^

##### Radionuclide‐MR Imaging Agents

The synergistic combination of PET (with high sensitivity) and MRI (with high resolution and exquisite soft tissue contrast) has attracted tremendous interest over the last decade. Magnetic nanocomposites can be used for such purposes as dual modality PET/MRI probes. These imaging agents require the development of organic ligands that are able to make the magnetic counterpart lipophilic, and then the material is entrapped into a polymeric matrix. Afterward, these MNCs could act as a targetable water soluble nanocarrier. This nanocarrier would be further functionalized with an appropriate labeling agent (e.g., a positron emitter), to allow multi‐modal imaging. SPION@PEG nanocomposites have been studied for this purpose.^[^ ^477^ ^]^ The label agent in that approach is ^69^Ge which can be incorporated into the SPIONs via a fast and highly specific chelator‐free approach. Non‐invasive PET/MR dual‐modality sentinel lymph node mapping has been demonstrated using these MNCs (Figure 23b).^[^ ^477^ ^]^ Besides, ^68^Ga‐labeled magnetic nanocomposites for MRI/PET imaging have been fabricated by encapsulated magnetic NPs in polymers^[^ ^478^ ^]^ or specific amphiphiles.^[^ ^479^ ^]^ The latter have been used for prostate‐specific membrane antigen targeting, which is a well‐known biomarker of prostate cancer.

##### CT‐MR Imaging Agents

CT is the one of the most common imaging techniques used in clinics.^[^ ^468^ ^]^ Compared with MRI, CT provides high temporal resolution and enables the imaging of various organs, including hard tissues (e.g., bones) and lung. Nowadays, iodinated compounds are available as injectable contrast agents for CT. A difficult issue in the fabrication of CT‐MRI contrast agents is that due to the lower sensitivity of the CT compared to the MRI, the amount of the iodine must be much larger than that of iron oxide in the case of the MRI contrast agents.^[^ ^468^ ^]^

Magnetic composite design composed of radiopaque elements and magnetic NPs can be an effective approach for bimodal CT‐MRI probes. In such nanocomposites, the non‐magnetic component is a metal. Phantom CT imaging demonstrated a good X‐ray contrast which can be attributed to the presence of the metallic part. Magnetic composite platforms possessing a microsphere morphology can include magnetic and metallic nanocrystals (gold nanorods and magnetic clusters)^[^ ^480^ ^]^ or in a different hybrid structure the metallic nanocrystals are decorated on magnetic particles.^[^ ^481^ ^]^ In addition, dumbbell‐like,^[^ ^482^, ^483^ ^]^ core–shell,^[^ ^484^, ^485^ ^]^ and Janus^[^ ^166^, ^486^ ^]^ structures have been tested for their multimodal imaging capability. Specifically, microspheres containing gold nanorods inside as radiopaque elements have been proved to act as ideal drug carriers for selective intra‐arterial catheter‐directed administration to liver tumors while also permitting at the same time the MRI/CT visualization for patient‐specific confirmation of tumor‐targeted delivery.^[^ ^480^ ^]^ In vivo MRI/CT imaging was used to monitor intra‐hepatic distribution and confirm delivery to the targeted tumor regions following catheter‐directed infusion. In a similar manner, dumbbell‐like hybrid structures have been shown to operate as efficient multimodal contrast agents for the accurate diagnosis of the hepatoma.^[^ ^482^ ^]^ Transmigration study of the magneto‐plasmonic core–shell nanocomposites using an in vitro blood–brain barrier (BBB) model demonstrated enhanced transmigration efficiency without disrupting the integrity of the BBB, and showed potential to be used for brain diseases and neurological disorders.^[^ ^484^ ^]^

Multimodal imaging has been also achieved by using materials different than metals. TaO_x_‐based contrast agents were found to be promising for CT and for clinical applications thanks to their reduced cost. Fe_3_O_4_@TaO_x_ core–shell nanocrystals have been reported for such applications.^[^ ^487^ ^]^ When these nanocomposites were intravenously injected, the tumor‐associated vessel was observed using computed CT combined with MRI and revealed the high and low vascular regions of the tumor (Figure 23c).^[^ ^487^ ^]^ Newly formed blood vessels to the tumors can be clearly imaged by CT, whereas the tumor microenvironment, including the hypoxic and oxygenated regions, can be visualized using MRI. These core–shell nanocrystals have enormous potential for accurate cancer diagnosis through visualization of developed tumor vessels, monitoring of tumor status, and anticipation of therapy. In another work, Fe_3_O_4_@ZrO_2_ core–shell nanocomposites were developed using an outside‐to‐inside way, where multiple Fe_3_O_4_ NPs grew inside the cavity of mesoporous hollow ZrO_2_ nanospheres. These composites exhibited not only superior magnetic properties and CT/MR imaging ability but also high drug loading capacity.^[^ ^488^ ^]^

##### Optical‐MR Imaging Agents

Optical imaging is a technique which is based on the detection of the light. Fluorescent organic dyes, quantum dots, or upconversion NPs have been included in magnetic composites in order to act as effective multimodal probes. This imaging revealed some advantages, which are unique in the case of the cancer surgery.^[^ ^489^ ^]^ In particular, the increasing availability of novel, fluorescent composites to identify crucial landmarks including tumor margins, lymph nodes, and vital structures such as nerves, lymphatics, and ureters, can expand the surgeons’ visual capability beyond that of white‐light reflectance.

Multimodal contrast agent consisting of an optically detectable near‐IR fluorescent fluorochrome (Cy5.5 fluorescent dye) conjugated to a MRI‐detectable iron oxide core might offer a novel approach to the surgical resection of brain tumors.^[^ ^490^ ^]^ Furthermore, silica matrix has been used for coverage of such complex structures to protect the organic dyes from photobleaching and improve their photostability.^[^ ^491^ ^]^ Such type of core–shell nanocomposites have been used for human stem cell labeling.^[^ ^492^ ^]^

Semiconducting nanocrystals have been incorporated as well in magnetic nanocomposites for optical imaging. Quantum dot‐capped magnetite nanorings were evaluated as a new class of magnetic‐fluorescent nanoprobes for bladder cancer cells.^[^ ^493^ ^]^ These magnetic nanorings have shown a r^2^* relaxivity 110 times larger than that of the commercial compound Resovist. The multiphoton fluorescence imaging and cell uptake of QD‐ capped magnetite nanorings are also demonstrated using MGH bladder cancer cells indicating their effectiveness in the intracellular imaging. These magnetic nanocomposites can escape from endosomes and be released into the cytoplasm. Similar fluorescent‐MNCs have been proposed in a novel method to visualize and quantify lipoprotein uptake kinetics in vivo by MRI in real time.^[^ ^494^ ^]^ These nanocomposites have been fabricated by embedding quantum dots and superparamagnetic iron oxide nanocrystals in the core of lipoproteins (micelles that transport lipids and other hydrophobic substances in the blood) and showed that it is possible to image and quantify the kinetics of lipoprotein metabolism in vivo using fluorescence and dynamic MRI. Using this strategy, it is possible to study the clearance of lipoproteins in metabolic disorders and to improve the contrast in clinical imaging.

Furthermore, upconversion nanocrystals have been utilized as fluorescent counterparts in magnetic nanocomposites for optical imaging. These NPs emit higher‐energy visible light when excited by NIR light.^[^ ^468^ ^]^ The magnetic particles together with the fluorescent ones have been encapsulated in an amphiphilic block copolymer.^[^ ^495^ ^]^ Alternatively, the upconversion material could cover the magnetic one forming a core–shell morphology (Figure 23d).^[^ ^496^ ^]^ NaYF_4_:Yb^3+^, Tm^3+^@Fe_x_O_y_ core–shell nanocomposites were proposed to help guide clinical lymph nodal study and diagnosis without skin surgery (Figure 23d).^[^ ^496^ ^]^

##### Ultrasound‐MR Imaging Agents

Ultrasound (US) imaging is based on the difference in the ultrasound passage rate through different tissues.^[^ ^497^ ^]^ Iron oxide NPs embedded in polymeric microbubbles were designed as multi‐modal contrast agents for hybrid MR–US imaging.^[^ ^497^ ^]^ These hybrid imaging agents exhibited strong contrast in US and increased transversal relaxation rate in MR. Moreover, a significant increase in longitudinal and transversal relaxivities was observed after US‐induced bubble destruction, which demonstrated triggerable MR imaging capabilities (Figure 23e).^[^ ^497^ ^]^ In addition, biodegradable magnetic yolk–shell morphologies consisting of poly(*γ*‐glutamic acid)‐stabilized Fe_3_O_4_ nanoclusters as magnetic core and disulfide cross‐linkage poly(methacrylic acid) as functional shell have also been designed to be used as hybrid MR–US imaging agents.^[^ ^498^ ^]^

Magnetic‐metallic nanocomposites have been also evaluated as novel probes for MRI and photoacoustic imaging (PAI).^[^ ^499^, ^500^, ^501^, ^502^ ^]^ Nanocomposites composed of hollow gold nanospheres as cores and superparamagnetic iron oxide NPs as satellites have been found to exhibit strong magnetic and NIR absorption properties.^[^ ^499^ ^]^ Taking advantage of those excellent dual types of properties, such nanocomposites were applied for targeted MRI and PAI of cancer cells.

Multi‐modal contrast agents have been designed by combining counterparts effective for ultrasound, photoacoustic tomography, and MR imaging as well. Photoacoustic tomography (PAT) is a hybrid‐imaging modality which has been developed by combining US and optical imaging. PAT images are constructed by detecting the ultrasonic wave that results from the thermo‐elastic expansion of a target through the absorption of light.^[^ ^468^ ^]^ For that purpose, multifunctional microcapsules have been fabricated by loading iron oxide NPs into polylactic acid capsules followed by functionalization of the surface with graphene oxide.^[^ ^503^ ^]^

##### Potential and Limitations for Multimodal Nanocomposite‐Based Imaging

This new imaging technology which takes advantage of different imaging modalities using only a single injection of multifunctional nanocomposite‐based contrast agent could provide complementary information for the diseased areas. Besides the plethora of multimodal materials with satisfactory properties in the laboratory scale, the effective transfer from the laboratory environment to the clinical practice is challenging. In particular, there are certain issues that need to be addressed:

a) Scale up and reproducible production of stable contrast agents with physical properties suitable for multimodal imaging.
b) Issues related to the biocompatibility, toxicity, targeting efficacy, and long‐term stability of these new systems have to be addressed using in vitro or in vivo models. These models will be more complicated for nanocomposite systems where two or more materials with different features are included.
c) In vivo and in vitro bio‐distribution and accumulation, bio‐degradation, defensive and inflammatory responses, and metabolism/clearance mechanisms have to be explored for these nanoprobes.
d) Instrumental design, imaging protocols, and hardware have to be evaluated for this new technology^[^ ^504^ ^]^ in order to obtain high sensitivity and temporal and spatial resolution.

#### 4.4.3 Magnetic Nanocomposites as Effective Mediators for Magnetic Hyperthermia

**Figure 24:**
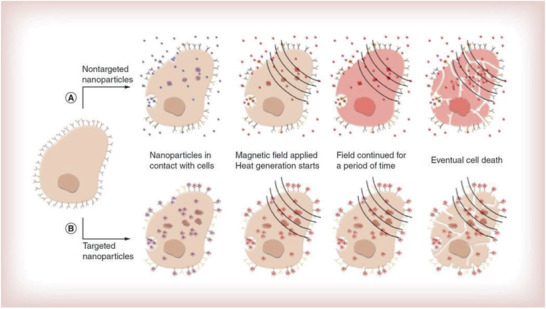
a) Nontargeted magnetic NPs reach the desired cancerous tissue; an alternating magnetic field is applied and nanoparticles dissipate heat; heat dissipation leads to an increase in the temperature of the surroundings reaching hyperthermia levels; and cell death eventually occurs (cell breakage is for illustrative purposes). b) Targeted NPs are internalized due the presence of a targeting ligand through vesicles, endosomes and lysosomes; an alternating magnetic field is applied only to cells with internalized nanoparticles; and cell death can occur through various mechanisms. A temperature rise may not be observed in the bulk. Reproduced with permission.^[^ ^505^ ^]^ Copyright 2013, Future Medicine Ltd.

Magnetic particle hyperthermia presents a promising therapeutic approach for the selective apoptosis of tumor cells through controlled heating of the infected tissues (**Figure** **24**).^[^ ^505^ ^]^ Treatment of tumor regions at temperatures between 41 and 47 °C allows tumor cell destruction while sparing healthy tissue. Heating process can also improve the efficacy of different chemotherapeutic drugs. The combination of conventional chemotherapy with magnetic or a photo‐triggered hyperthermia provides synergistic therapeutic effects and leads to the effective reduction of the anticancer drugs doses.

Τhe heat generation mechanisms can be attributed to two different phenomena, the magnetic moment relaxation and the hysteresis loss, which take place simultaneously. Néel (spin rotation) and Brown (whole particle rotation) are the two types of the relaxation. A way to optimize heating efficiency compared to single‐phase individual magnetic NPs is to form MNCs. In the case of the magnetic nanocomposites where two or more different types of particles are incorporated, the magnetic anisotropy of the final structure as well as the microscopic mechanisms (additional hysteresis loss mechanisms if the nanocomposites are ferro‐ or ferri‐magnetic) involved among the correlated particles may play an important role in their magnetic state and consequently on the heat dissipation.

Magnetic composites which are assemblies of single‐phase magnetic NPs have attracted a strong motivation for their study as potential heat mediators in magnetic hyperthermia.^[^ ^443^, ^506^, ^507^, ^508^, ^509^, ^510^, ^511^, ^512^, ^513^, ^514^ ^]^ A specific loss power (SLP) value of 1175 Wg^−1^ has been found for MNCs incorporating maghemite NPs under an alternating magnetic field of 21.5 kAm^−1^ (at a frequency of 700 kHz).^[^ ^507^ ^]^ The stoichiometry of the incorporated NPs was found also to have a strong impact on the heat dissipation of the final assembly‐like nanocomposites. Stoichiometric spinel ferrite magnetic nanocomposites have displayed an enhanced heating efficiency compared to those of non‐stoichiometric nature.^[^ ^511^ ^]^ Amine capped or polymer‐capped assembly‐like nanocomposites have exhibited a successful ability to kill cancer cells (≈74–90% dead cells)^[^ ^510^ ^]^ even in a very short time (within 15 min).^[^ ^511^ ^]^ Τhe SLP value has been found to be enhanced compared to that of individual NPs when superparamagnetic assembly‐like nanocomposites were capped with amine,^[^ ^507^ ^]^ while a reduced value was observed for superparamagnetic nanocrystals embedded in polystyrene nanospheres.^[^ ^515^ ^]^ A study of these two types of different magnetic nanocomposites revealed that the Néel relaxation is the leading mechanism together with the hysteresis losses for hyperthermia heating in such complex structures.

The dissimilar variation of the SLP upon the applied magnetic field, being proportional to magnetic field strength, H, for the amine capped magnetic nanocomposite^[^ ^507^ ^]^ and to H^2^ for the polystyrene‐encapsulated one,^[^ ^515^ ^]^ revealed that the interactions among NPs are mainly present in the first case. The magnetic interaction among the NPs in the nanocomposites together with the emerging collective properties (occurrence of ferrimagnetism) can lead to enhanced heating response.^[^ ^443^ ^]^ The study of the SLP value of ferrimagnetic nanocomposites with different magnetic material volume fractions indicated a rather complex behavior.^[^ ^231^ ^]^ The SLP value was increased with the size of the nanocomposite and the magnetic material volume fraction (*φ*); however, it decreases for structures larger than 50 nm. The thermal response in these cases was mainly dominated from the hysteresis losses.^[^ ^231^ ^]^

Enhanced heating response was evidenced when metallic NPs are included in the magnetic nanocomposites. The presence of gold leads to a remarkable enhancement by three times of the magnetic‐heating capacity of the nanocomposites.^[^ ^516^ ^]^ This enhancement is highly dependent on the synthesis conditions and the growth mechanism of the metallic particles. Magnetic nanocomposites decorated with fluorescent quantum dots or fluorescent dyes which emit in the visible range of the electromagnetic spectrum can achieve imaging after being administrated in vivo.^[^ ^517^, ^518^ ^]^

Moreover, utilizing a magnetic nanocomposite of iron oxide NPs in hydroxyapatite could help to tackle the low effectiveness of the superparamagnetic NPs in magnetic hyperthermia due to: a) the low uptake in cancer cells, b) the generation of reactive oxygen species that cause harm to the healthy cells, c) the potentiality of new target species, and d) the lack of temperature sensitivity between cancer cells and healthy cells.^[^ ^519^ ^]^ These nanocomposites retain the superparamagnetic nature of their nanocrystal components and showed an increased uptake ratio between cancer cells (U87 human glioblastoma cells) and healthy ones (human mesenchymal stem cells‐MSCs).^[^ ^519^ ^]^ Another interesting approach for effective magnetic hyperthermia is the use of biocompatible magnetic gels composites. Magnetic NPs can be incorporated in thermo‐responsive cross‐linked polymeric hydrogels.^[^ ^520^ ^]^ Mainly due to the hysteresis losses from the magnetic particles subjected to an external magnetic field at fixed frequency and amplitude, the temperature of the system increases and once it crosses the lower critical solution temperature, thermo‐responsive hydrogels undergo large contraction. Such collapse transition can be accompanied by the controlled release of anti‐cancer drug molecules that have been previously entrapped in the gel networks. Besides this, controlled drug release of the DOX molecules have been observed also in magnetic nanocomposites using a pH‐ and thermo‐responsive polymer.^[^ ^521^, ^522^ ^]^ The drug release experiments confirmed that only a small amount of DOX was released at room temperature and physiological pH, while the highest drug release of 85.2% was obtained after 48 h at acidic tumor pH under hyperthermia conditions (50 °C).^[^ ^521^ ^]^

All‐inorganic nanocomposites in core–shell^[^ ^31^, ^523^, ^524^, ^525^ ^]^ or more complex structures such as dimer^[^ ^181^, ^526^ ^]^ or Janus^[^ ^527^ ^]^ architectures were tested also for their efficiency as heating mediators in magnetic hyperthermia. Bimagnetic^[^ ^31^, ^523^, ^524^, ^525^ ^]^ or magnetic‐silica^[^ ^528^, ^529^ ^]^ is the most well‐studied core–shell morphology.

Magnetic hyperthermia measurements indicated a large increase in SAR for the bimagnetic Fe_3_O_4_/CoFe_2_O_4_ compared to Fe_3_O_4_ of the same size, and also this value is raised further with the increase of the thickness of the CoFe_2_O_4_ cell.^[^ ^523^ ^]^ The effective anisotropy of these structures can be tuned by the substitution of Co^2+^ by Zn^2+^ ions in the shell.^[^ ^524^ ^]^ The magnetic anisotropy can be systematically decreased as the Zn concentration increases. Magnetic hyperthermia experiments indicated that the dominant heating mechanism of the samples with higher anisotropy is the Brown relaxation, while the predominant heating mechanism in the samples with lower anisotropy is the Néel relaxation. In core–shell *α*‐Fe@Fe_x_O_y_ nanocomposites, the preservation of the high crystallinity of the Fe core leads to significantly higher magnetic hyperthermia outcomes.^[^ ^31^ ^]^ 18 nm particles retain their chemical properties (no sign of chemical deterioration) over 2 months, with lower oxidation of the Fe core resulting in a SAR value of 660 W g^−1^ and intrinsic loss power (ILP) of 3.6 nHm^2^ kg^−1^, while the 15 nm nanocomposites demonstrated a reduction of their properties due to their core oxidation. In addition, an interesting study combining electron microscopy and data acquired from high‐energy synchrotron facilities, such as X‐ray total scattering measurements, indicated that Fe_x_O−Fe_3−*δ*_O_4_ core–shell structures could lead to single‐crystal nanoscale entities with subdomain Fe_x_O−Fe_3−*δ*_O_4_ interfacial connectivity and extended defects with heat dissipation almost tenfold enhanced compared to single phase nanocrystals.^[^ ^525^ ^]^ Finally, it has been illustrated that in FePt@iron oxide core–shell nanocomposites, the FePt core can be exchange‐coupled with the iron oxide shell, resulting in a significant enhancement of the SAR value.^[^ ^530^ ^]^ In this case, the presence of the FePt core maintains chain‐like assemblies during AC field pulses in MRI measurement, which essentially increases the R_2_.

Magnetic‐metallic dimers,^[^ ^181^, ^526^, ^531^ ^]^ Janus,^[^ ^527^, ^532^, ^533^ ^]^ or yolk–shell^[^ ^534^ ^]^ particles have been developed with the aim to enhance the hyperthermic efficiency and to provide a photo‐triggered thermal treatment. The coexistence of two different materials, for example, Au and iron oxide, in the same composite could achieve selective surface attachment on the two different domains, thus allowing controlled targeting and drug delivery. The presence of the metallic part enhances the efficiency in magnetic hyperthermia. As demonstrated by Pellegrino's group, the synthesis method for Au‐Fe_x_O_y_ dimers affected the final morphology of the dimers and consequently their efficiency.^[^ ^181^ ^]^ They used a “‘one‐pot”’ approach where the Au nanocrystals were first nucleated in situ in the presence of chloride ions followed by the growth of the iron oxide domain. A “‘two‐pot”’ approach was also employed for comparison, in which pre‐synthesized Au NPs were used as seeds, leading to nanocrystals of larger magnetic part (>20 nm) which are more effective for such applications. It was also shown that the absence of 1,2‐hexadecanediol, a commonly used surfactant in the synthesis, favors the growth of dimers with larger iron oxide domains.

**Figure 25:**
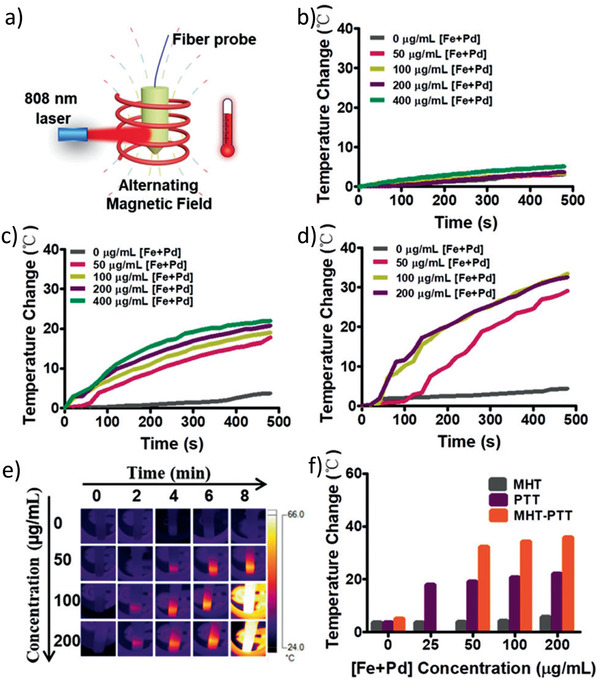
Magnetic‐photo dual‐mode hyperthermia in aqueous solution by using Pd‐Fe_3_O_4_ Janus nanocomposites. a) Schematic diagram of the magnetic‐photo dual‐mode hyperthermia measurement system. Time dependent heating profiles at various concentrations under AMF (300 Oe; 350 kHz) (b), under 808 nm laser irradiation at a power density of 0.5 W cm^−2^ (c), and under AMF plus 808 nm laser (d). Thermal images acquired by the IR camera of Fe_3_O_4_–Pd JNPs at different concentrations under AMF plus 808 nm laser (e). Average temperature increase recorded under the three heating protocols (f). Reproduced with permission.^[^ ^527^ ^]^ Copyright 2019, Royal Society of Chemistry.

The combination of spherical Fe_3_O_4_ nanocrystals on Pb nanosheets in a Janus architecture was delivered by intravenous administration to an orthotopic 4T1 breast tumor and tested as mediator for amplified dual‐mode magnetic‐photo‐hyperthermia treatment under the proper guidance of dual‐modality imaging.^[^ ^527^ ^]^ Enhanced ROS generation was noticed after alternating magnetic field plus NIR laser irradiation exposure in vitro, whereas amplified therapeutic effects for magnetic‐photo heating in vivo were observed (**Figure** **25**). The tumor was completely eliminated on day 18, as monitored under MRI/PA dual‐mode imaging with a high spatial resolution and accuracy.^[^ ^527^ ^]^ In a different strategy, a “non‐contact” incorporation of metallic nanocrystals in a magnetic nanoshell forming a yolk–shell morphology allows the integration of discrete functionalities, which is attractive for incorporating plasmonic materials in a “well‐protected” manner, which is intrinsically different from previous direct chemical or heterogeneous conjugation of the two components.^[^ ^534^ ^]^ The “non‐contact” incorporation of gold nanorods within iron oxide shell via yolk–shell architecture, realized highly preserved plasmonic cores and successive/permeable magnetic shells allowing MRI/PAI dual‐modal tumor diagnosis, photothermal therapy, and DOX delivery. This nanocomposite exhibited distinct NIR plasmonic absorption and superior magnetic responsiveness, together with pH/local heating dual‐responsive drug release behavior owing to the special coordination between DOX and iron species.^[^ ^534^ ^]^ Eventually, upconversion nanocrystals in combination with a magnetic part (MnFe_2_O_4_‐NaYF_4_) in a Janus configuration have been evaluated as an upconversion luminescence imaging agent, with promising performance in photothermal treatment. This composite displayed a strong absorption in the NIR region and can convert NIR light into heat after 808 nm laser irradiation.^[^ ^164^ ^]^

Despite the promising results of utilizing magnetic nanocomposites for heating generation on tumors in magnetic hyperthermia preclinical studies, there are numerous challenges that must be addressed before this treatment can progress to a clinical level. More specifically:

a) Optimization of the intrinsic features of magnetic mediators by modulating their nanocomposite geometry, size of the components, size distribution of the final structures, crystallinity, and compositional tuning of each component in order to enhance the heating efficiency. Once the inherent features in laboratory conditions are optimized, efforts to scale‐up the synthesis, and improve the repeatability of these materials have to be made.
b) Active targeting of the nanocomposites to the tumor by decoration with peptides and antibodies to increase the accumulation of the magnetic particles to the infected areas compared to the normal tissues is essential. Ideally, a high concentration of magnetic particles for high heating efficiency should be localized at the tumor and not accumulate in healthy tissues nor in excretory organs such as spleen, liver, and kidneys. In reality, this does not happen with the intravenous injection of the non‐targeting mediators. The loading of magnetic composites exclusively on tumor regions may not be always a facile process.
c) Issues related to the biocompatibility, toxicity, targeting efficacy, and long‐term stability of these magnetic mediators have to be addressed using in vitro or in vivo models. These models are complicated when two or more different materials with distinct features are included.
d) In vivo and in vitro bio‐distribution and accumulation, bio‐degradation, defensive and inflammatory responses, and metabolism/clearance mechanisms for these materials have to be explored. The optimum dosage and concentration of the nanocomposite‐based mediators have to be evaluated, aiming to obtain the lowest amount of side effects.
e) Development of numerical models for the prediction of the spatiotemporal distribution of the temperature in the living tissues during the therapy with magnetic nanocomposites is required. These models will be more complicated in the case of magnetic nanocomposites in which two or more materials are included.^[^ ^535^ ^]^ The optimum field strength and frequency have to be predicted through these models so as to receive the weakest side effects.
f) There is a need for real‐time monitoring of temperature rise during the magnetic hyperthermia treatment. Temperature uncontrollability occurs due to the thermal conductivity of human tissues, blood perfusion, the movement of organs, and due to inhomogeneous distribution of the magnetic mediators inside the tumors.^[^ ^536^ ^]^

#### 4.4.4 Magnetic Nanocomposites as Effective Therapeutic Agents

MNCs have been introduced in the personalized medicine as targeted theranostic nanoplatforms. More specifically, such nanomaterials have been used for magnetic targeted delivery and stimuli‐responsive release of drugs.^[^ ^537^, ^538^ ^]^ Magnetic nanocomposites of multiple morphologies were used for image‐guided therapy in which different drugs have been delivered and released in specific diseased areas. Magnetic core–shell morphologies, for example, with SiO_2_,^[^ ^446^, ^529^, ^539^, ^540^ ^]^ C,^[^ ^541^ ^]^ ZrO_2_,^[^ ^488^ ^]^ or GO^[^ ^452^ ^]^ in the shell could encapsulate DOX for chemotherapy or brain‐targeted HIV treatment (tenofovir disoproxil fumarate).^[^ ^484^ ^]^

**Figure 26:**
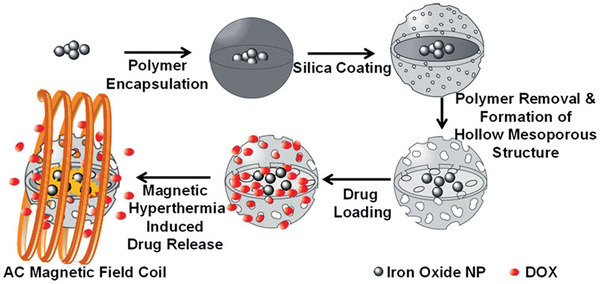
Schematic illustration of the magnetic nanoparticle encapsulation, drug loading and magnetic hyperthermia induced drug release. Reproduced with permission.^[^ ^544^ ^]^ Copyright 2013, Royal Society of Chemistry.

It has been proved that an external magnetic field improves the cellular uptake efficiency of silica‐based nanocomposites.^[^ ^542^ ^]^ Such magnetic composites have been used as a drug delivery vehicle for stimuli‐responsive dosing of therapeutic molecules via applying an alternative magnetic field (**Figure** **26**).^[^ ^543^, ^544^, ^545^, ^546^, ^547^, ^548^ ^]^ Core–shell composites were trafficked into lysosomes, mainly through an energy dependent pathway, namely clathrin‐induced endocytosis. Furthermore, Zn‐doped iron oxide nanocrystals within a mesoporous silica framework surface modified with pseudorotaxanes have been used for controlled drug delivery and cancer killing.^[^ ^549^ ^]^

Upon application of an AC magnetic field, the nanocrystals generate local internal heating, causing the molecular machines to disassemble and allowing the cargos (drugs) to be released. When breast cancer cells (MDA‐MB‐231) were treated with DOX‐loaded particles and exposed to an AC field, cell death occurred. On‐off drug release has been succeeded using a magnetic nanocomposite in which DNA/magnetic NP conjugates cap the pores of the magnetic silica particles upon hybridization of both DNA strands.^[^ ^545^ ^]^ Progressive double‐stranded DNA melting as a result of temperature increase gave rise to uncapping and the subsequent release of a mesopore‐filled model drug, fluorescein. The reversibility of DNA linkage results in such on‐off release mechanism. In a different approach, a thermoresponsive hybrid polymer/magnetic mesoporous silica nanovehicle has been developed to allow the magnetic field‐triggered release of different cargos, proteins, and small molecules housed in the polymer branches and in the silica matrix, respectively, in a controlled manner.^[^ ^547^ ^]^ These complex composites are decorated on the surface with a thermoresponsive copolymer, poly(ethyleneimine)‐*b*‐poly(N‐isopropylacrylamide) (PEI/NIPAM). In addition, very recently, a more complex silica‐based structure (mSiO_2_@PbS/CdS‐Fe_3_O_4_) was designed to integrate NIR photoluminescent PbS/CdS QDs and superparamagnetic Fe_3_O_4_ nanocrystals into a large‐pore silica matrix.^[^ ^550^ ^]^ This nanocomposite combines photoluminescence in the NIR‐II window, enabling its use for deep‐tissue imaging, together with excellent superparamagnetic properties that make it easily confined by an external magnetic field and ideal as a highly efficient T2 contrast agent for MR imaging in vivo. This was achieved thanks to the synergistic magnetic coupling effect induced by the close distance of Fe_3_O_4_ nanocrystals embedded in the mesoporous channel. At the same time, DOX drugs can be loaded and a pH‐responsive drug‐release behavior is shown: mSiO_2_@PbS/CdS‐Fe_3_O_4_ can produce local heat via the magnetothermal effect, due to the presence of superparamagnetic nanocrystals, and accelerate the drug release.

Furthermore, thermoresponsive shells of polymer or hydrogel have been used to cover magnetic NPs in order to operate as both heating and drug carrier agents. For example, the conjugation of DOX to the pH‐ and thermo‐responsive magnetic composites via acid‐cleavable imine linker provides advanced features for the targeted delivery of DOX molecules and offers spatial and temporal control over the drug release.^[^ ^521^ ^]^ Only a small amount of DOX was released at ambient conditions and physiological pH, while the highest drug release of 85.2% was obtained after 48 h at acidic tumor pH under hyperthermia conditions (50 °C). In a similar manner, magnetic nanocomposites comprising magnetic chitosan‐g‐PNVCL polymeric nanogels loaded with DOX were effectively taken up by the cells and 67% of iron content was present inside the cells when subjected to quantification after 24 h.^[^ ^551^ ^]^ Approximately fourfold enhancement of the drug release was observed by utilizing injectable, degradable hydrogel‐thermoresponsive microgel‐SPION nanocomposites.^[^ ^552^ ^]^ These systems can maintain pulsatile release properties over multiple cycles and for several days instead of only hours.

Controlled drug release has been observed in magnetic nanocomposites not only by thermal triggering under exposure to an external magnetic field. Carboxymethyl dextran‐coated magnetoliposomes were fabricated for DOX release.^[^ ^553^ ^]^ In this case, the release of DOX‐loaded magnetoliposomes was enhanced by low‐frequency alternating magnetic field without hyperthermia generation. Furthermore, very recently a core–shell nanocomposite with a superparamagnetic doped ferrite (MnFe_2_O_4_@CoFe_2_O_4_) NP core in a mesoporous silica shell which has a high cargo‐carrying capacity has been designed for drug delivery with a spatial, temporal, and dose control.^[^ ^554^ ^]^ In order to regulate the cargo (fluorescein or DOX) release, a thermoresponsive molecular‐based gatekeeper containing an aliphatic azo group was modified on the core–shell NPs. The release of cargo is due to the removal of the gatekeepers and the amount of the cargo release could be adjusted by the exposure time of the alternative magnetic field. The cargo release can take place in a stepwise manner via multiple sequential exposures of alternative magnetic field increasing the total amount of released cargo without overheating the particles’ surroundings. In another report, a versatile nanocomposite consisting of GO‐iron oxide‐DOX was explored as a theranostic cancer platform that combines hyperthermia and chemotherapy. The localized tumoricidal effects of the proposed GO‐IO‐DOX considerably increased due to the drug sensitization by repeated mild hyperthermia application. The main advantage of using these MNCs in hyperthermia‐based treatment is the synergistic improvement in heating efficiency properties due to the high thermal conductivity of GO.^[^ ^451^ ^]^

**Figure 27:**
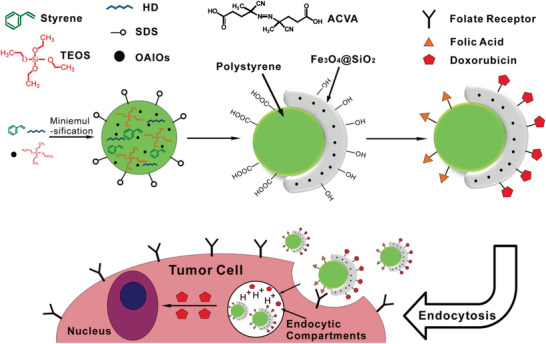
Proposed mechanism for tumor cell targeting and stimulus induced drug release of PS/Fe_3_O_4_@SiO_2_ superparamagnetic Janus nanocomposites. Reproduced with permission.^[^ ^555^ ^]^ Copyright 2013, Wiley‐VCH.

Moreover, the unique features of Janus MNCs which possess multiple surface structures that are anisotropic in morphology, composition, and surface functionalization have been effectively exploited in simultaneous tumor cell targeting and stimulus‐induced drug release.^[^ ^555^ ^]^ Functionally distinct surfaces in Janus morphologies can be selectively conjugated for cell targeting. Superparamagnetic Janus nanocomposites which are composed of a PS core and a half silica shell with iron oxide NPs embedded in its matrix have been developed for such applications. The two different surfaces can be suitable for selective chemical modification using different functional groups. Tumor cell targeting was attempted by the conjugation of folic acid to the PS surface using a bis‐amine linker (**Figure** **27**). The antitumor agent DOX was immobilized to the silica shell via a pH‐sensitive hydrazone bond facilitating stimulus‐induced drug release after internalization under acidic conditions in endosomal compartments. The therapeutic advantage of this novel nanostructure is that only tumor cells will be exposed to high concentrations of the cytotoxic DOX because of the chemical stability of the hydrazone bond during bloodstream circulation at pH 7.4. Consequently, patients are expected to suffer less from side effects that are commonly associated with the systemic circulation of free DOX. In a different work, the “non‐contact” incorporation of gold nanorods into porous magnetic nanoshell in a yolk–shell structure yields a strong superparamagnetic response with excellent permeability for magnetically targeted drug delivery.^[^ ^534^ ^]^ Interestingly, the special coordination between DOX and Fe species enabled pH/local heating dual‐responsive drug release with minor leakage at neutral pH. Under the guidance of dual‐modal imaging and magnetically tumor targeting using the nanocomposites, the photothermal‐chemo synergistic therapy was conducted via near‐infrared laser for highly efficient tumor eradication.

**Figure 28:**
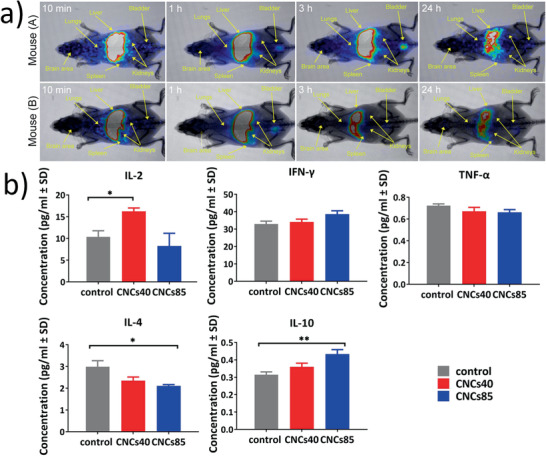
Magnetic nanocomposites accumulated in the liver (static scintigraphy/X‐ray images) (a) and the detection of cytokines in cells (b). Reproduced under the terms of the Creative Commons CC‐BY license.^[^ ^556^ ^]^ Copyright 2018, The Authors, published by MDPI.

Kostopoulou et al. reported the use of assembly‐like nanocomposites as contrast agents in MRI and as treatment agents for immunotherapy. Careful monitoring of the distribution, circulation, and likely excretion of such nanocomposites through the organism helps to infer effectiveness toward their preclinical use as contrast agents for diagnosis of liver lesions (**Figure** **28**a) and for immunotherapy in related diseases.^[^ ^556^, ^557^ ^]^ Although magnetic nanocomposites attain relatively similar MRI capabilities in a range of sizes, nanoclusters of different sizes appear to present a crucially unlike degree or type of interaction at the cellular level. Due to their ability to induce size‐dependent cytokine production (Figure 28b), smaller size nanoclusters might be more effective for therapeutic purposes (increasing the production of the inflammatory IL‐2) where the activation of the immune system is required^[^ ^556^ ^]^. On the other hand, larger nanoplatforms appear potentially promising as immune‐suppressive agents (through the decrease of anti‐inflammatory cytokines IL‐4 and increase of IL‐10).

**Figure 29:**
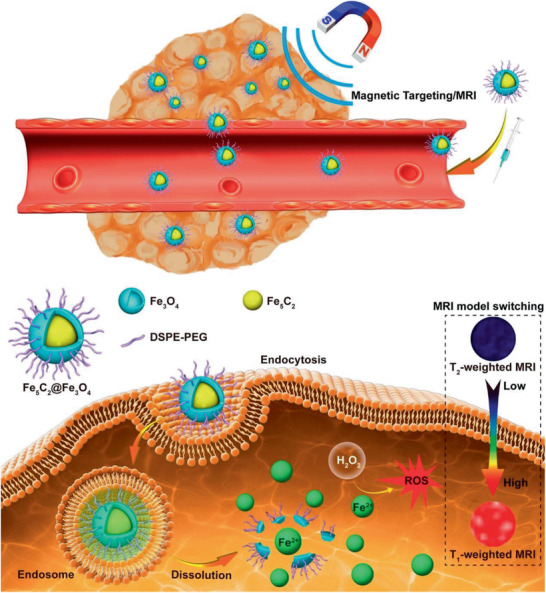
Schematic illustration of Fe_5_C_2_@Fe_3_O_4_ NPs for pH‐responsive Fe^2+^ release, ROS, and T2/T1 signal conversion. Reproduced with permission.^[^ ^558^ ^]^ Copyright 2018, American Chemical Society.

In a different approach, very recently, magnetic nanocomposites were used as magnetic reactive oxygen species nanoreactors for cancer treatment.^[^ ^558^ ^]^ Core–shell Fe_5_C_2_@Fe_3_O_4_ nanocomposites were synthesized for this purpose due to the fact that the Fe_5_C_2_ core is magnetic and the pH‐responsive iron oxide shell can efficiently release the H_2_O_2_ “catalyst” ferrous ions in acidic environments (**Figure** **29**).^[^ ^558^ ^]^ A significant advantage of this nanocomposite compared to single phase Fe_3_O_4_ nanocrystal is that the former is more sensitive to acidity and can discharge ferrous ions more effectively in low‐pH environments. This indicates their ability to catalyze a higher level of ROS generation resulting in better tumor‐selective therapy with low toxicity. Simultaneously, the efficient discharge of iron ions from the magnetic nanocomposites at the tumors results in the decrease of their magnetization and reduction of the T_2_ signal in the MRI. At the same time, the release of T_1_ “signal enhancer” ferrous ions for lightening on T_1_‐weighted MRI is achieved. This T_2_/T_1_ signal conversion sensitively denoted the production of ferrous ions and, consequently, traced the release of ROS for visible cancer therapy. Furthermore, core–shell nanocomposites comprising an iron oxide (core) and cerium oxide (shell) have been synthesized as a potential theranostic nanomaterial for ROS‐related inflammatory diseases. In particular, this composite was targeted for the treatment of rheumatoid arthritis and diseases related to atherosclerotic plaques.^[^ ^559^ ^]^ This innovative material simultaneously provides both diagnostic capability supported by the iron oxide component via MRI imaging and therapeutic functionality thanks to the cerium oxide part through their anti‐ROS capability in one dose.

Summarizing the magnetic nanocomposites tested as possible magnetic delivery systems, a few key challenges must be addressed to realize the maximum potential of these magnetic materials in such applications. Certain features/properties can be identified which are crucial for the successful drug delivery.^[^ ^560^ ^]^ In particular: magnetic composites have i) to be sufficiently large to possess high magnetization for specific magnetic targeting; ii) to exhibit controlled drug uptake and release with well‐defined mechanism; iii) to possess theranostic features in order to improve the delivery efficiency of the drug as well as its action; iv) to be long‐circulating at the bloodstream as long as it is required and not be removed too fast by the reticuloendothelial system.

Targeting can be accomplished by conjugating ligands or antibodies or by using an external magnetic field. The main advantage of the targeted drug delivery is to reduce side effects and to ensure minimal exposure of the circulation of drugs to other parts of the body, thus controlling the effectiveness of the drug on a specific diseased location. Notwithstanding that magnetic targeting is not effective for all the diseased areas, its further development should provide another tool for effective treatment. Cellular recognition, biocompatibility, and drug release or effective dose of the magnetic vehicles are some of the issues that have to be studied in vitro and in vivo. However, limitations associated with the strength of the external magnetic field and issues related to tissue penetration depth have to be further optimized experimentally or through mathematical models.

Furthermore, beside the significant progress made in the field of nanocomposite‐based therapeutic agents, the understanding of particles pharmacokinetics (adsorption, uptake, distribution, metabolism, and excretion) is quite limited till now.^[^ ^561^ ^]^ The pharmacokinetics are more difficult to be predicted through models in the case of multi‐component magnetic nanocomposites. Establishment of clear and detailed toxicity, immunogenicity, clearance, and safety profiles are needed based on in vitro or in vivo studies.

The successful translation of the nanovehicles to the clinic is broadly dependent upon two interrelated main factors: biological and chemical challenges. Moreover, another issue in this translation is the discrepancy between animal and human models, which chiefly influence clinical outcomes.^[^ ^562^ ^]^

All the above problems/challenges are reflected to the fact that considering the substantial preclinical research efforts into targeted drug delivery with magnetic nanoprobes, only a few have been approved for clinical practice, with no use at humans so far.^[^ ^562^ ^]^

#### 4.4.5 Magnetic Nanocomposites for Biomolecule Separation

The separation of specific biomolecules from their native environment for analysis is often required. Superparamagnetic colloids are ideal for this application because of their ability to switch on and off their magnetization according to the presence (or absence) of an external magnetic field enabling the transportation of biomaterials with a magnetic field.^[^ ^563^ ^]^ Magnetic nanocomposites have been extensively used for separation of biomolecules in bioprocesses. The strong magnetic response and high surface area of the MNCs facilitates the biomolecule binding. In fact, separation efficiencies of 99% have been achieved.^[^ ^564^ ^]^

**Figure 30:**
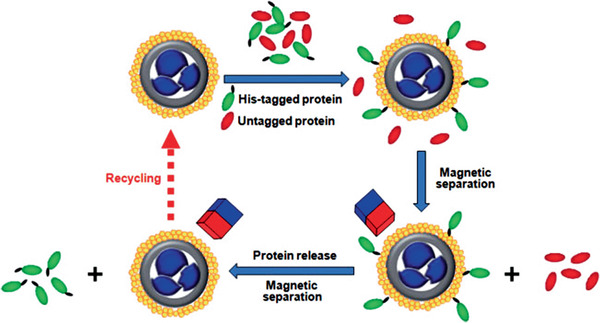
Magnetically recyclable protein separation process using magnetic nanocomposite (porous core–shell hematite‐ SiO_2_ decorated with NiO NPs). Reproduced with permission.^[^ ^566^ ^]^ Copyright 2010, Wiley‐VCH.

Magnetic nanocomposites have been introduced in biomolecule magnetic separations of proteins,^[^ ^565^, ^566^, ^567^, ^568^ ^]^ antigens,^[^ ^569^ ^]^ or plasmid DNA^[^ ^570^ ^]^ due to their unique capability to introduce two or more different materials and also to provide an accessible surface for specific binding. The decoration of a hematite/SiO_2_ nanocomposite with NiO allows the selective separation of polyhistidine through the Ni‐histidine bond. This complex magnetic nanocomposite and magnetic separation process are illustrated in **Figure** **30**.^[^ ^566^ ^]^ Hematite NPs covered with silica shell can become mesoporous after a calcination process. This porous surface is accessible for the growth of NiΟ nanoparticles. The exposed NiO NPs enable the selective adsorption of His‐tagged protein from the mixed‐protein solution, as well as *E. coli* lysate, whereas the magnetic core allows the particles to be separated from the solution by applying an external magnetic field. The selectivity and recyclability of this magnetic nanocomposite for His‐tagged protein were maintained during several separation cycles of the protein. A composite fabricated by loading small (3 nm) nickel nanoparticles onto cellulose filter paper showed a highly resilient and chemically inert interaction with the His‐tagged protein thanks to the surface oxidation of the Ni NPs to NiO. The selective immobilization of the proteins onto the NPs‐doped filter paper nanocomposite was achieved in this way.^[^ ^571^ ^]^ Magnetic nanocomposites having a core–shell morphology with Fe_3_O_4_ in the core and double layered silica around have been utilized for the septavidin protein separation.^[^ ^572^ ^]^ Biotin conjugated easily to the particles through a typical amine functionalization onto the silica surface followed by amide coupling. For the separation and enrichment of low concentration peptides from a complex mixture, a unique Fe_3_O_4_@2SiO_2_@mSiO_2_‐C nanocomposite was fabricated.^[^ ^569^ ^]^ This composite possesses a highly ordered mesoporous structure, large specific surface area, strong hydrophobic property, and mechanical strength as well as excellent pore volume. A low detectable concentration was achieved, together with excellent repeatability and recovery of enriching peptides. These magnetic nanocomposites successfully enriched 2198 endogenous peptides from human serum. In this case, the isolation of peptides and small proteins from complex samples occurs through a size‐exclusive mechanism and due to the hydrophobic interaction between the carbon counterpart and the peptides.

Plasmid DNA was purified using a core–shell *γ*‐Fe_2_O_3_@SiO_2_ magnetic nanocomposite.^[^ ^570^ ^]^ Comparison with commercial SiO_2_‐coated Fe_3_O_4_‐based magnetic beads used for such purification indicated that the *γ*‐Fe_2_O_3_@SiO_2_ core–shell structures exhibited similar purities, and all had similar theoretical plasmid DNA binding capacities. However, the latter structures displayed a faster separation speed and a higher saturation magnetization compared to the commercial ones.

Multifunctional magnetic nanostructures with yolk–shell morphology can realize the selective capture, convenient magnetic separation, and rapid identification of target phosphopeptides by taking advantage of the Fe_3_O_4_ magnetic cores and the selective YPO_4_ affinity shells.^[^ ^573^ ^]^ Practical application of such structures demonstrates that 2678 unique phosphopeptides can be enriched and identified from the digestion of mouse brain proteins.

In line with the growing interest in biomolecular and cell targeting to selectively separate one or more cells and molecules from their medically relevant assemblies, such complex magnetic nanocomposites have been investigated in this context.^[^ ^574^ ^]^ Ferrofluids of superparamagnetic iron‐oxide/silicate/carbon core/shell/crust NPs were highly effective at separating i) biomolecules, ii) bacteria, and iii) eukaryotic cells from solutions, and they also exhibited selectivity in the separation of different types of those entities. More specifically, it was possible to separate cancer versus normal cells, as well as Gram‐positive versus Gram‐negative, and multidrug‐resistant versus laboratory bacterial strains using a very low magnetic field (<100 T m^−1^). This field was much weaker compared to the magnetic field used in conventional separation technology.

The multifunctional magnetic nanocomposites as described in this section are ideal for separation of different molecules due to the coexistence of distinct material sections together with a large surface area for the binding, but till now, there are issues regarding their synthesis and the underlying mechanisms that have to be solved:

i) Although small scale production to generate the required magnetic nanocomposites may be efficient in laboratory scale, a scale up and reproducible production is still required for economical industrialization and subsequent commercialization. Synthesis routes often require multistep processes. Complex devices such as glovebox and Schlenk line involving inert atmosphere are utilized and the capping of these particles with an organic or inorganic shell will take place in a second step. Lab‐scale synthesis may not be often easy nor cost effective for conversion to mass production.
ii) New or improved synthesis strategies have to be explored in order to prevent aggregation or facile oxidation upon air exposure.
iii) Despite the exploitation of a large number of such nanomaterials in various combinations/configurations, the binding mechanisms are not always well understood in order to improve these processes.
iv) Functionalization has to be explored further to improve their composites biotargeting capacity and enhance their physicochemical properties.^[^ ^575^ ^]^

## 5 Summary and Outlook

Magnetic nanocomposites have already demonstrated a pioneering role in several areas as catalysis, water treatment, sensing, and biomedicine. Though efforts for the production of MNCs have focused on finding faster and more straightforward ways to obtain them, this has not been always easy to achieve. Still, the synergistic properties of the MNCs bring often significant benefits and thus are strongly desired, even if their fabrication involves often multi‐step processes. Several strategies for the synthesis of different types of MNCs have been described in this review. Despite that chemistry‐based stages were usually dominant in the synthetic methods, in certain cases physical‐based techniques were also needed in some fabrication stages. Apart from emphasizing on the synergistic properties of the MNCs arising from the simultaneous presence of different components bearing their own properties, we also described extensively the recent advances achieved toward the development of MNCs. It seems that for the future, a trade‐off between simplifying the production processes for the different types of MNCs, together with maintaining a high level of their resulting properties, which ensure a good performance in applications, is a challenging goal that needs to be achieved. Especially those MNCs that display excellent activity in their respective applications need further attention so that their properties are not kept only at laboratory scale. Their production in larger scale and subsequent commercialization, when applicable, are often difficult but worthy objectives.

In what concerns biomedical applications, besides the recent progress and promising results in the design and synthesis of multifunctional magnetic nanocomposites, except of the scaling‐up of the fabrication processes, there are numerous challenges that must be addressed before the successful translation from the proof‐of‐concept and laboratory‐based stage to clinics. Issues related to biocompatibility, toxicity, targeting efficacy, and long‐term stability of these magnetic nanoprobes have to be addressed using in vitro or in vivo models. These models will be more complicated when two or more different materials with distinct features are incorporated but are essential for both diagnostic and therapeutic applications. In addition, studies using proteomics, genomics, and metabolomics could be very useful to understand the underlying mechanisms and predict the toxicity and pharmacokinetics in different types of cells. In vivo and in vitro bio‐distribution and accumulation, bio‐degradation, defensive and inflammatory responses, and metabolism/clearance mechanisms for these materials have to be explored. The optimum dosage and concentration of the nanocomposite used for theranostic applications need to be thoroughly investigated by using numerical models so as to achieve the lowest amount and severity of side effects.

New or improved strategies to increase the accumulation of the magnetic composites by decoration with peptides and antibodies, aiming for a better targeting to the infected areas for magnetic hyperthermia or drug delivery treatments have to be evaluated.

Finally, issues related to the instrumentation related to the imaging or treatment have to be explored. Instrumental design, imaging protocols, and hardware have to be evaluated, so that new imaging technologies will be able to achieve higher sensitivity as well as temporal and spatial resolution. In the case of hyperthermia treatment, there is a need for real‐time monitoring of temperature rise during the treatment process.

We certainly hope that this review will help the readers to understand better the synthesis, the improved properties, and the performance in applications of the MNCs, in a summarized manner. In this way, it will be easier for researchers to improve the synthesis and function of the MNCs afterward.

## Conflict of Interest

The authors declare no conflict of interest.
